# The SLO Hierarchy of Pseudo-Boolean Functions and Runtime of Evolutionary Algorithms

**DOI:** 10.1007/s00453-025-01359-z

**Published:** 2026-03-24

**Authors:** Duc-Cuong Dang, Per Kristian Lehre

**Affiliations:** 1 Chair of Algorithms for Intelligent Systems, University of Passau, Innstr. 33, 94032 Passau, Germany; 2 School of Computer Science, University of Birmingham, Edgbaston, Birmingham, B15 2TT UK

**Keywords:** Landscape analysis, runtime analysis, mutation operators

## Abstract

While some common fitness landscape characteristics are critical when determining the runtime of evolutionary algorithms (EAs), the relationship between fitness landscape structure and the runtime of EAs is poorly understood. Recently, Dang, Eremeev, and Lehre introduced a classification of pseudo-Boolean problems showing that “sparsity” of local optima and the “density” of fitness valleys can be crucial characteristics when determining the runtime of EAs Dang et al. (in Proceedings of the Genetic and Evolutionary Computation Conference. Association for Computing Machinery, New York, NY, USA, GECCO’21, pp 1133–1141, 10.1145/3449639.3459398, 2021c). However, their approach could only classify some classes of pseudo-Boolean functions and thus defined an incomplete hierarchy. We generalise the previous work to a complete hierarchy for all pseudo-Boolean functions, denoted Slo$^\alpha _{\varepsilon ,r}$. The hierarchy is consistent with existing results for the runtime of EAs. The easiest problems are in Slo$^{\alpha }_{\varepsilon }$ for $\alpha =1$ and $\varepsilon =0$. As we increase $\varepsilon$ and decrease $\alpha$, the function class contains more interesting functions, including instances of hard combinatorial optimisation problems and problems perturbed by static noise. For $\alpha =O(1/n)$ and $\varepsilon =1,$ the problem class contains every problem, including problems closed under permutation (No Free Lunch). Problem classes where local optima sparsity exceed fitness valley density are shown to have exponential black-box complexity. We also study how random perturbations of a function can change its classification. E.g., randomly perturbing search points in OneMax with constant probability leads to a problem class that can still be optimised efficiently with appropriately tuned non-elitist EAs.

## Introduction

A central aim in the runtime analysis of evolutionary algorithms is to determine the structural properties of fitness landscapes (or optimisation problems) that are responsible for making them either easy or hard for different classes of evolutionary algorithms. Orthogonally, the aim is also to understand how the design of an evolutionary algorithms impact the runtime on various problem classes.

Droste, Jansen, and Wegener introduced black-box complexity to classify optimisation problems in terms of their hardness for randomised search heuristics [20]. Roughly speaking (see Sect. 2.2 the formal definition), the black-box complexity of a function is defined as the required number of queries to the function values in order to optimise that function. As pointed out by Carola Doerr, black-box complexity has also been studied under the name (randomised) query-complexity [42]. Zhang proved that the randomised query complexity of finding a *local* optimum of pseudo-Boolean functions is $\Theta (2^{n/2}\sqrt{n})$ Zhang [46]. They point out that the problem of finding a local optimum is related to the problem class PLS (polynomial local search) from [36]. However, only guaranteeing a local optimum is insufficient in many optimisation applications.

The black-box (or randomised query) complexity of finding a *global optimum* of pseudo-Boolean functions is $(1+o(1))2^{n-1}$ [20]. However, this negative result is too pessimistic because we can expect real-world optimisation problems to possess some structure. We would therefore like to consider interesting sub-classes of pseudo-Boolean functions. Every pseudo-Boolean function can be expressed as a polynomial [6], hence one could classify functions by the degree of the polynomial representation. However, this classification is too coarse-grained since polynomials of degree one are trivial while polynomials of degree two are already NP-hard (i.e., they contain problems such as Max 2-SAT [6]).

In more empirical evolutionary computation research, there has been significant interest in understanding algorithm behaviour from the perspective of “fitness landscapes” (see e.g.,  [26, 37]), a metaphor introduced to genetics by [44]. Wright described biological evolution as selection and mutation driving species in a rugged field towards increasingly higher “peaks”, where multiple peaks are separated by “valleys”:

“*The problem of evolution as I see it is that of a mechanism by which the species may continually find its way from lower to higher peaks in such a field.*” [44]

In [39] it was demonstrated that the presence of multiple *sub-optimal funnels*, i.e., collections of paths leading to sub-optimal sink nodes in the network of local optima with deteriorating edges removed, in fitness landscapes contributes to lower success for Randomised Local Search and Simulated Annealing.

Recently, Dang, Eremeev, and Lehre introduced a classification of pseudo-Boolean functions called SparseLocalOpt which quantifies formally the relationship between the “sparsity” of non-optimal peaks (local optima) and the “density” of the surrounding valleys [13]. This classification placed several theoretical benchmark functions within the hierarchy and also provided general conditions for non-elitist EAs guaranteeing expected polynomial runtime on sub-classes of the hierarchy for certain parameter values $\alpha$ and $\varepsilon .$ They constructed a particular function BBFunnel which can be optimised efficiently by several non-elitist EAs, while having exponential $(\mu +\lambda )$-elitist black-box complexity. However, the SparseLocalOpt hierarchy does not contain every pseudo-Boolean function.

**Fig. 1:**
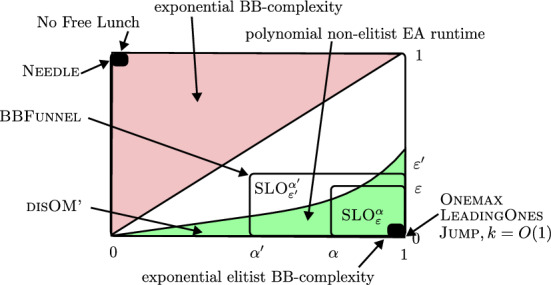
Illustration of results in this paper

Here, we introduce a refined problem classification which we call Slo$^\alpha _{\varepsilon ,r}$ with three parameters $\alpha ,\varepsilon \in [0,1]$ and $r\in [n]$. Similarly to the work by Dang et al, we consider the *sparsity* $\varepsilon$ of deceptive regions (“local optima”) and the *density* $\alpha$ of surrounding “fitness valleys” (see Definitions 4 and 5). Finally, the parameter *r* determines the range at which the constraints induced by the class needs to hold. We will see that the parameter *r* will play a limited role, and in most cases, it will suffice to assume that it is a small constant (e.g., as in Corollary 46). For any parameters $\alpha ,\varepsilon \in [0,1],r\in [n]$ and pseudo-Boolean function $f:\{0,1\}^n\rightarrow \mathbb {R}$, we say that *f* belongs to the class Slo$^\alpha _{\varepsilon ,r}$ if all its deceptive regions are $(\varepsilon ,r)$-sparse, and all its fitness valleys are $\alpha$-dense (Definition 8). The parameters induce a problem hierarchy (see Fig. 1): whenever $0\le \alpha '\le \alpha \le 1,$ $0\le \varepsilon \le \varepsilon '\le 1$, and $r\ge r'$, $\textsc{Slo}^{\alpha }_{\varepsilon ,r} \subseteq \textsc{Slo}^{\alpha '}_{\varepsilon ',r'}.$

Informally, increasing the parameter $\varepsilon$ relaxes the sparsity requirement for the deceptive regions, and decreasing the parameter $\alpha$ relaxes the density requirement on the fitness valleys. In the limit when $\alpha =0$ and $\varepsilon =1$ (“bad parameters”), the problem class contains all pseudo-Boolean functions (cf. Lemma 10). In the other limit, when $\alpha =1$ and $\varepsilon =0$ (“good parameters”), the class contains only completely non-deceptive problems, such as linear functions. We will show that all functions in the class $\textsc{Slo}^{\alpha }_{\varepsilon ,r}$ for moderately good parameters can be optimised efficiently (c.f., Theorem 45) while $\textsc{Slo}^{\alpha }_{\varepsilon ,r}$ for moderately bad parameters contains functions that cannot be optimised efficiently. Note that not all functions in $\textsc{Slo}^{\alpha }_{\varepsilon ,r}$ for bad parameters are hard to optimise. This is consistent with the worst-case perspective of computational complexity, i.e., not all instances of a NP-complete problem are difficult to solve.

We prove the following results for this new classification:

- We classify well-known theoretical benchmark functions in the hierarchy.
- We show that any class of functions *F* which satisfies the conditions of the No Free Lunch Theorem (i.e., closed under permutation) belongs to $\textsc{Slo}^{1/n}_{1,r}$ but not to $\textsc{Slo}^{2/n}_{1,r}$ for some $r=(1-o(1))n$.
- We show that for any constant $\delta \in (0,1)$, any constant $\alpha \in (0,1-\delta )$, any constant $\varepsilon \in (\alpha +\delta ,1)$, and $r=n/2$, Slo$^\alpha _{\varepsilon ,r}$ has exponential black-box complexity (in the sense of [20]). Informally, the function class is hard for all black-box optimisation algorithms when the “sparsity” $\varepsilon$ of local optima is larger than the “density” $\alpha$ of fitness valleys.
- We show that Slo$^\alpha _{\varepsilon ,r}$ has exponential *elitist* black box complexity (in the sense of [18]) for any constant levels of denseness $\alpha$ and sparsity $\varepsilon$, which we demonstrate on the BBFunnel problem sub-class. This negative result (Theorem 41) implies that a large set of elitist EAs, including those with one-point, bit-wise or heavy-tailed mutation, crossover etc. are inefficient on some problems with even mild degrees of deception. This result can be seen as a corollary to Theorem 8 from [13].
- We show that perturbing the function values of any function $f\in \textsc{Slo}^{\alpha }_{\varepsilon ,r}$ on a $\delta$-sparse set leads to a problem class belonging to $\textsc{Slo}^{\alpha -\delta }_{\varepsilon +\delta ,r}$. As an application, we locate a slight variation of the recently introduced disOM$_{d,p}$ function in the SLO hierarchy. As a corollary, we show that perturbing every search point with constant probability still leaves the problem in a class that can be solved to optimality in expected polynomial time with appropriately tuned non-elitist EAs. In contrast, previous analyses of $(1,\lambda )$ EA only considered perturbation probabilities up to $p=O(n^{-\delta })$ for constant $\delta$.
- Finally, we show that non-elitist EAs with bit-wise mutation have expected polynomial runtime on Slo$^\alpha _{\varepsilon ,r}$, given the appropriate values of $\alpha , \varepsilon , r$ and selection and mutation parameters (Theorem 45). In particular, this applies to EAs with 3-tournament selection and linear ranking selection [5]. Corollaries for non-elitist EAs for some classes of Knapsack and Vertex Cover problems illustrate applicability of this result.

A preliminary version of the paper appeared in [10], in which a small representative subset of the results are shown while the proofs and the rest of the results were only available to the reviewers of the conference. This is a follow-up work of the previous classification [13] in which the hierarchy was incomplete. In this journal paper, all results are presented with the complete proofs, and the general analyses of the linear and power-law ranking selections are new.

The remainder of the paper is organised as follows. Common notations in evolutionary computation and black-box complexity are provided in Sect. 2, then the SLO-Hierarchy is formalised and its properties are explored in Sect. 3. The follow-up section gives examples of functions and their stands in the hierarchy. Finally, the results for black-box algorithms and non-elitist algorithms are shown in Sects. 5, 6 and 7.

## Preliminaries

We introduce the notation that will be used throughout the paper.

### Basic Notation

The natural and base-2 logarithms are denoted by $\ln (\cdot )$, and $\log (\cdot )$ respectively. $\mathbb {N}$ is the set of natural numbers and $\mathbb {N}_0$ is the set of whole numbers. For any $n\in \mathbb {N}$, define $[n]:=\{1,\dots , n\}$, and similarly we use $[n]_0:=[n]\cup \{0\}$ for the inclusion of 0. By convention, the empty sum and product are 0 and 1 respectively, i.e., $\sum _{i\in \emptyset } c_i=0, \prod _{i\in \emptyset } c_i=1$ regardless of how $c_i$ are defined. The Iverson bracket is denoted by $[\cdot ]$.

The *Hamming distance* is denoted by $\mathrm{H}(\cdot ,\cdot )$. The *Hamming sphere* (or *shell*) with radius $r\in [n]$ around a bitstring $x\in \{0,1\}^n$ is defined by $S_r(x):= \left\{ y\in \{0,1\}^n\mid \mathrm{H}(x,y) = r \right\} .$ Clearly, $|S_r(x)|={n\atopwithdelims ()r}$. The power set of a finite set *A* is denoted $\mathcal {P}(A)$.

For any finite set *X*, any function $f: X\rightarrow Y$, and any permutation $\sigma : X\rightarrow X,$ let $\sigma f$ be the function defined by $(\sigma f)(x):=f(\sigma x)$. For any finite set *X*, a class of functions $F \subseteq \{ f \mid f: X\rightarrow Y\}$ is *closed under permutation* if and only if for all $f\in F$ and for any permutation $\sigma : X\rightarrow X$, $(\sigma f)\in F$.

Throughout the paper, we consider the maximisation of a function $f:X\rightarrow Y$, and *f* has a *unique global maximum* $x\in X$ if and only if $\forall y\in X, y\ne x\Longrightarrow f(x)>f(y)$. Furthermore, for any subsets $A,B\subseteq X$, we define $f(A)\ge f(B)$ to denote that for all $x \in A$ and for all $y\in B$, $f(x)\ge f(y)$.

Given a partition of a search space $\mathcal {X}$ into *m* ordered “levels” $(A_1,\dots ,A_m)$, we define $A_{\ge j}:=\cup _{i=j}^{m} A_i$.

Given two bitstrings *x* and *y*, we let $x\cdot y$ and *xy* denote the concatenated bitstrings.

Some definitions use the benchmark pseudo-Boolean functions $\textsc{ OneMax} (x) := \textsc{Om} (x) := \sum _{i=1}^n x_i$, and $\textsc{Lo} _y(x) := \sum _{i=1}^n \prod _{j=1}^i [x_j=y_j]$ for a target bitstring *y*. In particular, *y* is the all-ones string for the standard LeadingOnes function.

For an event $\mathcal {E}$ and random variable *X*, we use the standard notation $E\left[ X;\mathcal {E}\right] :=E\left[ X1_{\mathcal {E}}\right]$ where $1_\mathcal {E}$ is the indicator random variable $1_\mathcal {E}(\omega ):= [\omega \in \mathcal {E}]$ (see e.g., Section 6.3 in [41]).

A *population* of $\lambda \in \mathbb {N}$ points of the search space $\mathcal {X}$ is a vector $P\in \mathcal {X}^\lambda$, the *i*-th individual of *P* is denoted *P*(*i*). Given $x \in \mathcal {X}$, define $\mathrm{H}(x, P):= \min _{j \in [|P|]}\{\mathrm{H}(P(j),x)\}$, and for $A \subseteq \mathcal {X}$, we let $|P \cap A|:= |\{i \mid P(i) \in A\}|$, i.e., the number of individuals of *P* belonging to *A*.

**Algorithm 1:**
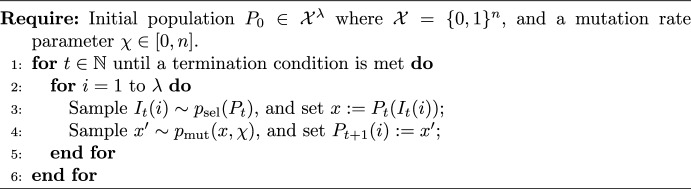
Non-elitist EA with unary variation operator [8].

**Algorithm 2:**
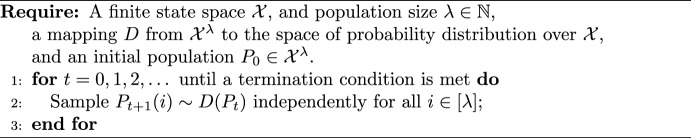
Population-based algorithm [5].

All non-elitist EAs with *unary variation operators* can be cast in the framework of Algorithm 1 [8]. A new population $P_{t+1}$ is generated by independently sampling $\lambda$ individuals from an existing population $P_t$ according to a selection mechanism $p_{\mathrm{sel}}$, then by perturbing each of the selected individuals with a unary variation operator $p_{\mathrm{mut}}$. The algorithm in turn is a special case of a more general framework of Algorithm 2, for which the *level-based theorem* [5] was developed. To prove the positive results for our paper, we derive a variant of that theorem, c.f. Theorem 42.

We will characterise selection mechanisms in the following way.

#### Definition 1

If the individuals in a population *P* are ordered by decreasing fitness i.e., $f(P(1))\ge f(P(2)) \ge \cdots \ge f(P(\lambda ))$, then for any $0 \le \psi \le \gamma \le 1,$ the value $\beta (\psi ,\gamma ,P)$ is the probability that the selection mechanism chooses an individual ranked between $\lceil \psi \lambda \rceil$ and $\lceil \gamma \lambda \rceil$ in the population.

We omit the symbol $P$ from the notation when the population is clear from the context. Note also that the 2-argument variant of the definition of $\beta$, such as the one used in Lehre [30], is the special case of the above notation with $\psi =0$.

A popular selection mechanism is *k*-*tournament*. For this mechanism, $p_{\mathrm{sel}}$ returns ${\mathrm{argmax}}_{i \in S} f(P_t(i))$ where *S* is a multi-set of *k* random numbers drawn independently and uniformly from $[\lambda ]$. The corresponding $\beta$ is non-linear, with $\beta (\gamma _1,\gamma _2)=(1-\gamma _1)^k-(1-\gamma _2)^k$.

The tournament selection satisfies the following natural property, the so-called *f*-*monotonicity*: if *x*, *y* are two individuals of the population with $f(x)\ge f(y)$, then the probability of selecting *x* is at least that of selecting *y*. It is easy to see that for an *f*-monotone selection mechanism, the selection probability satisfies the inequality $\beta (\psi _1,\psi _1+\gamma ) \ge \beta (\psi _2,\psi _2+\gamma )$ for any $\psi _1 \le \psi _2$ and any $\gamma >0$.

We denote the standard *bitwise mutation operator* by $p_{\mathrm{mut}}$ and it is configured by a parameter $\chi \in (0,n/2]$ so that for any pair of bitstrings $x,x'\in \{0,1\}^n$, the probability of obtaining $x'$ from *x* is $\Pr \left( x'=p_{\mathrm{mut}} (x, \chi )\right) = \left( \chi /n\right) ^{\mathrm{H}(x,x')}\left( 1-\chi /n\right) ^{n-\mathrm{H}(x,x')}.$ We also use $p_{\mathrm{mut}} (x)$ to denote the distribution of the outcome string when applying the mutation operator to a string *x*. Thus $y\sim p_{\mathrm{mut}} (x)$ means *y* is sampled from that distribution, or in other words, *y* is the outcome of applying the operator to *x*.

### Black-Box Complexity

Evolutionary algorithms belong to the class of so-called *black-box algorithms* that query function values to optimise functions. Such a query is referred to as fitness evaluation. Let $f:\{0,1\}^n\rightarrow \mathbb {R}$ be a pseudo-Boolean function and *A* be a black-box algorithm. We use *T*(*f*, *A*) to denote the number of fitness evaluations, i.e., the *running time*, until *A* queries an optimal search point $x^* \in {\mathrm{argmax}}\, f$ for the first time. If *A* can make random decisions (so-called *randomised algorithms*), which is the case for EAs, then *T*(*f*, *A*) is a random variable and we are mostly interested in $E\left[ T(f,A)\right]$. For a class $\mathcal {F}$ of pseudo-Boolean functions, the complexity of *A* on $\mathcal {F}$ is:

$$\begin{aligned} \textsc{C} (\mathcal {F},A) := \sup _{f\in \mathcal {F}} E\left[ T(f,A)\right] , \end{aligned}$$

that is, the worst-case running time of *A* on $\mathcal {F}$. For a class $\mathcal {A}$ of black-box algorithms, the black-box complexity of $\mathcal {F}$ by $\mathcal {A}$ is:

$$\begin{aligned} \textsc{BBC} (\mathcal {F},\mathcal {A}) := \inf _{A\in \mathcal {A}} \textsc{C} (\mathcal {F},A), \end{aligned}$$

that is, the complexity of the best algorithm in $\mathcal {A}$. The class of unrestricted black-box algorithms is the largest class of black-box algorithms as it puts no restriction on what the algorithms can do outside of the fitness evaluations.

A general tool to prove lower bounds for black-box complexities is the so-called Yao’s Minimax Principle [45].

#### Theorem 2

(Yao’s Minimax Principle [45]). Let $\Pi$ be a problem with a finite set $\mathcal {I}$ of input instances of fixed size permitting a finite set $\mathcal {A}$ of deterministic algorithms. Let $I_p$ be a randomly chosen instance with a probability distribution *p* over $\mathcal {I}$ and let $A_q$ be a randomly chosen algorithm with a probability distribution *q* over $\mathcal {A}$. Then for all *p* and all *q*, it holds

$$\begin{aligned}\min _{A\in \mathcal {A}}E\left[ T(I_p,A)\right] \le \max _{I\in \mathcal {I}}E\left[ T(I,A_q)\right] ,\end{aligned}$$

where *T*(*I*, *A*) denotes the running time of $A\in \mathcal {A}$ on $I \in \mathcal {I}$.

The principle uses the standard convention that any randomised algorithm can be viewed as a probability distribution *q* over a set $\mathcal {A}$ of deterministic algorithms, thus the algorithm is denoted $A_q$. It says that the performance of the best deterministic algorithms on a random input, chosen according any fixed distribution *p* over a class $\mathcal {I}$, is a lower bound for the performance of any randomised algorithm $A_q$ on $\mathcal {I}$. Here, $\mathcal {I}$ coincides with our notion of function class $\mathcal {F}$. However, to apply Yao’s minimax principle, both the sets $\mathcal {A}$ and $\mathcal {I}$ or $\mathcal {F}$ must be finite. The finiteness of $\mathcal {F}$ is easy to handle because it suffices to consider a finite subset of functions over the infinite one.

#### Lemma 3

(Lemma 4.3 in Jansen [27]). Let $\mathcal {F}, \mathcal {G}$ be classes of pseudo-Boolean functions and $\mathcal {A}$ be a class of algorithms. Then

$$\begin{aligned} \mathcal {F}\subseteq \mathcal {G} \Rightarrow \textsc{BBC} (\mathcal {F},\mathcal {A}) \le \textsc{BBC} (\mathcal {G},\mathcal {A}). \end{aligned}$$

## The SLO-Hierarchy

We introduce a class of fitness landscapes Slo$^\alpha _{\varepsilon ,r}$, which we claim, separates elitist evolutionary algorithms from non-elitist ones. The class contains all functions which satisfy the following requirements. We first require that from any point in the search space, there must exist a not too long directed path to the global optimum, where consecutive steps on the path are near each other in the search space. Along any path, we distinguish between “deceptive regions” and “fitness valleys”. Any region of the path with higher fitness than a later part of the path is called deceptive. Conversely, any region with lower fitness than an earlier part of the path is called a fitness valley. We only impose the constraint that deceptive regions must be sparse, while fitness valleys must be dense. Informally, a set is called dense if every member of the set has many neighbours in that set or the set of better search points. A set is called sparse if for any search point *x* and any radius *r*, sampling another search point uniformly at random among search points at distance *r* from *x* is unlikely to belong to the set. We first give the formal definition of Slo$^\alpha _{\varepsilon ,r}$ and show that any pseudo-Boolean function belongs to at least one of these classes.

### The Definition of Slo$^\alpha _{\varepsilon ,r}$

#### Definition 4

For $\alpha \in [0,1],$ a subset $C\subseteq \{0,1\}^n$ is called $\alpha$-*dense* with respect to a set *D* if for all ${x\in C}$, $\left| S_1(x)\cap D\right| \ge \alpha n.$

The term density has been employed with different meanings in mathematics and computer science. This paper usually considers density of a set of search points *C* within which a sizeable portion of the current population resides, with respect to a superset $D=C\cup E$ where the elements of *E* are equally good or better neighbours of *C* (where “better” is context-dependent). With this interpretation, stating that *C* is $\alpha$-dense with respect to *D* for a large $\alpha$ implies that the neighbourhoods of the search points in *C* are “dense” with good solutions. Note that the previous definition of density from [13] uses a more restricted case where *C* and *D* are identical.

#### Definition 5

For $n\in \mathbb {N},\varepsilon \in [0,1]$ and $r_{\max }\in [n-1]$, a subset $B\subseteq \{0,1\}^n$ is called $(\varepsilon ,r_{\max })$-*sparse* if for all $x\in \{0,1\}^n$ and for all $r\in [r_{\max }]$,

1 $$\begin{aligned} \left| S_r(x)\cap B\right| \le \varepsilon \cdot {n\atopwithdelims ()r}. \end{aligned}$$

The definition of sparsity is not only the opposite of density but the required condition must also hold for all search points *x* of the search space and for Hamming spheres of radii up to $r_{\max }$. In other words, the opportunities of entering *B* from the outside or moving between elements of *B* are limited and depend on the parameter $\varepsilon$. The previous definition of sparsity from [11, 13] uses different conditions depending on whether $x\in B$ or not, then chooses $r_{\max }=n-1$. Here we use the same condition and add the flexibility to choose $r_{\max }$. We will see later that the most interesting cases often require a mild condition on $r_{\max }$, i.e., $r_{\max }=\omega (1)$. As already noticed in [11], we eliminate the possibility of having $r_{\max }=n$ to avoid the non-exciting case that for all $\varepsilon <1$ the only $(\varepsilon ,n)$-sparse set is the empty set. This is because if *B* is not empty then by taking any element $x\in B$ we see that (1) is not satisfied for the complement point ${\bar{x}}$ of *x*, i.e., $|S_n({\bar{x}})\cap B|=|\{x\}|=1>\varepsilon {n \atopwithdelims ()n}$. Finally, note that if *B* is $(\varepsilon ,r_{\max })$-sparse for $r_{\max }\ge \lfloor \frac{n}{2}\rfloor$, the following lemma implies that *B* must also be $(\varepsilon ,n-1)$-sparse because inequality (1) must hold simultaneously for any *x* and the complement bitstring ${\bar{x}}$.

#### Lemma 6

If *B* is $(\varepsilon ,\lfloor \frac{n}{2}\rfloor )$-sparse, then *B* is also $(\varepsilon ,n-1)$-sparse.

#### Proof

Choose any *x* and $r\in [n-1]$. If $r\le \lfloor \frac{n}{2}\rfloor$, then (1) holds by the assumption that *B* is $(\varepsilon ,\lfloor \frac{n}{2}\rfloor )$-sparse. It therefore remains to consider the case where $\lfloor \frac{n}{2}\rfloor <r\le n-1.$ We intend to prove this case by applying (1) to the complement bitstring ${\bar{x}}$. For all *y*, we have $H(x,y)=n-H({\bar{x}},y)$ hence $S_r(x)=S_{n-r}({\bar{x}})$. It is easy to see that $n-r\le \lfloor \frac{n}{2}\rfloor$. If *n* is odd, then $n=2m+1$ for some integer *m*, and

$$\begin{aligned}n-r< n-\left\lfloor \frac{n}{2}\right\rfloor =n-m = m+1 = \left\lfloor \frac{n}{2}\right\rfloor +1.\end{aligned}$$

On the other hand, if *n* is even, then

$$\begin{aligned} n-r< n-\left\lfloor \frac{n}{2}\right\rfloor =n-\frac{n}{2} = \frac{n}{2} = \left\lfloor \frac{n}{2}\right\rfloor . \end{aligned}$$

We now have

2 $$\begin{aligned} |S_r(x)\cap B| = |S_{n-r}({\bar{x}})\cap B| \le \varepsilon \cdot {n\atopwithdelims ()n-r} = \varepsilon \cdot {n\atopwithdelims ()r}, \end{aligned}$$

where the inequality follows from $(\varepsilon ,\lfloor \frac{n}{2}\rfloor )$-sparsity of *B* and $n-r\le \lfloor \frac{n}{2}\rfloor$. We have therefore proved (1) for all $r\in [n-1]$ and the lemma follows. $\square$

To make the notion of deceptive regions and fitness valleys more precise, we formally define deceptive pairs.

#### Definition 7

Given a function $f:\{0,1\}^n\rightarrow \mathbb {R}$ and a partition $(A_1,\ldots ,A_m)$ of $\{0,1\}^n$, a pair $(A_i,A_j)$ is called *f*-deceptive if $1\le i<j\le m$ and there are elements $x\in A_i,$ $y\in A_j$ such that $f(x)\ge f(y)$.

The deceptive pair notion means that the fitness of search points in the lower level $A_i$, with respect to the order defined by the partition, can be as good as or even exceed the fitness of search points in the higher level $A_j$. In what follows, the first element of a deceptive pair will be called a *deceptive element* and the second element will be called a *valley element*. We now state the definition of the problem class.

#### Definition 8

An objective function $f:\{0,1\}^n\rightarrow \mathbb {R}$ belongs to the problem class Slo$^\alpha _{\varepsilon ,r}$ if there exists a partition of $\{0,1\}^n$ into $m\in {\mathrm{poly}}(n),$ subsets $(A_1,\ldots , A_m)$ such that

1. $$A_m = \{x\in \{0,1\}^n\mid \forall y\in \{0,1\}^n, f(x)\ge f(y)\}$$,
2. $$\forall j\in [m-1],\forall {x\in A_j},\exists {y\in A_{\ge j+1}}$$ s.t. $$\mathrm{H}(x,y)=O(1),$$
  and if $(A_{i_1},A_{j_1}),\ldots , (A_{i_u},A_{j_u})$ are *f*-deceptive pairs then
3. $\cup _{v=1}^u A_{i_v}$ is $(\varepsilon ,r)$-sparse, and
4. $A_{j_{v}}$ is $\alpha$-dense with respect to $A_{\ge j_v}$ for all $v\in [u]$.

The two first conditions are standard for level-based methods [5] that the last level $A_m$ contains all the global optima and that it is not hard to progress throughout the levels using the bitwise mutation. The two last conditions are only required when there are deceptive pairs in the partition, namely the union of the deceptive levels $\cup _{v=1}^u A_{i_v}$ must be sparse, while each valley level must be dense. The following lemma is derived immediately from Definitions 4, 5 and 8.

#### Lemma 9

For all $\alpha '\le \alpha$, $\varepsilon \le \varepsilon ',$ and $r'\le r$, it holds

3 $$\begin{aligned} \textsc{Slo}^{\alpha }_{\varepsilon ,r} \subseteq \textsc{Slo}^{\alpha '}_{\varepsilon ',r'}. \end{aligned}$$

We first prove a simple lemma stating that for any $r\ge 1$ and $\alpha =O(1/n)$, the class Slo$^{\alpha }_{1,r}$ contains all pseudo-Boolean functions.

#### Lemma 10

For any constant $c\ge 1$, any $r\ge 1$, and any pseudo-Boolean function $f:\{0,1\}^n\rightarrow \mathbb {R}$, $f\in \textsc{Slo}^{c/n}_{1,r}$.

#### Proof

We prove the lemma by constructing a partition with $m\le n$ levels and then proving that this partition satisfies the conditions of Definition 8 for $\alpha =c/n$, $\varepsilon =1$ and any *r*.

Define the final level $A_m = \{x\mid \forall y, f(x)\ge f(y)\}$ and pick an arbitrary element $x^*\in A_m$. Define $A_{m-1}:=\{x\mid 1\le H(x,x^*)\le c\}{\setminus} A_m$, and $A_i:=\{x\mid H(x,x^*)=m-i+c-1 \}{\setminus} A_m$ for $i\in [1..m-2]$.

Condition 1 is satisfied by construction of $A_m$. Condition 2 is also satisfied because for all $i<m,$ and all $x\in A_i,$ the Hamming distance to the next level is $H(x,A_{i+1})\le c$. Condition 3 is immediately satisfied because $\varepsilon =1$. Finally, to see that condition 4 is satisfied, we distinguish between three cases. In the case where $x\in A_j$ for $j\le m-2$, the element *x* must have at least *c* Hamming-neighbours in $A_{j+1}$ or $A_m$, i.e.,

$$\begin{aligned} |S_1(x)\cap A_{\ge j}| \ge |S_1(x)\cap (A_{j+1}\cup A_m)| \ge c = \alpha n. \end{aligned}$$

Secondly, in the case where $x\in A_{m-1}$ and $H(x,x^*)=c$, then *x* has at least *c* Hamming-neighbours in $A_{m-1}$ or $A_m$, i.e.,,

$$\begin{aligned} |S_1(x)\cap A_{\ge m-1}| = |S_1(x)\cap (A_{m-1}\cup A_m)| \ge c \ge \alpha n. \end{aligned}$$

Thirdly, in the case where $x\in A_{m-1}$ and $H(x,x^*)=d$ for $1\le d<c$, then *x* has at least $n-d>n-c$ Hamming-neighbours in $A_{m-1}$ or $A_m$, i.e.,,

$$\begin{aligned} |S_1(x)\cap A_{\ge m-1}| \ge |S_1(x)\cap (A_{m-1}\cup A_m)| \ge n-c \ge \alpha n. \end{aligned}$$

In all cases, condition 4 is satisfied and the lemma follows. $\square$

Furthermore, the hierarchy is populated by functions for all values of $\alpha$ and $\varepsilon$. In particular, the construction of the so-called BBFunnel problem [13] (see Definition 31) illustrates that for any constants $\alpha$ and $\varepsilon =\Omega (\log n/n)$, there exists an instance of BBFunnel in Slo$^\alpha _{\varepsilon ,r}$ which does not belong to Slo$^{\alpha (1+\delta )}_{\varepsilon (1-\delta ),r}$.

Regarding the number of levels that a partition satisfying Definition 8 can have, we have the following property. If *f* has *N* global optima where $N<c2^n$ for some constant $c\in (0,1)$ then the number of levels in any level partition satisfying Definition 8 is $m=\Omega (n-\sqrt{n\log (N)})$.

#### Lemma 11

For any partition $A:=(A_1,\ldots ,A_m)$ with $N:=|A_m|<c2^n$ for some constant $c\in (0,1)$ satisfying condition (2) in Definition 8, it holds that $m=\Omega (n-\sqrt{n\log (N)})$.

#### Proof

We assume that $cm<n/2$, because otherwise $m=\Omega (n)$ and the result follows immediately.

We apply the incompressibility method [32] (see Appendix C), where $C(x\mid y)$ is the Kolmogorov complexity of *x* conditional on *y*. Consider any level-partition $A:=(A_1,\ldots , A_m)$ for *f*, and let $N:=|A_m|$. The $2^n-N$ non-optimal search points are partitioned into $m-1$ levels.

We will compute an upper bound on $C(x_j\mid A)$ by providing an encoding of an arbitrary element $x_j$ of $A_j$ given a description of the partition *A*. By condition 2 of Definition 8, there must exist a sequence of search points $y_1,y_{2},\ldots ,y_\ell$, where $y_1=x_j$, and a strictly increasing function $\sigma :[\ell ]\rightarrow [j..m]$ such that $\sigma (1)=j, \sigma (\ell )=m$, and $y_i\in A_{\sigma (i)}$ for all $i\in [\ell ]$, and where for all $i\in [\ell -1]$ we have $H(y_i,y_{i+1})\le d$ for some constant *d*. Hence, by the triangle inequality, the Hamming distance between $y_1=x_j$ and $y_m$ is at most $d\ell \le dm$. Thus, we can obtain $x_j$ from an encoding of $y_{\ell }\in A_m$ using at most $\log {n\atopwithdelims ()dm}+c_1$ bits (i.e., encode the set of bit-positions where they differ) for some constant $c_1$. Furthermore, from $A_m$, the optimal search point $y_\ell$ can be obtained using $\log (N)+c_2$ bits (i.e., encode $y_\ell$ by the encoding the index of $y_\ell$ in an enumeration of $A_m$). It follows that the Kolmogorov complexity of $x_j$ conditional on *A* is at most

4 $$\begin{aligned} C(x_j\mid A) \le \log (N)+\log {n\atopwithdelims ()dm}+c_1+c_2+c_3 \end{aligned}$$

bits for some constant $c_3\ge 1$.

However, by Theorem 58 (incompressibility lemma), there must exist at least one element $x_j\in \{0,1\}^n{\setminus} A_m$ with conditional Kolmogorov complexity

5 $$\begin{aligned} C(x_j\mid A) \ge \log (2^n-N)-c_4 \end{aligned}$$

for some constant $c_4\ge 1$. By combining (4) and (5), we obtain for $c_5=e^{c_1+c_2+c+3+c_4}>1$

$$\begin{aligned} \log (2^n/N-1)&\le \log {n\atopwithdelims ()dm} + \log (c_5).\\ \end{aligned}$$

Hence, exponentiating both sides and applying inequality (1.4.19) from [17] give

$$\begin{aligned} \frac{2^n}{c_5N}-\frac{1}{c_5}&\le {n\atopwithdelims ()dm} \le 2^n\exp \left( -\frac{2(dm-n/2)^2}{n}\right) . \end{aligned}$$

Solving for *m*

$$\begin{aligned} \frac{1}{c_5N}-\frac{1}{2^{n}c_5}&\le \exp \left( -\frac{2(dm-n/2)^2}{n}\right) , \end{aligned}$$

which implies

$$\begin{aligned} \frac{2(dm-n/2)^2}{n}&\le \ln \left( \frac{c_5N}{1-N/2^n}\right) \end{aligned}$$

and

$$\begin{aligned} dm&\ge \frac{n}{2}-\sqrt{\frac{n}{2}\ln \left( \frac{c_5N}{1-N/2^n}\right) } \end{aligned}$$

which implies the main result since $N<c'2^n$ for some constant $c'\in (0,1)$. $\square$

### Properties of the SLO Class

The following lemma gives some basic properties of the sparsity definition and showcases the sparsity relationship between supersets.

#### Lemma 12

The following properties hold for the sparsity definition.

1. The empty set $\emptyset$ is $(0,n-1)$-sparse.
2. For any $z \in \{0,1\}^n$, $\{z\}$ is $(1/n,n-1)$-sparse.
3. If $B\subseteq \{0,1\}^n$ is $(\varepsilon ,r)$-sparse, then any $B'\subseteq B$ is also $(\varepsilon ,r)$-sparse.
4. Let $A_1,A_2,\ldots ,A_m\subseteq \{0,1\}^n$ where $A_i$ is $(\varepsilon _i,r_{i})$-sparse for $i \in [m]$, it holds that $\cup _{i=1}^m A_i$ is $\left( \sum _{i=1}^m\varepsilon _i,\min _{i\in [m]}\left\{ r_{i}\right\} \right)$-sparse.

#### Proof

We show each property separately.

1. For any $x\in \{0,1\}^n$ and all $r\in [n-1]$, $|S_r(x)\cap \emptyset | = 0 = 0\cdot {n\atopwithdelims ()r}.$
2. For any $x\in \{0,1\}^n$ and all $r\in [n-1]$, it holds
  $$\begin{aligned} |S_r(x)\cap \{z\}| \le |\{z\}| = 1 = \frac{1}{{n \atopwithdelims ()r}}{n \atopwithdelims ()r} \le \frac{1}{{n \atopwithdelims ()1}}{n \atopwithdelims ()r}, \end{aligned}$$
  thus $\{z\}$ is $(1/n,n-1)$-sparse by Definition 5.
3. The inclusion $B'\subseteq B$ and the sparsity of *B* imply for all $r'\in [r]$
  $$\begin{aligned} |S_{r'}(x)\cap B'| \le |S_{r'}(x)\cap B| \le \varepsilon {n\atopwithdelims ()r'}, \end{aligned}$$
  hence $B'$ is $(\varepsilon ,r)$-sparse.
4. Let $A:=\cup _{i=1}^m A_i$, $\varepsilon :=\sum _{i=1}^m\varepsilon _i$ and $r:=\min _{i\in [m]} \{r_{i}$}, then for any $r'\in [r]$ and $x\in \{0,1\}^n$, we have
  $$\begin{aligned} |S_{r'}(x)\cap A|&\le \sum _{i=1}^m|S_{r'}(x)\cap A_i| \le \sum _{i=1}^m\varepsilon _i{n\atopwithdelims ()r'} = \varepsilon {n\atopwithdelims ()r'}. \end{aligned}$$
  Therefore the set *A* is $(\varepsilon ,r)$-sparse by Definition 5.

$$\square $$

The density of a set after excluding some elements can be estimated as follows.

#### Lemma 13

If *C* is $\alpha$-dense with respect to *D*, and *B* is $(\varepsilon ,r)$-sparse, then $C{\setminus} B$ is $(\alpha -\varepsilon )$-dense with respect to $D{\setminus} B$.

#### Proof

Consider any element $x\in C{\setminus} B$. The $\alpha$-density of *C* with respect to *D* and the $(\varepsilon ,r)$-sparsity of *B* imply

$$\begin{aligned} |S_1(x)\cap D{\setminus} B|&\ge |S_1(x)\cap D| -|S_1(x)\cap B| \ge \alpha n - \varepsilon {n\atopwithdelims ()1} = (\alpha - \varepsilon ) n. \end{aligned}$$

$$\square $$

We need the following technical lemma for the next result.

#### Lemma 14

For any $\ell \in \mathbb {N}_0$ and any $n,k\in \mathbb {N}$ with $1\le k\le n$, it holds

$$\begin{aligned} {n+\ell \atopwithdelims ()k}\ge \left( 1+\frac{\ell }{n}\right) {n\atopwithdelims ()k}. \end{aligned}$$

#### Proof

The case $k=1$ is trivial as the equality is obtained:

$$\begin{aligned} {n+\ell \atopwithdelims ()1} = n+\ell = \left( 1 + \frac{\ell }{n} \right) {n \atopwithdelims ()1}. \end{aligned}$$

For $k\ge 2$, we prove the result by induction on $\ell$. The base case $\ell =0$ is trivial. Now assume the statement holds for some $i\ge 0$, then for $\ell =i+1$ we get

$$\begin{aligned} {n+\ell \atopwithdelims ()k}&= \left( \frac{n+\ell }{n+\ell +1-k}\right) {n+\ell -1\atopwithdelims ()k}, \end{aligned}$$

and using the induction hypothesis gives

$$\begin{aligned}&\ge \left( \frac{n+\ell }{n+\ell +1-k}\right) \left( \frac{n+\ell -1}{n}\right) {n\atopwithdelims ()k}\\&= \left( \frac{n+\ell }{n}\right) \left( \frac{n+\ell -1}{n+\ell +1-k}\right) {n\atopwithdelims ()k} \overset{(k\ge 2)}{\ge }\ \left( \frac{n+\ell }{n}\right) {n\atopwithdelims ()k}. \end{aligned}$$

The statement now follows for all $\ell \in \mathbb {N}$ by induction. $\square$

The following lemma shows that the set of bitstrings that agree with a fixed bitstring *z* on a set of $(1-\varepsilon )n$ bit positions is $\varepsilon$-sparse. Such sets correspond to the notion of *schemata* in early evolutionary computation theory.

#### Lemma 15

Assume $z\in \{0,1\}^n$ and $I\subseteq [n]$ with $|I|\ge (1-\varepsilon )n$ for some $\varepsilon \in \{0,1/n,\ldots ,1-1/n,1\}.$ Let $B := \left\{ x \mid x_i = z_i \ \forall i\in I\right\}$, i.e., the set of all bit strings that agree with *z* at the positions defined by *I*, then *B* is $(\max (1/n,\varepsilon ),n-1)$-sparse.

#### Proof

By Lemma 6, it suffices to prove the statement for the parameter $r_{\max }:=\lfloor n/2\rfloor$.

It suffices to prove the lemma for all *I* such that $|I| = (1 - \varepsilon )n$, since for any $I, I'$ such that $|I'| > (1 - \varepsilon )n$ and $I \subset I'$ and for sets $B_I = \{x \mid x_i=z_i \forall i \in I\}$ and $B_{I'} = \{x \mid x_i=z_i \forall i \in I'\}$ we have $B_{I'} \subset B_I$ (since for *x* to agree with *z* in positions in $I'$ it is necessary to do so in all positions in *I*). Hence if $B_I$ is $(\varepsilon ', r')$-sparse for some $\varepsilon '$ and $r'$, then by Lemma 12(3), so is $B_{I'}$.

In the case $\varepsilon =0$, we have $|B|=1$ and the statement follows directly from Lemma 12(2). In the case $\varepsilon =1$, we trivially satisfy Definition 5. Thus in the remaining of the proof, we assume $1/n\le \varepsilon \le 1-1/n$.

We assume without loss of generality that $z=1^n$ and $I=[(1-\varepsilon )n]$. For any bit string, we call the “prefix” the first $(1-\varepsilon )n$ bit-positions, and the “suffix” the last $\varepsilon n$ bit-positions. For any fixed $x\in \{0,1\}^n$, let $a:=\sum _{i=1}^{(1-\varepsilon )n} (1-x_i)$ be the number of 0s of in the prefix of *x*. Note that all strings $y\in B$ must have the prefix being all 1s, therefore for all $r< a$, we already have $H(x,y)\ge a > r$ so $S_r(x)\cap B=\emptyset$ and

$$\begin{aligned} |S_r(x)\cap B| = 0 < \varepsilon {n\atopwithdelims ()r} \end{aligned}$$

trivially holds for all $\varepsilon \ge 1/n$.

In the remaining cases where $r \in [\lfloor n/2\rfloor ] \cap [a,\infty )$, let $y\in S_r(x)\cap B$. This means $H(x,y)=r$ and *y* has a fixed prefix of all 1s. Since the prefixes of *x* and *y* already contribute *a* to the Hamming distance, then *y* and *x* must differ by exactly $r-a$ bits in the suffix. The number of ways that strings *y* can be chosen, using ${n\atopwithdelims ()i}\le {n\atopwithdelims ()r}$ which holds for all $i\le r\le n/2$, is

$$\begin{aligned} |S_r(x)\cap B|&= {\varepsilon n\atopwithdelims ()r-a} \le {\varepsilon n\atopwithdelims ()r}, \end{aligned}$$

then applying Lemma 14 with $\ell :=(1-\varepsilon )n$, $k:=r$ and $\varepsilon n$ in place of *n* gives

6 $$\begin{aligned}&\le \left( 1 + \frac{(1-\varepsilon )n}{\varepsilon n} \right) ^{-1} {\varepsilon n + (1-\varepsilon )n \atopwithdelims ()r} = \varepsilon {n \atopwithdelims ()r}. \end{aligned}$$

The conditions of Definition 5 are satisfied in all cases, thus the lemma follows. $\square$

## Examples of SLO Function Classes

We give a list of benchmark functions and well-known problems and how they can be classified in the hierarchy of Slo$^\alpha _{\varepsilon ,r}$. For any $r\ge 1$, some easy problems that can be solved by simple evolutionary algorithms, e.g., by the (1+1) EA, in expected polynomial time are in Slo$^{1}_{0,r}$. On the other extreme, deceptive or unstructured problems are in Slo$^{c/n}_{1,r}$. This includes classes of functions that are closed under permutation, where the No-Free Lunch (NFL) theorem [19] tells us that on average no algorithm can outperform unstructured random search. In between there are interesting problems, such as instances of NP-hard combinatorial problems. The instances that we will use to illustrate this are well understood and can be solved in polynomial time. In particular, some have been used to in the literature to show the limitations of greedy algorithms [4]. We also consider functions under random perturbation, and show how their hierarchy is changed with respect to the parameters of the perturbation. Table 1 summarises our classification and Fig. 1 gives an overview of structure of the hierarchy.

**Table 1:** Density and sparsity bounds for some functions, here $c\ge 1$ is any constant. All results hold for the parameter $r=n-1$. Result $[^a]$ holds with probability $1-o(1)$, result $[^b]$ holds with a constant probability

Problem class	$\boldsymbol{\alpha }$	$\boldsymbol{\varepsilon }$	Reference
Linear functions	1	0	Theorem 16
LeadingOnes	1	0	Theorem 16
Jump$_{k,n}$, $k\in O(1)$	1	0	Theorem 16
Plateau $_{k,n}$, $k\in O(1)$	1	0	Theorem 16
$\mathcal{G}(\textsc{OneMax},(1-\alpha )n,B)$ $\,\mathrm{with}\,\alpha >1/n, B\sim \mathcal {V}(n,p),$ $\,p<\left( \frac{\delta }{2e}\right) ^{1+\frac{1+\alpha }{\delta }}, \delta =\Omega (1)$	$\alpha -\delta$	$\delta$	Corollary 28$[^a]$
$\textsc{BBFunnel}$ $\,\mathrm{with}\,w\in \Theta (n), b\in {\mathrm{poly}}(n)$	$1-\frac{w}{n}$	$\omega \left( \frac{\log {n}}{n}\right)$	Theorem 39$[^b]$
$\textsc{Knapsack} _{u,v}$ $\,\mathrm{with}\,u,v \in (0,1)$	$u(1-v)+v$	*v*	Proposition 21
$\textsc{MISP}^{u,v}(K_{1,n})$ $\,\mathrm{with}\,u,v \in (0,1), v\le u$	*v*	$\frac{1}{n+1}$	Proposition 22
$\textsc{VCP} ^{u,v}(K_{1,n})$ $\,\mathrm{with}\,u,v \in (0,1), v\le u$	*v*	$\frac{1}{n+1}$	Proposition 23
Needle	$\left[ \frac{c}{n},\infty \right) {\setminus} \omega \left( \frac{1}{n}\right)$	1	Corollary 18
Jump$_{k,n}$, $k\in \omega (1)$	$\left[ \frac{c}{n},\frac{k}{n}\right)$	1	Corollary 18
Closed under permutation	$\left[ \frac{c}{n},\infty \right) {\setminus} \omega \left( \frac{1}{n}\right)$	1	Theorem 20
All pseudo-Boolean functions	$\frac{c}{n}$	1	Lemma 10

### Theoretical Benchmark Functions

With every pseudo-Boolean function $f:\{0,1\}^n\rightarrow \mathbb {R}$, for a given *r*, we can associate an $\alpha$- and $\varepsilon$-value by finding the largest $\alpha$ and the smallest $\varepsilon$ such that $f\in \textsc{Slo}^\alpha _{\varepsilon ,r}$, i.e., corresponding to the smallest problem-class to which it belongs. Consider functions:

$$\begin{aligned} {\textsc{Jump}}_{k,n} (x)&:= {\left\{ \begin{array}{ll} k + \sum _{i=1}^n x_i & {\mathrm{if}}\quad \sum _{i=1}^n x_i\in [0\cdots n-k]\cup \{n\},\\ n-\sum _{i=1}^n x_i& {\mathrm{otherwise}}. \end{array}\right. } \end{aligned}$$

$$\begin{aligned}{\hspace{5ex}} {\textsc{Plateau}}_{k,n} (x)&:= {\left\{ \begin{array}{ll} n-k & {\mathrm{if}}\quad n-k\le \sum _{i=1}^n x_i< n,\\ \sum _{i=1}^n x_i & {\mathrm{otherwise}}. \end{array}\right. } {\hspace{5ex}}\end{aligned}$$

Table 1 gives all the results about the SLO hierarchy we have in the paper and the other functions (or problems) will be defined later on. The first four rows of that table are covered by the following theorem.

#### Theorem 16

LeadingOnes, Jump$_{k,n}$ and Plateau $_{k,n}$ with $k\in O(1)$, and any linear pseudo-Boolean function belong to Slo$^{1}_{0,n-1}$.

#### Proof

We verify the conditions of Definition 8. Consider any linear function $f(x)=\sum _{i=1}^n c_ix_i$, where without loss of generality, the coefficients satisfy the following monotonicity property

7 $$\begin{aligned} c_1\;\ge \; \cdots \; \ge \; c_n\;>0. \end{aligned}$$

Define the optimal level $A_n:=\{1^n\}$, and for all $j\in [0..n-1]$, define level

$$\begin{aligned} A_j:= \left\{ x \mid \sum _{i=1}^{j}c_i\le f(x)<\sum _{i=1}^{j+1}c_i\right\} . \end{aligned}$$

Trivially, $m\in {{\mathrm{poly}}}(n)$ and the last level $A_{n}$ contains the only optimal solution. For any other level $j\le n$ and any $x\in A_j$, there must exist, by the definition of the levels and the monotonicity of the coefficients, at least one bit-position $i\le j+1$ such that $x_i=0$. Flipping this bit-position produces an element in level $A_{\ge j+1}$. In other words, the Hamming distance to the upper levels is *O*(1). Finally, there are no deceptive pairs in the partition. Any linear function therefore satisfies the conditions for any setting of $\alpha$ and $\varepsilon ,$ including the most restrictive setting where $\alpha =1$, $\varepsilon =0$, and $r=n-1$.

For LeadingOnes, we use the canonical fitness-based partition into $m:=n+1$ levels

$$\begin{aligned} A_j&:= \{x \mid \textsc{LeadingOnes} (x)=j \}. \end{aligned}$$

Trivially, $m\in {{\mathrm{poly}}}(n)$. Furthermore, level $A_{n}$ contains the only optimal solution $1^n$, and for any other levels $j\le n$, and any $x\in A_j$, flipping the first 0-bit produces an element in level $A_{j+1}$. Finally, there are by definition no deceptive pairs. LeadingOnes therefore satisfies the conditions for any setting of $\alpha$ and $\varepsilon ,$ including the most restrictive setting where $\alpha =1$ and $\varepsilon =0$.

For Jump$_{k,n}$, we choose the canonical fitness-based partition into $m:=n+1$ levels

$$\begin{aligned} A_j:= {\left\{ \begin{array}{ll} \{x \mid {\textsc{Jump}}_{k,n} (x) = j\} & {\mathrm{if}}\quad j\in [n],\\ \{x \mid {\textsc{Jump}}_{k,n} (x) = n+k \} & {\mathrm{if}}\quad j=n+1. \end{array}\right. } \end{aligned}$$

Trivially, $m\in {\mathrm{poly}}(n).$ The final level $A_m=\{1^n\}$ contains the only global optimum.

For all levels $j\in [k-1]$, any element $x\in A_j$, contains at least one 1-bit, and flipping this into a 0-bit gives a bit string $x'\in A_{j+1}$. For all levels $j\in [k\dots n-1]$, any element $x\in A_j$ contains at least one 0-bit, and flipping this into a 1-bit gives a bit string $x'\in A_{j+1}$. At last, any element $x\in A_{n}$ contains exactly $k=O(1)$ 0-bits, and flipping all of these into 1-bits gives the optimal bit string $x'\in A_{n+1}$. Finally, by the definition of the levels, there are no deceptive pairs. The function therefore satisfies the conditions for any setting of $\alpha$ and $\varepsilon$, including the most restrictive one where $\alpha =1$, $\varepsilon =0$, and $r=n-1$.

The function Plateau $_{k,n}$ (see e.g.,  [2]) is similar to Jump$_{k,n}$, except that the “gap” is replaced by a “plateau” of equal fitness. We choose the canonical fitness-based partition into $m:=n-k+1$ levels

$$\begin{aligned} A_j:= {\left\{ \begin{array}{ll} \{x \mid {\textsc{Plateau}}_{k,n} (x) = j\} & {\mathrm{if}}\quad j\in [0..n-k],\\ \{x \mid {\textsc{Plateau}}_{k,n} (x) = n \} & {\mathrm{if}}\quad j=n-k+1. \end{array}\right. } \end{aligned}$$

Trivially, $m\in {{\mathrm{poly}}}(n).$ The final level $A_m=\{1^n\}$ contains the only global optimum. For all levels $j\in [0..n-k-1]$, any element $x\in A_j$, contains at least one 0-bit, and flipping this into a 1-bit gives a bit string $x'\in A_{j+1}$. Any element in $A_{n-k}$ contains at most $k=O(1)$ 0-bits, and flipping all of these bit-positions gives the optimal solution $1^n$. Finally, by the definition of the levels, there are no deceptive pairs. The function therefore satisfies the conditions for any setting of $\alpha$ and $\varepsilon$, including the most restrictive one where $\alpha =1$, $\varepsilon =0$, and $r=n-1$. $\square$

### Needle and Deceptive Problems

The problems discussed so far in this subsection are “non-deceptive” and belong to Slo$^\alpha _{\varepsilon ,r}$ for a small $\varepsilon =0$ and large $\alpha =1$. These problems can be optimised in expected polynomial time by appropriately configured evolutionary algorithms. We now consider functions at the other extreme end of the problem hierarchy. Our intuition is that when we relax the sparsity assumption on local optima (i.e., increase $\varepsilon$) and relax the density assumption on fitness valleys (i.e., decrease $\alpha$), then the problem class Slo$^\alpha _{\varepsilon ,r}$ contains harder problem instances. To test this intuition, we will place problem classes which are provably hard for all search heuristics into the Slo$^\alpha _{\varepsilon ,r}$ hierarchy.

We say that a pseudo-Boolean function *f* belongs to the Needle problem class if and only if there exist two constants $c_1>c_2$ and a bit string $z\in \{0,1\}^n,$ such that

$f(z)=c_1$ and $f(x)=c_2$ for all $x\ne z$. Droste, Jansen and Wegener showed that the black-box complexity of Needle is $2^{n-1}+1/2$ [20]. We will describe a broader class of “deceptive” problems formally defined by the conditions of Lemma 17. Informally, these problems have the property that search points near the unique global optimum $x^*$ have inferior fitness to some *x* which is far from the optimum. The function class Needle belongs to this class. However not all such deceptive functions are as difficult as Needle, e.g., Trap has a low black-box complexity [20].

#### Lemma 17

Let $f:\{0,1\}^n\rightarrow \mathbb {R}$ be any pseudo-Boolean function with a unique global optimum $x^*$. Assume there exists a function $d(n)=\omega (1/n)$ and a bit string *x* with $H(x,x^*)\ge d(n)n$, such that for all bit strings *y* where $1\le H(y,x^*)<d(n)n$, $f(x)\ge f(y)$ then $f\not \in \textsc{Slo}^{d(n)}_{1,1}$. If additionally it holds for all pairs of bit strings (*z*, *y*) where $H(z,x)=1$ and $1\le H(x^*,y)\le d(n)n$ that $f(z)\ge f(y)$, then $f\not \in \textsc{Slo}^{0}_{\varepsilon ,1}$ for any $\varepsilon <1$.

#### Proof

We prove the first part of the statement. Suppose by contradiction that $f\in \textsc{Slo}^{\alpha }_{1,1}$ for $\alpha =d(n)$. Consider any partition $(A_1,\ldots ,A_m)$ which satisfies Definition 8 for these parameters. Choose any bit string $y\in {\mathrm{argmax}}_{u\in A_{m-1}} H(u,x^*)$, i.e., any search point with maximal distance to the optimum among the elements in level $A_{m-1}$. Since *y* is an element in $A_{m-1}$ with maximal distance to $x^*$, any element $y'$ in $S_1(y)\cap A_{\ge m-1}$ must have Hamming distance $H(x^*,y')<H(x^*,y)$ to the optimum. Every neighbour of *y* is either closer to $x^*$ by one or further from it by one, and there are exactly $H(y, x^*)$ neighbours that are closer. Since only these neighbours can be in $A_{\ge m - 1}$, we have

8 $$\begin{aligned} |S_1(y)\cap A_{\ge m-1}|\le H(x^*,y) =O(1)< d(n) n, \end{aligned}$$

where the last inequality is due to condition 1 and the assumption $d(n)=\omega (1/n)$.

By condition 1 of Definition 8, since $H(x,x^*)\ge d(n)=\omega (1)$, search point *x* cannot belong to $A_{m-1}$, and must instead belong to some level $A_j$ with $j\le m-2$. Furthermore, $f(x)\ge f(y)$ holds by assumption of the lemma. Therefore, $(A_j,A_{m-1})$ is an *f*-deceptive pair, so condition 4 requires that

9 $$\begin{aligned} |S_1(y)\cap A_{\ge m-1}| \ge \alpha n = d(n)n. \end{aligned}$$

However, (8) contradicts with (9), and we conclude that $f\not \in \textsc{Slo}^{d(n)}_{1,1}$.

We now prove the second part of the statement. Define $C:=\{x\}\cup S_1(x)$. Suppose by contradiction that $f\in \textsc{Slo}^{\alpha }_{\varepsilon ,1}$ for some $\varepsilon <1$ and any $\alpha$. Consider any partition $(A_1,\ldots ,A_m)$ which satisfies Definition 8 for these parameters, and let $(A_{i_1},A_{j_1}),\ldots , (A_{i_u},A_{j_u})$ be the *f*-deceptive pairs.

We first claim that $(A_i,A_{m-1})$ is an *f*-deceptive pair for all levels $A_i$ where $A_i\cap C\ne \emptyset$. By assumption $H(C,x^*)=\omega (1),$ thus by condition 1 of Definition 8, $C\cap A_{m-1}=\emptyset$, i.e., every element in *C* is placed in an earlier level than $A_{m-1}$. Furthermore, $f(C)\ge f(A_{m-1})$ because $H(A_{m-1},x^*)=O(1)$ by condition 1. The claim therefore follows.

The claim above implies that $C\subseteq \cup _{v=1}^u A_{i_v}$. Furthermore, since condition 3 requires that $\cup _{v=1}^u A_{i_v}$ is $(\varepsilon ,r)$-sparse, Lemma 12 part 3 implies that the set *C* must also be $(\varepsilon ,r)$-sparse. However,

$$\begin{aligned} \varepsilon \cdot {n\atopwithdelims ()1}\ge |S_1(x)\cap C| = |S_1(x)| = {n\atopwithdelims ()1} \end{aligned}$$

contradicts that $f\in \textsc{Slo}^{\alpha }_{\varepsilon ,1}$ for $\varepsilon <1$, and the proof follows. $\square$

It is now straightforward to classify the Needle problem in the Slo$^\alpha _{\varepsilon ,r}$ problem hierarchy.

#### Corollary 18

For any constant $c\ge 1$, any $r< n$, any $\varepsilon <1$, and any function $d(n)=\omega (1/n)$, then

$$\begin{aligned} \textsc{Needle}&\subseteq \textsc{Slo}^{c/n}_{1,r} {\setminus} \left( \textsc{Slo}^{0}_{\varepsilon ,1} \cup \textsc{Slo}^{d(n)}_{1,1} \right) ,\\ \textsc{Jump} _k&\in \textsc{Slo}^{c/n}_{1,r} {\setminus} \left( \textsc{Slo}^{0}_{\varepsilon ,1} \cup \textsc{Slo}^{k/n}_{1,1} \right)\,\, \hbox{for any}\,\,k=\omega (1). \end{aligned}$$

#### Proof

The inclusion $\textsc{Needle} \cup \{\textsc{Jump} _k\}\subseteq \textsc{Slo}^{c/n}_{1,r}$ follows immediately from Lemma 10.

Consider any function $f\in \textsc{Needle}$, and assume without loss of generality that $x^*$ is its unique global optimum. To see that $f\not \in \textsc{Slo}^{0}_{\varepsilon ,1} \cup \textsc{Slo}^{d(n)}_{1,1}$, it suffices to notice that $f(x)=f(z)$ whenever $x\ne x^*$ and $y\ne x^*$, and the conditions of Lemma 17 and are satisfied.

We now consider $\textsc{Jump} _k$. Let *x* be any search point with $H(x,1^n)=k$. It is then easy to see that *x* satisfies the conditions of Lemma 17 for $d(n)=(k-1)/n$. $\square$

### No Free Lunch Function Classes

We continue classifying problems which are known to be hard for randomised search heuristics. In particular, we show that problem classes that satisfy the assumptions of the No Free Lunch Theorem belong to Slo$^\alpha _{\varepsilon ,r}$ if and only if $\alpha$ is close to 0 and $\varepsilon$ is close to 1.

Informally, the No Free Lunch Theorem (NFL) for optimisation by Wolpert and Macready states that the average case runtime over all functions is the same for all search heuristics [43], i.e., the same runtime as random search. Note that the NFL theorem does not count re-evaluations of search points in the definition of runtime. To state this result precisely, we use the more general result in Theorem 19. For any sets *A* and *B*, where *B* is completely ordered, let $F_{A,B}$ be the function class containing all functions $f:A\rightarrow B$. A problem class $F\subseteq F_{A,B}$ is *closed under permutation* if for any $f\in F$ and any permutation $\sigma$ over *A*, the function $f_\sigma (x):=f(\sigma A)$ also belongs to *F*. Informally, we say that a problem class which is closed under permutation “lacks structure”. The theorem implies that if a search heuristic has polynomial average case expected runtime (not counting re-evaluation of search points) on a problem class with polynomial number of global optima (and therefore faster than random search which needs exponential time), then that problem class must have some “structure”.

#### Theorem 19

(Generalised NFL theorem Droste et al. [19]). Let *H* be an arbitrary (randomised or deterministic) search heuristic for functions $f\in F\subseteq F_{A,B}$ where *F* is closed under permutation. Let *r*(*H*) be the average (under the uniform distribution on *F*) number of search points evaluated by *H*, not counting re-evaluations, to find an optimum. Then *r*(*H*) is a value independent of *H*, i.e., *r*(*H*) is the same for all *H*.

The following theorem relates the No Free Lunch theorem to the Slo$^\alpha _{\varepsilon ,r}$ problem class. Let *F* be any class of pseudo-Boolean functions which is closed under permutation. Since the problem class $\textsc{Slo}^\alpha _{\varepsilon ,r}$ contains all pseudo-Boolean functions for $\alpha =0$ and $\varepsilon =1$, we clearly have $F\subseteq \textsc{Slo}^{0}_{1,r}$. However, we will see that for $\alpha$ close to 1 and $\varepsilon$ close to 0, appropriately configured evolutionary algorithms have polynomial worst case expected runtime on Slo$^\alpha _{\varepsilon ,r}$ (cf Theorems 45 and Corollary 46). In light of Theorem 19, *F* must contain some hard functions, and hence, we would not expect *F* to be a subset of Slo$^\alpha _{\varepsilon ,r}$ when $\alpha$ is close to 1 and $\varepsilon$ is close to 0. The following theorem confirms this intuition. The idea behind the proof is that in any such function class *F*, there must exist a “maximally deceptive” problem where the fitness of any non-optimal search point increases with the Hamming distance to the optimum, and then apply Lemma 17.

#### Theorem 20

Consider any non-empty class of pseudo-Boolean functions $F\subset \{ f \mid f : \{0,1\}^n\rightarrow \mathbb {R}\}$ in which every element $f\in F$ has a unique global maximum and where *F* is closed under permutation. Then for all constants $c\ge 1$ and any *r*, $F \subseteq \textsc{Slo}^{c/n}_{1,r}$ and for all $\varepsilon <1$ and any function $d(n)=\omega (1/n)$, $F \not \subseteq \textsc{Slo}^{d(n)}_{1,1} \cup \textsc{Slo}^{0}_{\varepsilon ,1}.$

#### Proof

The inclusion $F \subseteq \textsc{Slo}^{c/n}_{1,r}$ follows immediately from Lemma 10.

To prove the second statement of the theorem, consider any function $g\in F.$ By assumption, *g* has a unique global optimum for some search point $z^{(0)}$. Define $f_{\mathrm{max}}:= g(z^{(0)})$. Hence, we have $\forall z\in \{0,1\}^n, \ z\ne z^{(0)}, \ g(z^{(0)}) = f_{\mathrm{max}}> g(z).$

To complete the proof, since *F* is closed under permutation, it suffices to construct a permutation $\sigma :\{0,1\}^n\rightarrow \{0,1\}^n$ such that the function $f=g_{\sigma }\in F$ does not belong to $\textsc{Slo}^{c/n}_{1,r}$.

The permutation $\sigma$ will be constructed such that *f* is “deceptive”. Let $y^{(0)}$, $y^{(1)},\ldots ,y^{(2^n-1)}$ be an enumeration of $\{0,1\}^n$ sorted according to increasing Hamming distance to $1^n$, i.e., this sequence satisfies for all $i,j\in [0..2^{n}-1]$

10 $$\begin{aligned} H(y^{(i)},1^n) \ge H(y^{(j)},1^n) \Longrightarrow i \ge j. \end{aligned}$$

Let $z^{(1)},\ldots ,z^{(2^n-1)}$ be an enumeration of $\{0,1\}^n{\setminus} \{z^{(0)}\}$ sorted according to increasing *g* value. Hence, we have

11 $$\begin{aligned} \forall i,j\in [2^n-1]: i\ge j \Longrightarrow g(z^{(i)}) \ge g(z^{(j)}). \end{aligned}$$

Define the permutation $\sigma : \{0,1\}^n\rightarrow \{0,1\}^n$ by assuming for all $i\in [0..2^{n}-1]$

12 $$\begin{aligned} \sigma (y^{(i)}) := z^{(i)}, \end{aligned}$$

and define $$f:= g_{\sigma }$$.

We claim that the function *f* satisfies $f(1^n)=f_{\mathrm{max}}$ and for all $x,y\in \{0,1\}^n{\setminus} \{ 1^n\}$,

13 $$\begin{aligned} H(x,1^n)>H(y,1^n) \Longrightarrow f(x)\ge f(y). \end{aligned}$$

The first part of the claim is true by definition. To see that the second part of the claim is also true, consider any $i,j\in [2^n-1]$. Then by (10)

$$\begin{aligned} H(y^{(i)},1^n) > H(y^{(j)},1^n) \Longrightarrow i \ge j \end{aligned}$$

(by (11))

$$\begin{aligned}\Longrightarrow g(z^{(i)}) \ge g(z^{(j)}) \end{aligned}$$

(by the definition of the permutation $\sigma$ in (12))

$$\begin{aligned}\Longrightarrow g(\sigma (y^{(i)})) \ge g(\sigma (y^{(j)})) \end{aligned}$$

(by the definition of function *f*)

$$\begin{aligned}\Longrightarrow f( y^{(i)}) \ge f(y^{(j)}). \end{aligned}$$

Since *F* is closed under permutation, $f\in F$. Note that for $x^*=1^n$ and all *z* and *y* where $H(z,x^*)\ge d(n)n > H(y,x^*)\ge 1$, we have $f(z)\ge f(y)$. The proof now follows by applying Lemma 17 with respect to $x=y^{(2^n-1)}=0^n$ and *d*(*n*). $\square$

### Problems in Combinatorial Optimisation

This section gives some example classifications for instances of well-known NP-hard problems. These instances contain local optima and they fall into to immediate parts of the SLO-hierarchy.

#### Knapsack Problem

Consider a knapsack with weight capacity *W* and a set of *n* items where each item $i\in [n]$ is associated with a weight $w_i\ge 0$ and value $p_i\ge 0$. The goal of 0/1-Knapsack Problem (KP) is to find the most valuable items to put in the knapsack while respecting its capacity, i.e., maximise $\sum _{i=1}^n p_i x_i$ subject to the constraint $\sum _{i=1}^n w_i x_i \le W,$ where each variable $x_i \in \{0,1\}$ indicates the selection of item *i*. The problem can be cast into pseudo-Boolean optimisation by maximising the following fitness function.

$$\begin{aligned} f_{\textsc{Knapsack}}(x):={\left\{ \begin{array}{ll} \sum _{i=1}^n -x_i & \hbox{if}\quad \sum _{i=1}^n w_i x_i>W,\\ \sum _{i=1}^n p_i x_i & \mathrm{otherwise.} \end{array}\right. } \end{aligned}$$

We consider a family of KP instances, denoted $\textsc{Knapsack} _{u,v}$, which is given by Table 2 and is parameterised by the dimension *n* and two constants $u, v \in (0,1)$ with $\ell :=(1-v)n\in \mathbb {N}$.

**Table 2:** Characteristics of $\textsc{Knapsack} _{u,v}$ instances.

*i*	$p_i$	$w_i$	*W*
$i=1$	$(1-u)(1-v)n$	$(1-v)n$	$(1-v)n$
$2\le i \le (1-v)n$	1	1	
$(1-v)n < i \le n$	0	0	

For any $z \in \{0,1\}^{n-\ell }$ the solution $0 1^{\ell -1} z$ with fitness $\ell -1$ is a global optimum, and the solution $10^{\ell -1} z$ with fitness $p_1=(1-u)\ell$ is a local optimum for sufficiently large *n*. Particularly, a local optimum selects the high value item which is also the heaviest, and possibly some “dummy” items.

##### Proposition 21

The fitness function $f_{\textsc{Knapsack}}$ of $\textsc{Knapsack} _{u, v}$ belongs to

$$\textsc{Slo}^{u(1-v)+v}_{v,n-1} $$.

##### Proof

By Lemma 6, It suffices to show inclusion for $r_{\max }=\lfloor n/2 \rfloor$. We consider the following partition of the search space into $n + \ell$ levels, where $\ell$ is defined in the paragraph before the proposition.

$$\begin{aligned} A_j := {\left\{ \begin{array}{ll} \left\{ 1yz \mid y \in \{0,1\}^{\ell -1}, z \in \{0,1\}^{n-\ell }, |y|_1>0, |yz|_1 = n-j \right\} & \hbox{if}\quad 1 \le j \le n-1, \\ \left\{ 1yz \mid y \in \{0,1\}^{\ell -1}, z \in \{0,1\}^{n-\ell }, |y|_1=0 \right\} & \hbox{if}\quad j=n, \\ \left\{ 0yz \mid y \in \{0,1\}^{\ell -1}, z \in \{0,1\}^{n-\ell }, |y|_1=j-n-1\right\} & \hbox{if}\quad n < j \le n+\ell . \end{array}\right. } \end{aligned}$$

Then it holds for the fitness of $x \in A_{j}$ that:

$$\begin{aligned} f_{{\textsc{Knapsack}}}(x) = {\left\{ \begin{array}{ll} -(n - j + 1) & {\mathrm{if}}\quad 1\le j\le n-1,\\ (1-u)\ell & {\mathrm{if}}\quad j=n,\\ j - n - 1 & {\mathrm{if}}\quad n<j\le n+\ell . \end{array}\right. } \end{aligned}$$

**Fig. 2:**
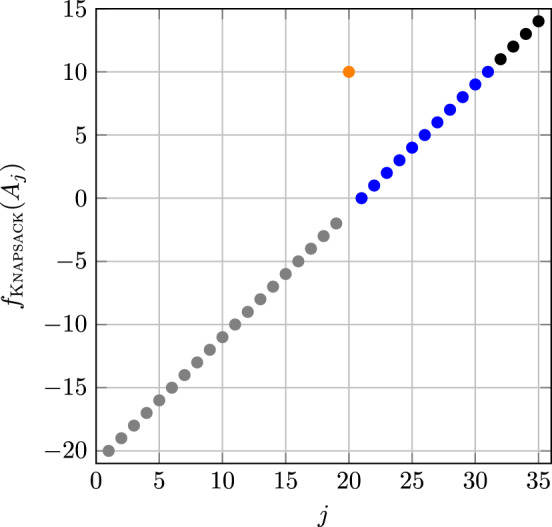
$f_{\textsc{Knapsack}}(x)$ in the level partition for $n=20$, $u=1/3$ and $v=1/4$. The level containing all local optima corresponds to the orange point while those of the fitness valley are in blue.

An illustration of this function for $n=20$, $u=1/3$ and $v=1/4$ is shown in Fig. 2.

Clearly, the last level $A_{n + \ell }$ consists of all global optima. For every $x\in A_j$ with $j\in [n + \ell -1],$ there is an element of $A_{j+1}$ in Hamming distance 1.

The deceptive pairs are $(A_{n},A_{n+i}),$ where $1\le i \le (1-u)\ell + 1$, and any solution *x* from any valley level $A_{n+i},$ contains at least $u\ell$ zero-bits such that flipping any of these bits gives a solution in $A_{n+i+1}.$ Besides that, flipping any of the *vn* bits of *z* keeps the current level unchanged. This implies $(u\ell /n+v)=(u(1-v)+v)$-density of $A_{\ell +i}$ with respect to the union $\cup _{i\le j \le (1-u)\ell +1} A_{n+j}$.

On the other hand, the set $A_{\ell }$ of local optima has its first $\ell \ge (1-v)n$ bits fixed, thus it follows from Lemma 15 that the set is $(v,\lfloor n/2\rfloor )$-sparse. $\square$

#### Weighted Independent Set Problem

Let $G=(V,E)$ be a graph where the vertices are numbered, i.e., $V=[n]$, and weighted by a function $w:V \rightarrow \mathbb {R}^{+}$. Then any bit string $x\in \{0,1\}^n$ encodes a subset $V(x) \subseteq V$, i.e., $x_i=1$ if vertex *i* belongs to *V*(*x*) and $x_i=0$ otherwise. *V*(*x*) is called an *independent set* if for any edge $\{u, v\}\in E,$ *V*(*x*) does not contain both of its endpoints, i.e., $\{u, v\}\not \subseteq V(x)$. The Maximum Weight Independent Set Problem (MISP) asks for an independent set *V*(*x*) of maximum weight $\sum _{i=1}^{n}w_i x_i$. MISP is equivalent to the Weighted Vertex Cover Problem (VCP). A subset $C\subseteq V$ is called a *cover* if for each $\{u,v\}\in E$ either $u\in C$ or $v\in C$ or both of them are in *C*. The VCP asks for a cover of minimum weight. *V*(*x*) is an independent set iff $V({\overline{x}}):=V{\setminus} V(x)$ is a cover.

Solving MISP is equivalent to the maximisation of

$$\begin{aligned} f_{{\textsc{MISP}}}(x):= {\left\{ \begin{array}{ll} \sum _{i=1}^{n} w(i) x_i & {\mathrm{if}}\quad {V}({x})\,{\hbox{is an independent set}},\\ -\sum _{\{u,v\}\in E} x_u x_v & {\mathrm{otherwise}}. \end{array}\right. } \end{aligned}$$

This is because when *V*(*x*) is an independent set, the function maximises the sum of weights of vertices in the subset (i.e., $V({\overline{x}})$), otherwise it minimises the number of edges contained in *V*(*x*).

A claw (or star) graph parameterised by *n*, denoted $K_{1,n}$, is a complete bipartite graph which consists of *n* vertices, numbered from 1 to *n*, in one subset and of a vertex, numbered $n+1$, in the other subset. There are all edges that connect the vertices of one subset to those of the other subset, but no edge within a subset. We consider a family of instances of MISP on $K_{1,n}$ parameterised by $u,v \in (0,1)$, $v\le u,$ and *n*, and denoted $\textsc{MISP} ^{u,v}(K_{1,n})$, such that

14 $$\begin{aligned} w(n+1)= (1-u)S,\quad \mathrm{where}\quad S:=\sum _{i=1}^{n}w(i) ,\end{aligned}$$

15 $$\begin{aligned} \frac{w(i)}{w(j)}\le \frac{u}{v}\,\, \text{for all}\, i, j \in [n] . \end{aligned}$$

Note that (15) can also be written as

16 $$\begin{aligned} u \min _{i\in [n]}\{w(i)\}&\ge v \max _{i\in [n]}\{w(i)\} . \end{aligned}$$

The global optimum of $\textsc{MISP} ^{u,v}(K_{1,n})$ is the independent set $x^*:=1^n 0$ with fitness (and weight) *S*. The local optimum is $\overline{x^*}:=0^n 1$ with fitness (and weight) $(1-u)S$.

Notice that the expected optimisation time of the Randomised Local Search (RLS) (i.e., using Hamming-neighbours of distance 1) is infinite on $\textsc{MISP} ^{u,v}(K_{1,n})$ due to a positive chance of a bad initialisation. The following result shows that $\textsc{MISP} ^{u,v}(K_{1,n})$ belongs to Slo$^\alpha _{\varepsilon ,r}$ class, hence it can be solved efficiently with non-elitist EAs.

##### Proposition 22

The fitness function $f_{MISP}$ for $\textsc{MISP} ^{u,v}(K_{1,n})$ belongs to $\textsc{Slo}^{v}_{\frac{1}{n+1},n-1}$.

##### Proof

By Lemma 6, it suffices to show membership for the parameter $r_{\max }=\lfloor \frac{n+1}{2} \rfloor$. Assuming w. l. o. g. that $w(1) \ge w(2) \ge \dots \ge w(n)$, we partition the search space $\{0,1\}^{n+1}$ into $2n+2$ levels as follows:

$$\begin{aligned} A_j:= {\left\{ \begin{array}{ll} \left\{ y1 \mid y \in \{0,1\}^n, |y|_1 = n-j\right\} & \hbox{if}\quad 0\le j\le n, \\ \left\{ y0 \mid y \in \{0,1\}^n, \displaystyle \sum _{i=1}^{j-n-1}w(i) \le \sum _{i=1}^n w(i) y_i< \sum _{i=1}^{j-n}w(i)\right\} & \hbox{if}\quad n< j\le 2n,\\ \left\{ 1^n 0 \right\} & \hbox{if}\quad j=2n+1, \end{array}\right. } \end{aligned}$$

and hereafter we use string *y* to denote the first *n* bits of *x*.

It is clear that the last level $A_{2n+1}$ contains the unique global optimum. It is also clear that for all search points $x \in A_{j}$ with $j\le n$, there exists an $x' \in A_{\ge j+1}$ with $H(x,x')=1$, e.g., it suffices to flip the “1” at position $n+1$ to a “0”. For $j>n$, the same statement also holds because among the first $j-n$ bits of *y* in the level definition, there must be at least one “0” because otherwise we would have $\sum _{i=1}^{j-n}w(i) y_i=\sum _{i=1}^{j-n}w(i)$ which contradicts the level definition. Thus flipping the left-most “0”, which has the weight at least $w(j-n)$ by our assumption, guarantees to leave the $A_j$ to a higher level.

It follows from (14) that for any $x \in A_{j}$ we have:

$$\begin{aligned} f(x)\in {\left\{ \begin{array}{ll} \{-(n-j)\} & \hbox{if}\quad 0\le j\le n-1, \\ \{(1-u)S\} & \hbox{if}\quad j=n, \\ \left[ \sum _{i=1}^{j-n-1}w(i), \sum _{i=1}^{j-n}w(i)\right) & \hbox{if}\quad n< j\le 2n,\\ \{S\} & \hbox{if}\quad j=2n+1. \end{array}\right. } \end{aligned}$$

For $j\ge 1$, it holds for all $x \in A_{n+j}$ that *V*(*x*) is an independent set and $f(x)=$ $\sum _{i=1}^{n} w(i) y_i$. So if $x \in A_{n+j}$ and $\sum _{i=1}^{n} w(i)y_i$ $\le (1-u) S$, we have that: (i) $f(x)\le f(0^n 1)$, that is, $(A_n,A_{n+j})$ is a deceptive pair, and (ii) $\sum _{i=1}^{j-1}w(i)\le f(x)\le (1-u)S$. Therefore, the deceptive pairs are $(A_n, A_{n+j})$ for $j\in [n]$ satisfying $\sum _{i=1}^{j-1}w(i) \le (1-u)S$.

It follows from (16) that $u w(n) \ge v w(1)$, so for a valley solution $x:= y0 \in A_{n+j}$ it holds

$$\begin{aligned} \sum _{i=1}^{n} w(i) (1-y_i) \ge u S = u \sum _{i=1}^{n} w(i) \ge u \sum _{i=1}^{n} w(n) = u \cdot n \cdot w(n) \end{aligned}$$

and

$$\begin{aligned} \sum _{i=1}^{n} w(i) (1-y_i) \le (n-|V(x)|) \cdot w(1) \le (n-|V(x)|) \cdot (u/v) \cdot w(n). \end{aligned}$$

Combining these gives $(n-|V(x)|) \cdot (u/v) \cdot w(n) \ge u \cdot n \cdot w(n)$, or equivalently, $n-|V(x)| \ge v n$. In other words, *x* has at least *vn* zeros in substring *y*, thus flipping any of these bits improves *f*(*x*) and keeps the search point at the same level or higher, i.e., in $\cup _{k\ge j} A_{n+k}$. Therefore, we have that the fitness valley is *v*-dense.

On the other hand, the set $A_n$ contains only the local optimum, and by Lemma 15 it is $(1/(n+1),\lfloor (n+1)/2\rfloor )$-sparse, as our problem is defined on bit strings of length $n+1$. $\square$

We can also consider the family of instances of the VCP on $K_{1,n}$ parameterised by $u,v \in (0,1)$, $v\le u,$ and *n*, and denote it by $\textsc{VCP} ^{u,v}(K_{1,n})$. Analogously to the MISP fitness function $f_{MISP}$ for $\textsc{MISP} ^{u,v}(K_{1,n})$ one can introduce a fitness function for the VCP:

$$\begin{aligned} f_{{\textsc{VCP}}}(x):= {\left\{ \begin{array}{ll} \sum _{i=1}^{n}(1-x_i)w(i) & {\mathrm{if}}\quad {V}({x})\,{\hbox{is a cover}}\\ -\sum _{(u,v)\in E}(1-x_u)(1-x_v) & {\mathrm{otherwise}}, \end{array}\right. } \end{aligned}$$

as a corollary to Proposition 22, due to the invariance of density and sparsity of any set of bit strings under the inversion of all bit positions, we have

##### Proposition 23

The fitness function $f_{VCP}$ for $\textsc{VCP} ^{u,v}(K_{1,n})$ belongs to $\textsc{Slo}^{v}_{\frac{1}{n+1},n-1}$.

The following subclass of $\textsc{VCP} ^{u,v}(K_{1,n})$ was used in Chvátal [4] as an example where the logarithmic approximation ratio of the greedy algorithm is asymptotically tight: $w(i)=1/i$ for $i\in [n]$ and $w(n+1)=1+o(1)$. Thus the subclass has $S=\sum _{i=1}^{n}1/i =: H_n$, $u=1-(1+o(1))/H_n$ (so (14) is satisfied for $n\ge 2$) and $v=u/n$ (so (15) is satisfied), and therefore it belongs to $\textsc{Slo}^{\frac{1}{n}-\frac{1+o(1)}{n H_n}}_{\frac{1}{n+1},\lfloor \frac{n+1}{2} \rfloor }$.

### Randomly Perturbed Problems

In the previous sections, we classified theoretical benchmark problems commonly studied in runtime analysis of evolutionary algorithms. While these problems capture important fitness landscape characteristics, we would like to understand more complex fitness landscapes, particularly highly multi-modal landscapes. A popular approach to construct a harder fitness landscape which is to depart from a fitness function *f* with well-understood structure, then redefine fitness values on a (possibly random) perturbation set *B*.

So-called frozen noise models (also called noisy landscape with fitness caching) are popular in evolutionary computing [21, 25, 28, 38], and similarly the rough Mt. Fuji model is popular in population genetics [1, 35]. A difference with our approach is that instead of introducing frozen (or cached) noise for every search point and controlling the ruggedness with the scaling factor (see e.g., [35] or [21]), we allow the function to be completely redefined on the perturbation set *B*.

#### Definition 24

Given $f:\{0,1\}^n\rightarrow \mathbb {R}$, a subset $B\subset \{0,1\}^n$, and a threshold $f_0<\max _x \{f(x)\}$. Let $\mathcal {G}(f,f_0,B)$ consist of all functions $g:\{0,1\}^n\rightarrow \mathbb {R}$ such that for some function $h:\{0,1\}^n\rightarrow (-\infty ,f_0]$,

$$\begin{aligned} g(x):= {\left\{ \begin{array}{ll} h(x)& \hbox{if}\quad x\in B \wedge f(x) \le f_0,\quad\mathrm{and}\\ f(x) &\mathrm{otherwise}. \end{array}\right. } \end{aligned}$$

Intuitively, the class $\mathcal {G}(f,f_0,B)$ contains any function which has the same function-value as *f* on points outside the perturbation set *B*. The threshold $f_0$ ensures that no function in $\mathcal {G}(f,f_0,B)$ has perturbed search points with *f*-value exceeding $f_0$. As a consequence, the functions in the perturbed class have the same optima as *f*.

Following [28], we construct the subset *B* by fixing a probability *p* for independently considering the inclusion of each bit string into *B*. Hence, in expectation, we perturb $p2^n$ elements. Note that once the set *B* is constructed, we can consider the resulting function *g* as “frozen”. This type of frozen noise is related to analysis of EAs’ performance on fitness functions with stochastic noise, see e.g.,  [7], where non-elitist EAs have been shown efficient.

#### Definition 25

For $n\in \mathbb {N}$ and $p\in (0,1)$, let $\mathcal {V}(n,p)$ be the distribution over $\mathcal {P}(\{0,1\}^n)$, such that if $B\sim \mathcal {V}(n,p)$, then the event $x\in B$ holds independently for all $x\in \{0,1\}^n$ with probability *p*.

We will later see that the classification of the perturbed function class depends on the sparsity of *B*. The following lemma shows that decreasing the probability *p* increases the probability that the perturbation set *B* is sparse.

#### Lemma 26

For any $\varepsilon \in (0,1]$, $r_{\max }\in [n-1]$, and $p<\varepsilon /e$, a random set $B\sim \mathcal {V}(n,p)$ is $(\varepsilon ,r_{\max })$-sparse with probability at least $1-2^nr_{\max }(ep/\varepsilon )^{\varepsilon n}$.

#### Proof

For any $r\in [r_{\max }]$, let $K:={n\atopwithdelims ()r}$ and $k:=\varepsilon K\ge \varepsilon n$. We will first estimate the probability that $|B\cap S_r(x)|> k$ for an arbitrary *x*. For an arbitrary subset $C\subseteq S_r(x)$ of size *k*, the probability that all elements in *C* belong to *B* is $p^{k}$. By a union bound over all ways of selecting such a subset *C*, we get

$$\begin{aligned} \Pr \left( |B\cap S_r(x)|>k\right) \le {K\atopwithdelims ()k}p^k \le \left( \frac{epK}{k}\right) ^k =\left( \frac{ep}{\varepsilon }\right) ^k \le \left( \frac{ep}{\varepsilon }\right) ^{\varepsilon n}, \end{aligned}$$

where the last inequality follows because $p<\varepsilon /e$ and $k\ge \varepsilon n$. The proof is now complete by taking the union bound over all $2^n$ choices of *x* and $r_{\max }$ choices of *r*. $\square$

We are now ready to show how perturbing a function as described above changes its position in the hierarchy. Informally, Theorem 27 states that perturbing a function *f* belonging to $\textsc{Slo}^\alpha _{\varepsilon ,r}$ on a $\delta$-sparse perturbation set *B* leads to a perturbed function class $\mathcal {G}(f,f_0,B)$ where the density parameter is decreased by $\delta$ and the sparsity parameter is increased by $\delta$. Recall from Lemma 9 and Fig. 1 that decreasing the density parameter $\alpha$ and increasing the sparsity parameter $\varepsilon$ lead to a larger problem class containing potentially harder problems.

Note that those levels of the partition that originally are not part of a deceptive pair may be perturbed, and thus become part of a deceptive pair (cf Definition 7) for which conditions (3) and (4) of Definition 8 become relevant. We therefore need the additional assumption that any level $A_j$ with *f*-value less than $f_0$ must be $\alpha$-dense with respect to $A_{\ge j}$, i.e., the union of $A_j$ and higher levels.

#### Theorem 27

Let $0\le \varepsilon <1-\delta$, $0<\delta <\alpha \le 1$ with $\delta \le 1-1/n$, $r\ge 1$, and $f_0\in \mathbb {R}$. Let *B* be any $(\delta ,r)$-sparse set. Assume that $f\in \textsc{Slo}^\alpha _{\varepsilon ,r}$ for some level partition $(A_1,\dots ,A_m)$, and that

1. $$f(A_m)>f_0$$,
2. for any $A_j$ with $\min _{x\in A_j}f(x)\le f_0$,
  - the set $A_{j}$ is $\alpha$-dense with respect to $A_{\ge j}$,
  - for all $x\in A_j \cap B$ there exists $y\in A_{\ge j+1}{\setminus} B$ such that $H(x,y)=O(1)$.

Then $$\mathcal {G}(f,f_0,B)\subseteq \textsc{Slo}^{\alpha -\delta }_{\varepsilon +\delta ,r} $$.

#### Proof

We first claim that we can assume $f(x)\le f_0$ for all $x\in B$. Suppose otherwise, that *B* has a non-empty subset $B'=\{x\in B\mid f(x)>f_0\}$ of elements with strictly higher *f*-value than $f_0$. Note that the function *g* in Definition 24 keeps the original fitness of *f* for all points in $B'$, in other words, $\mathcal {G}(f,f_0,B)=\mathcal {G}(f,f_0,B{\setminus} B')$.

Furthermore, by Lemma 12, (3) $B{\setminus} B'$ is also $(\delta ,r)$-sparse. In the rest of this proof we will assume that $f(x)\le f_0$ for all $x\in B$, which also implies

17 $$\begin{aligned} \max _{x\in B} g(x) = \max _{x\in B} h(x) \le f_0. \end{aligned}$$

Now, consider any function $g\in \mathcal {G}(f,f_0,B)$. To prove that $g\in \textsc{Slo}^{\alpha -\delta }_{\varepsilon +\delta ,r}$, we construct the following new level partition $(A'_0,A'_1,\ldots ,A'_m)$ from the level partition $(A_1,\ldots ,A_m)$, then verify the conditions of Definition 8 for *g* on this new partition.

$$\begin{aligned} A'_0&:= B\\ A'_j&:= A_j {\setminus} B\, \text{for all}\, j \in [m]. \end{aligned}$$

By the level definition and by assumption 1 of this theorem regarding $f_0$, $A_m=A'_m$. Hence, condition 1 of Definition 8 is satisfied, that is,

$$\begin{aligned} A'_m=\{x\in \{0,1\}^n\mid \forall y\in \{0,1\}^n, g(x)\ge g(y)\}. \end{aligned}$$

We now consider condition 2 of Definition 8. For any level $j\in [0..m-1]$, and any $x\in A'_j$, we need to prove that there exists an element $y\in A'_{\ge j+1}$ such that $H(x,y)=O(1)$. We will distinguish between two cases.

Case 1 ($j=0$): The first level $A'_0=B$, is by assumption $(\delta ,r)$-sparse. Then by Definition 5, for all $x\in A'_0$, $|S_1(x)\cap A'_0|\le \delta n$. Therefore, *x* has at least $(1-\delta )n \ge 1$ Hamming-neighbours in $\{0,1\}^n{\setminus} A'_0 = A'_{\ge 1}$. So level $A'_0$ satisfies condition 2 of Definition 8.

Case 2 (any $j\in [m-1]$): By assumption, $(A_1,\ldots ,A_m)$ satisfies condition 2 in Definition 8, hence for every $x\in A'_j\subseteq A_j$ there exist an element $y\in A_k$ for some $k>j$ such that $H(x,y)=O(1)$. We distinguish between two sub-cases. In the case that $y\not \in B$, then $y\in A'_k$ and *x* satisfies the condition 2 with respect to *y*. Otherwise, in the case $y\in B$, we first note that $k<m$ since $A_m\cap B=\emptyset$ by assumption 1, thus there are still higher levels than $A_k$. Furthermore, if $\min _{x\in A_k} f(x)\le f(y)\le f_0$, then by assumption 2 (b) in this theorem, there exists an element $z\in A_{\ge k+1}{\setminus} B=A'_{\ge k+1}$ such that $H(y,z)=O(1)$. By the triangle inequality, $H(x,z)\le H(x,y)+H(y,z)=O(1)$, and *x* satisfies condition (2) with respect to *z*.

Condition 2 of Definition 8 is therefore satisfied in all cases.

We now consider conditions 3 and 4 of Definition 8. Suppose that $(A_i,A_j)$ is not *f*-deceptive for some $j>i\ge 1$. Then for all $x\in A_i$ and for all $y\in A_j$, $f(x)<f(y)$. This immediately implies that for all $x\in A'_i\subseteq A_i$ and all $y \in A'_j\subseteq A_j,$ we have $g(x)=f(x)<f(y)=g(y)$. Hence, $(A'_i,A'_j)$ is not *g*-deceptive. Therefore, any *g*-deceptive pair must either be a pair $(A'_0,A'_j)$ involving the new level $A'_0$, or a pair $(A'_{i_v},A'_{j_v})$ corresponding to an *f*-deceptive pair $(A_{i_v},A_{j_v})$.

Consider the first type of deceptive pairs. For any $j\in [m]$ if the pair $(A'_0,A'_j)$ is *g*-deceptive, then

$$\begin{aligned} f_0 \ge \max _{x\in B} g(x) = \max _{x\in A'_0} g(x) \ge \min _{y\in A'_j} g(y) = \min _{y\in A'_j} f(y) \ge \min _{y\in A_j} f(y), \end{aligned}$$

where the first inequality follows from (17) and the last inequality follows because $A'_j\subseteq A_j$. Since $\min _{y\in A_j} f(y)\le f_0$, then by assumption 2 (a), the set $A_j$ is $\alpha$-dense with respect to $A_{\ge j}$. We have already shown that $A'_0$ is $(\delta ,r)$-sparse when checking condition 2 of Definition 8, hence by Lemma 13, the set $A'_j=A_j{\setminus} B$ is $(\alpha -\delta )$-dense with respect to $A'_{\ge j}=A_{\ge j}{\setminus} B$.

We now consider the second type of deceptive pair. Again by Lemma 13 and given that $A'_0$ is $(\delta ,r)$-sparse, for any *f*-deceptive pair $(A_{i_v},A_{j_v})$, the set $A'_{j_{v}}= A_{j_v}{\setminus} B$ is $(\alpha -\delta )$-dense with respect to $A'_{\ge j_v}=A'_{\ge j_v}{\setminus} B$.

Finally, note that $\cup _{v=1}^u A_{i_v}$ is $(\varepsilon ,r)$-sparse due to the assumption that $f\in \textsc{Slo}^\alpha _{\varepsilon ,r}$. Hence, by Lemma 12 (4) and given that $A'_0$ is $(\delta ,r)$-sparse, the union $\cup _{v=0}^u A'_{i_v} = (\cup _{v=0}^u A_{i_v})\cup A'_0$ is $(\varepsilon +\delta ,r)$-sparse.

We have proved that the partition $(A'_j)_{j\in [0..m]}$ satisfies all the required conditions of Definition 8 for the function *g*, hence the theorem follows. $\square$

As a special case of Theorem 27, we identify the position of perturbed  OneMax within the Slo$^\alpha _{\varepsilon ,r}$ hierarchy. Jorritsma, Lengler and Sudholt introduced the function $\textsc{disOM}_{d,p}$, also called “distorted  OneMax ” [28]. Departing from  OneMax, it adds a constant *d* to the function value independently for every search point with probability *p*. Due to the $f_0$-threshold constraint from Definition 24, disOM $_{d,p}$ does not immediately belong to our randomised class $\mathcal {G}(\textsc{OneMax},f_0,B)$ where $f_0\in \mathbb {R}$ and $B\sim \mathcal {V}(n,p)$. However, we can define a closely related function

$$\begin{aligned} \textsc{disOM}'_{d,p} (x):= {\left\{ \begin{array}{ll} \min (f_0,\textsc{disOM}_{d,p} (x)) & \hbox{if}\quad \textsc{ OneMax} (x)\le f_0,\quad\mathrm{and}\\ \textsc{ OneMax} (x) & \mathrm{otherwise}, \end{array}\right. } \end{aligned}$$

which clearly belongs to the class $\mathcal {G}(\textsc{ OneMax},f_0,B)$. Jorritsma et al. compared the fixed target performance with fitness target $n-k^*$, showing that the $(1,\lambda )$ EA can be a factor 1/*p* faster than the $(1+\lambda )$ EA when $p\in \omega (1/(n\log n))\cap n^{-\Omega (1)}$ and $k^*\in n^c$ for some $c\in (0,1)$. They left regimes with larger *p* open for future research.

Defining $\textsc{disOM}'_{d,p}$ with respect to the parameter $f_0=n-k^*$ leads to a comparable problem setting as disOM $_{d,p}$. The following corollary shows that the perturbed problem class $\mathcal {G}(\textsc{ OneMax},(1-\alpha )n,B)$ which contains $\textsc{disOM}'_{d,p}$ for $f_0=n-k^*$ and $\alpha :=k^*/n$ belongs with high probability to $\textsc{Slo}^{\alpha -\delta }_{\delta ,r}$. For the special case where $\delta$ and $\alpha$ are constants, the function class $\mathcal {G}(\textsc{ OneMax},(1-\alpha )n,\mathcal {V}(n,p))$ belongs to $\textsc{Slo}^{\alpha -\delta }_{\delta ,r}$ with overwhelmingly high probability when *p* is a sufficiently small constant. This is significant, because as will be shown in Theorem 45, such problems can be solved in expected polynomial time with appropriately configured non-elitist EAs.

#### Corollary 28

For $0<\delta <\alpha \le 1, \alpha >1/n$ and $B\sim \mathcal {V}(n,p)$ with $p<\left( \frac{\delta }{2e}\right) ^{1+\frac{1+\alpha }{\delta }}$, $\mathcal {G}(\textsc{ OneMax},(1-\alpha )n,B)\subset \textsc{Slo}^{\alpha -\delta }_{\delta ,r}$ with probability at least $1-n2^{-\Omega (\alpha n)}$.

#### Proof

Note first that by Lemma 26 and given $p<\left( \frac{\delta }{2e}\right) ^{1+\frac{1+\alpha }{\delta }}<\frac{\delta }{e}$, $r\le n-1<n$, and $\delta /2e<1/2$, *B* is $(\delta ,r)$-sparse with probability at least

$$\begin{aligned} 1-2^nr\left( \frac{ep}{\delta }\right) ^{\delta n}&= 1-r \left( 2\cdot \left( \frac{e}{\delta }\right) ^\delta \cdot p^{\delta }\right) ^{n}> 1-n \left( 2\cdot \left( \frac{e}{\delta }\right) ^\delta \cdot \left( \frac{\delta }{2e}\right) ^\delta \cdot \left( \frac{\delta }{2e}\right) ^{1+\alpha } \right) ^{n}\\&> 1-n \left( 2\cdot 2^{-\delta } \cdot 2^{-1-\alpha } \right) ^{n} = 1-n 2^{-(\delta +\alpha )n} = 1 - n 2^{-\Omega (\alpha n)}. \end{aligned}$$

We apply Theorem 27 with parameters $f_0:=(1-\alpha )n$ and $\varepsilon :=0$. By Theorem 16, $\textsc{ OneMax} \in \textsc{Slo}^{1}_{0,r} \subseteq \textsc{Slo}^{\alpha }_{\varepsilon ,r}$. This follows using the canonical partition defined by $x\in A_j$ if and only if $\textsc{ OneMax} (x)=j$. Clearly, this partition satisfies $f(A_n)>f_0$, i.e., assumption 1 of the theorem.

Furthermore, $\min _{x\in A_j} \textsc{ OneMax} (x)\le f_0$ implies $j\le f_0$. Any search point $x\in A_j$ for $j\le f_0$ has at least $\alpha n$ 0-bits. More formally, $|S_1(x)\cap A_{\ge j}|$ $=|S_1(x)\cap A_{j+1}|=n-j\ge \alpha n$. Hence, $A_j$ is $\alpha$-dense with respect to $A_{\ge j}$ or assumption 2 (a) is satisfied.

Finally, we claim that with probability at least $1-n2^{-\Omega (\alpha n)}$ assumption 2 (b) is satisfied, that is, for all $j\le f_0$, and for all $x\in A_j$ ($\supseteq A_j \cap B$), there exists $y\in A_{j+2}{\setminus} B$ such that $H(x,y)=2$. Any such $x\in A_j$ has $k:=n-j\ge \alpha n$ 0-bits, and there are therefore ${k\atopwithdelims ()2}\ge k^2/4$ elements $y\in A_{j+2}$ with $H(x,y)=2$. The probability that all of these belong to *B* is no more than

$$\begin{aligned} p^{k^2/4}< \left( \left( \frac{\delta }{2e}\right) ^{1+\frac{1+\alpha }{\delta }}\right) ^{k^2/4} <\left( \frac{\delta }{e}\right) ^{k^2/4}\le \left( \frac{\alpha }{e}\right) ^{k^2/4}, \end{aligned}$$

where the second inequality holds because $\delta<1<2e$ and $1+(1+\alpha )/\delta >1$.

By a union bound over the elements of $A_j$, the probability that this event holds for any of the elements in $A_j$ is at most

$$\begin{aligned} {n\atopwithdelims ()k}\left( \frac{\alpha }{e}\right) ^{k^2/4}&\le \left( \frac{en}{k}\right) ^{k}\left( \frac{\alpha }{e}\right) ^{k^2/4} \le \left( \frac{en}{\alpha n}\right) ^{k}\left( \frac{\alpha }{e}\right) ^{k^2/4}\\&= \left( \frac{\alpha }{e}\right) ^{k^2/4-k} < 2^{-\Omega (k^2)} = 2^{-\Omega ((\alpha n)^2)}. \end{aligned}$$

The claim now follows by taking a union bound over at most *n* levels. All conditions of Theorem 27 are satisfied and the corollary follows. $\square$

## Unrestricted Black-Box Algorithms

Intuitively, one would expect that Slo$^\alpha _{\varepsilon ,r}$ becomes hard (contains instances that require exponential runtime) when the fitness valleys are less dense than the deceptive regions. The following theorem confirms this intuition and shows that for any constants $\alpha \in (0,1)$ and $\varepsilon \in (\alpha ,1)$ the black-box complexity of Slo$^\alpha _{\varepsilon ,r}$ is exponential even for unrestricted black-box algorithms.

### Theorem 29

If $\delta =\delta (n)\in (0,1)$, $\alpha =\alpha (n)\in (0,1-\delta -3/n)$ then the unrestricted black-box complexity of $\textsc{Slo}^{\alpha }_{\alpha +\delta +3/n, n-1}$ is at least $2^{n\delta /2-1}$.

### Proof

By Lemma 6, it suffices to prove the statement for the parameter $r_{\max }=\lfloor n/2\rfloor$.

We first define a class $\mathcal {I}$ of the so-called $f_{z,\alpha ,\delta }$ functions, i.e., one for each possible hidden sub-string *z* of length $m:=\lceil n \delta /2\rceil$, and show that the unrestricted black-box complexity of this class is $2^{n\delta /2-1}$. Then we show that every $f_{z,\alpha ,\delta }$ belongs to $\textsc{Slo}^{\alpha }_{\alpha +\delta ,\lfloor n/2\rfloor }$, it then follows from Lemma 3 that the claim is implied.

Define $\ell :=\lfloor (1-\alpha )n\rfloor -2m$, and note that $\ell > ((\delta +3/n)n - 1)$ $- 2(n\delta /2 + 1) = 0$ since $\alpha <1-\delta -3/n$. To simplify notation, we divide any bit string $x\in \{0,1\}^n$ into three sub-strings $x=(a,b,c)$, where

$$\begin{aligned} a&:= x_1\cdots x_\ell ,\\ b&:= x_{\ell +1}\cdots x_{\ell +m},\\ c&:= x_{\ell +m+1}\cdots x_n. \end{aligned}$$

For any bit string $x=(a,b,c)\in \{0,1\}^n$ and $z\in \{0,1\}^m$, define

$$\begin{aligned} f_{z,\alpha ,\delta }(a,b,c) := {\left\{ \begin{array}{ll} \sum _{i=1}^{\ell } a_i & \hbox{if}\quad a \ne 1^\ell ,\\ \ell +2m & \hbox{if}\quad a=1^\ell\quad \mathrm{and}\quad b\ne z,\\ \ell +m + \sum _{i=\ell +m+1}^n c_i & \hbox{if}\quad a = 1^\ell \quad\mathrm{and}\quad b=z. \end{array}\right. } \end{aligned}$$

The global optimum of $f_{z,\alpha ,\delta }$ is the bit string $x^*:=1^\ell z 1^{n-\ell -m}$. This problem is hard for all black-box algorithms because to find $x^*$ the algorithm needs to guess correctly the *m* bits of the hidden bit string *z*, from position $\ell$ to position $\ell +m$.

Following the same arguments as in the proof of Theorem 1 in [20], we apply Yao’s minimax principle to show that the worst case expected time for any black-box algorithm to discover the hidden bit string is $(2^{m}+1)/2$. Let $\mathcal {I}:=\{ f_{z,\alpha ,\delta } \mid z\in \{0,1\}^{m}\}$ be the set of instances, and *p* be the uniform distribution on $\mathcal {I}$. We consider the first point in time that the algorithm queries a search point $x=(a,b,c)$ with $b=z$, clearly this is a lower bound on the number of time steps to find $x^*$.

For all $a\in \{0,1\}^\ell , b,b'\in \{0,1\}^m {\setminus} \{z\}$ and $c\in \{0,1\}^{n-m-\ell }$, it holds that $f_{z,\alpha ,\delta }(abc)=f_{z,\alpha ,\delta }(ab'c)$. Hence, as long as a deterministic black-box algorithm has not queried a search point $x=(a,b,c)$ with $b=z$ yet, the values obtained by querying $f_{z,\alpha ,\delta }(x)$ are independent of *z*. Therefore, after querying *t* search points without discovering *z*, there are still at least $2^m-t$ sub-strings that can be *z* with equal probabilities. The expected number of queries until a *x* with $b=z$ is queried for the first time is therefore at least $(2^m+1)/2\ge 2^{n\delta /2-1}$ for any black-box algorithm.

It remains to prove that $f_{z,\alpha ,\delta }$ belongs to Slo$^{\alpha }_{\alpha +\delta }$. We partition the search space into $n+1$ levels defined by

$$\begin{aligned} A_j := {\left\{ \begin{array}{ll} \{(a,b,c) \mid \sum _{i=1}^\ell a_i=j\} & \mathrm{for}\quad 0\le j< \ell ,\\ \{(1^\ell ,b,c) \mid \sum _{i=1}^{m}[b_i=z_i]=j-\ell \} & \mathrm{for}\quad\ell \le j<\ell +m,\\ \{(1^\ell ,z,c) \mid \sum _{i=1}^{n-\ell -m} c_i=j-\ell -m\} & \mathrm{for}\quad\ell +m\le j\le n.\\ \end{array}\right. } \end{aligned}$$

Trivially, the only optimal search point with respect to $f_{z,\alpha ,\delta }$ belongs to $A_n$, and for any level $A_j$, one can obtain a bit string in level $A_{j+1}$ by flipping a single bit.

The deceptive pairs are $(A_{\ell +i},A_{\ell +j})$ for $0\le i <m$ and $i<j\le 2m$. Note that all bit strings in $B:=\cup _{j=\ell }^{\ell +2m}A_j$ agree on the first $\ell$ bit-positions, and $\ell >(1-\alpha -\delta )n-3$, hence by Lemma 15, *B* is $(1-\ell /n,\lfloor n/2\rfloor )$-sparse, and therefore $(\alpha +\delta +3/n,\lfloor n/2\rfloor )$-sparse. Furthermore, for any bit string in $A_{\ell +j}$ where $j\le 2m$, flipping any of the last $n-\ell -2m \ge \alpha n$ bits in *x* which differs from the global optimum $x^*$ will lead to a search point in $A_{\ge \ell +j}$. Therefore, $A_{\ell +j}$ is $\alpha$-dense with respect to $A_{\ge \ell +j}$. $\square$

The following corollary expresses the theorem above in a more natural form.

### Corollary 30

For any $\alpha \in (0,1-3/n)$ and $\varepsilon \in (\alpha +3/n,1)$, the unrestricted black-box complexity of $\textsc{Slo}^{\alpha }_{\varepsilon , n-1}$ is at least $2^{\frac{n(\varepsilon -\alpha )-5}{2}}$.

### Proof

Define $\delta :=\varepsilon -\alpha -3/n$, which gives $\alpha =\varepsilon -\delta -3/n$. The assumption $\varepsilon <1$ implies that $\alpha <1-\delta -3/n$. Furthermore, $\varepsilon =\alpha +\delta +3/n$ and $n\delta /2-1=\frac{n(\varepsilon -\alpha )-5}{2}$. The result now follows from Theorem 29. $\square$

## Elitist Black-Box Algorithms

Elitist black-box algorithms are those that always memorise a certain number of the best solutions they have discovered so far. Particularly they only generate new solutions to evaluate next by modifying or recombining these best solutions. The elitist black-box model of [18] captures all these algorithms.

The elitist black-box model is described in Algorithm 3. The initial $\mu$ search points may be sampled adaptively, i.e., the *i*-th sample may depend on the ranking of the first $i-1$ samples w.r. t.  *f*. In each of the main iterations, this algorithm samples $\lambda$ new search points from distributions that depend only on the current population $P_t$ and the ranking of it. In each of these iterations, the $\lambda$ offspring do not need to be independent of each other. However, it is required that all of the $\lambda$ offspring are created before the fitness of any of them is evaluated.

In the elitist ($\mu +\lambda$) EA (which is a special case of Algorithm 3), a new offspring population *P* is created by selecting parents uniformly from $P_t$ and perturbing them with $p_{\mathrm{mut}}$. The surviving population $P_{t+1}$ of the next generation then composes of the $\mu$ best individuals among both parent and offspring populations $P_t \cup P$.

**Algorithm 3:**
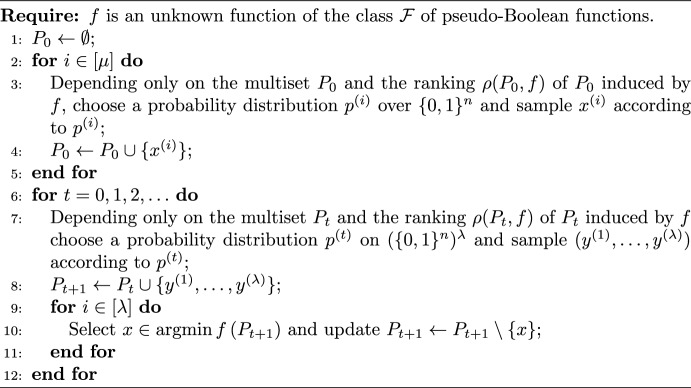
$(\mu +\lambda )$ elitist black-box algorithm [18]

To prove that Slo$^\alpha _{\varepsilon ,r}$ has exponential elitist black-box complexity, we consider a sub-class BBFunnel (see Definition 31 and Theorem 39). The sub-class captures the same features of the problem Funnel introduced in [12], however it is a broader class.

**Fig. 3:**
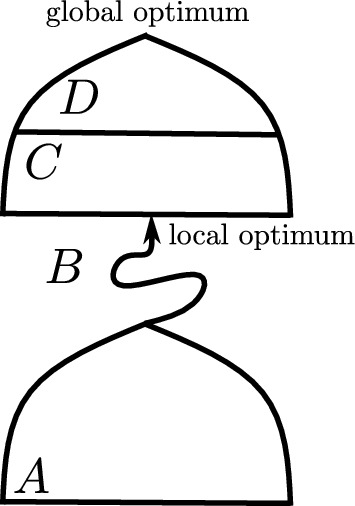
Illustration of the four main regions of BBFunnel.

Figure 3 illustrates the intuition behind BBFunnel. The search space is divided into different regions (see Definition 31). Region *A* comprises a large part of the search space such that with high probability, a randomly sampled search point belongs to *A*. The fitness increases in region *A* towards region *B*. Region *B* is a sparse random path which ends in a local optimum. Region *C* is a dense “valley” with fitness lower than the local optimum. Finally, region *D* leads to the global optimum. The design of the function class ensures that elitist black-box algorithms either get trapped onto region *B*, or must wait for a long jump from region *A* to region $C\cup D$. In contrast, a properly configured non-elitist EA will allow sub-populations to fall off the local optimum into the dense valley region *C* and eventually reach the fitter region *D*.

We now give a more detailed description of the function class. It is partly defined in terms of the functions $\textsc{Lo} _z$ and $\textsc{Om}$ (see Sect. 2). In what follows, by a simple path we mean a sequence of binary strings with no repetitions and same number of bits, where each element differs in exactly one bit from the previous element. Formally, a *simple path* is any sequence of bit strings $x_0,x_1,\ldots , x_\ell \in \{0,1\}^m$, where for all $i,j\in [\ell ]_0$, if $x_i=x_j$ then $i=j$ (i.e., uniqueness of each string), and for every $i\in [\ell ]$, $\mathrm{H}(x_{i-1},x_{i})=1$ (i.e., consecutively separated by a Hamming distance of 1) [20]. The string $x_0$ is referred to as the starting point of the path, and $\ell$ is called the length of the path, i.e., the number on consecutive pairs $(x_{i-1},x_i)$. Clearly, simple paths contain no cycles.

### Definition 31

For any integers $$1\le u< v < w\le n$$, and a simple path $$p_0,p_1,\ldots , p_\ell$$$$\in \{0,1\}^{v-u}$$ of length $$\ell \in {{\mathrm{poly}}}(n)$$ starting from $$p_0:=0^{v-u}$$, let

$$\begin{aligned} {\textsc{BBFunnel}} _\ell (x) := \left\{ \begin{array}{lll} {\textsc{Lo}}_y(x)+\ell & {\mathrm{if}}\quad w<{\textsc{Lo}}_y(x)\le n & (D) \\ {\textsc{Lo}} _y(x) & {\mathrm{if}}\quad v < {\textsc{Lo}}_y(x) \le w & (C) \\ i+w & {\mathrm{if}}\quad x=1^up_i0^{n-v}\,\, {\mathrm{where}}\,\, i\in [\ell ] & (B) \\ -n-{\textsc{Om}} (x) & {\mathrm{if}}\quad {\textsc{Lo}}_y(x)\ge u\,\,{\mathrm{and}}\,\, x\not \in B\cup C\cup D & (B')\\ {\textsc{Lo}}_y(x) & {\mathrm{if}}\quad {\textsc{Om}} (x)\le u & (A)\\ -{\textsc{Om}} (x) & {\mathrm{if}}\quad x\not \in A\cup B'\cup B\cup C\cup D & (A') \end{array} \right. \end{aligned}$$

where $$y:=1^u\cdot p_\ell \cdot z\cdot 1^{n-w}$$ and a bit string $$z\in \{0,1\}^{w-v}.$$ The labels $$A,A',$$$$B,B',C,D$$ used in the case separation for *x* are also the names of the sets of strings *x* satisfying the respective cases, for example $$D:=\{x\in \{0,1\}^n\mid w< \textsc{Lo} _y(x) \le n\}$$.

To show a worst-case runtime for any randomised elitist black-box algorithm, we will apply Yao’s minimax principle. We fix $\mathcal {A}$ to be the set of deterministic algorithms, which may become a realisation of the randomised elitist $(\mu +\lambda )$ black-box algorithms (see Algorithm 3). As pointed out by Carola Doerr and Lengler, to account for the possibility that a deterministic elitist black-box algorithm can enter an infinite loop, it is necessary to extend $\mathcal {A}$ to a bigger class of algorithms by assisting them with some additional information [18]. Clearly, lower bounds that hold in this more general class, also hold for the original class. The extra information we give to the algorithms will be later detailed in our proof.

We define a distribution *p* to sample a random instance $I_p$ from the class BBFunnel as follows. The bit string *z* is chosen uniformly at random. Furthermore, the simple path is constructed randomly using the following two definitions (see [20]).

### Definition 32

A sequence $R=R(b)$ of points $r_0,\dots ,r_b$ in $\{0,1\}^m$ where for all $i<b$, $r_{i+1}$ equals $r_i,$ except for one bit-position chosen uniformly at random, is an *m*-dimensional random path of length *b*.

We will assume that $b=n^{2\delta }\mu$ for two constants $\mu >0$ and $\delta \in (0,1),$ and $m:=v-u$ in what follows. The sequence *R* can contain cycles, which may be excluded as follows.

### Definition 33

The random simple path *P* based on *R* is a subsequence of *R*,  defined so that $p_0:=r_0$, and for $i\ge 0$, if $p_i=r_j$, then $p_{i+1}:=r_{k+1}$ where $k\ge j$ is the largest index where $r_j=r_k$. Formally, we have the recurrence

$$\begin{aligned} p_i := {\left\{ \begin{array}{ll} r_i & \hbox{if}\quad i=0,\\ r_{\max \{k\mid r_k=r_{i-1}\} + 1} & \hbox{if}\quad i\ge 1. \end{array}\right. } \end{aligned}$$

This construction ensures that $P=(p_0,\ldots ,p_\ell )$ is a simple path, where $\ell$ is the random length of *P*. In Lemma 35 below, we will show that with probability $1-2^{-\Omega (n^\delta )},$ the random path *R* has no cycles longer than $n^\delta$. Since *R* has length $b=n^{2\delta }\mu$, removing cycles of length at most $n^\delta$ from *R* leads to a path *P* with length $\ell \ge n^{\delta }\mu$.

We need the following drift analysis result for the next lemma.

### Theorem 34

(Hajek’s drift theorem, Thm. 2.3 (2.8) [24]). Let $(Y_t)_{t\ge 0}$ be a sequence of random variables on a probability space $(\Omega ,\mathcal {F},P)$ adapted to an increasing sequence $(\mathcal {F}_{t})_{t\ge 0}$ of sub-$\sigma$-algebras of $\mathcal {F}$ where for $a<b$ it holds

1. $$E_{1,t}:=E\left[ e^{\eta (Y_{t+1}-Y_t)};Y_t>a\mid \mathcal {F}_{t}\right] \le \rho $$, and
2. $$E_{2,t}:=E\left[ e^{\eta (Y_{t+1}-a)};Y_t\le a\mid \mathcal {F}_{t}\right] \le D$$,

then

$$\begin{aligned} \Pr \left( Y_t\ge b\mid \mathcal {F}_{0}\right) \le \rho ^te^{\eta (Y_0-b)}+\frac{1-\rho ^t}{1-\rho }De^{\eta (a-b)}. \end{aligned}$$

### Lemma 35

Let $r_0,\ldots , r_b,$ be an *m*-dimensional random path. For any $d\le m/5$, any bit string $z\in \{0,1\}^m$, any $i\in [0..b-1]$ and any $t\in [1..b-i]$, and any $c\le \log (8/7)$

(i) $$ \Pr \left( H(z,r_{i+t})\le d\mid H(z,r_{i})\right) \le 2^{d-H(z,r_{i})-ct}+e^{-\Omega (m)},$$
(ii) $$\Pr \left( H(z,r_{i+t})\le d\right) \le 2^{d-ct}+e^{-\Omega (m)}$$.

### Proof

We apply Theorem 34 (Hajek’s drift theorem) to the process $Y_t:= m-H(z,r_{i+t})$ for the filtration $\mathcal {F}_{t}:=\sigma (\{H(z,r_{i+j})\mid j\in [0..t]\})$, and the parameters $a:=(3/4)m$, $b:=m-d\ge a+m/20$, and $\eta :=\ln (2)$.

For $Y_t> a,$ we have $\Pr \left( Y_{t+1}-Y_t=1\right) \le 1/4$ since the random walk must flip one of at most *m*/4 bits that differ from *z* in $r_t$ to decrease the Hamming distance to *z*. Thus

$$\begin{aligned} E_{1,t}&\le \frac{1}{4} e^{\eta }+\frac{3}{4} e^{-\eta } = \frac{7}{8}=:\rho . \end{aligned}$$

For $Y_t\le a$, we have $Y_{t+1}\le a+1$, and $E_{2,t} \le e^{\eta } = 2 =: D$. Thus, both conditions of Hajek’s drift theorem are satisfied and given that *D*, $\eta$, *t* and $\rho$ are constant, we get

$$\begin{aligned} \Pr \left( H(z,r_{i+t})\le d\mid H(z,r_i)\right)&= \Pr \left( Y_t\ge b\mid \mathcal {F}_{0}\right) \\&\le \rho ^{t}e^{\eta (Y_0-b)}+e^{-\Omega (b-a)} \\&= (2^{\log (\rho )})^{t} \cdot (2)^{(m - H(z,r_{i}) - b)} + e^{-\Omega (m)}\\&= 2^{d-H(z,r_{i})+t\log (\rho )}+e^{-\Omega (m)}, \end{aligned}$$

and part (i) of the result follows since $\log (\rho )=-\log (8/7)\le -c$.

Finally, for part (ii) we have an unconditional bound

$$\begin{aligned} \Pr \left( H(z,r_{i+t})\le d\right)&= E\left[ 1\!\!1_{\left\{ H(z,r_{i+t})\le d\right\} }\right] \\&= E\left[ E\left[ 1\!\!1_{\left\{ H(z,r_{i+t})\le d\right\} }\mid H(z,r_i)\right] \right] \\&= E\left[ \Pr \left( H(z,r_{i+t})\le d\mid H(z,r_i)\right) \right] \\&\le E\left[ 2^{d-H(z,r_{i})-ct}\right] +e^{-\Omega (m)}\\&\le 2^{d-ct}+e^{-\Omega (m)}. \end{aligned}$$

$$\square $$

Lemma 35 is a variant of Lemma 1 from [20], but uses a different proof idea and provides more flexibility with the choice of *z* in part (i), which is required in what follows. A first simple application is to show that it is easy to build long simple paths based on *m*-dimensional random paths.

### Lemma 36

Let $n\in \mathbb {N}$, and $p_0,\ldots ,p_{\ell }$ be a simple path based on an *m*-dimensional random path $r_0,\ldots ,r_b$ where $m=\Omega (n)$. Then for any constant $\delta <1$ and any positive integer $\mu \in {{\mathrm{poly}}}(n)$, if $b\ge n^{2\delta }\mu$ and $b\in {{\mathrm{poly}}}(n)$ then $\Pr \left( \ell <n^{\delta } \mu \right) \le 2^{-\Omega (n^\delta )}$.

### Proof

For any fixed *i*, define events $E_i:= \{\max \{k\mid r_k = r_i\} \ge i + n^{\delta }\}$ and $E:= \bigcup _{i\in [0,b]} E_i$. By a union bound, we have

$$\begin{aligned} \Pr \left( E_i\right)&\le \sum _{j:j\ge i+n^{\delta }}\Pr \left( H(r_i,r_j)<1\right) , \end{aligned}$$

and the worst case is at $i=0$, so this is

$$\begin{aligned}&\le \sum _{j:j\ge n^{\delta }}\Pr \left( H(r_0,r_{\lfloor n^\delta \rfloor })\le 1\right) \le b \Pr \left( H(r_0,r_{\lfloor n^\delta \rfloor })\le 1\right) . \end{aligned}$$

Since $b={{\mathrm{poly}}}(n)$, then by Lemma 35, there exists a constant *c* such that

$$\begin{aligned} \Pr \left( E\right)&\le (b+1)b \Pr \left( H(r_0,r_{\lfloor n^\delta \rfloor })\le 1\right) \le n^{O(1)} \left( 2^{1 - c \lfloor n^\delta \rfloor } + e^{-\Omega (m)}\right) \end{aligned}$$

and given $m=\Omega (n)$, this is

$$\begin{aligned}&\le 2^{-\Omega (n^{\delta })} + 2^{-\Omega (n)} = 2^{-\Omega (n^{\delta })}. \end{aligned}$$

Therefore, with probability at least $1- 2^{-\Omega (n^{\delta })}$ the complement event $\lnot E$ occurs, and this is the event that no cycles of length at least $n^{\delta }$ are generated. Assume that $\lnot E$ and the simple path generated is $p_0,p_1,\ldots ,p_{\ell }$ which corresponds to points $r_{k_0},r_{k_1},\ldots ,r_{k_{\ell }}$ of the random path. Then, we have $(k_i - 1) - k_{i-1}< n^{\delta }$ or equivalently $k_i - k_{i-1}\le n^{\delta }$ for any $i\in [\ell ]$. Note that $k_0=0$ and $k_{\ell }=b$, so

$$\begin{aligned} b = k_{\ell } \le k_{\ell -1} + n^{\delta } \le k_{\ell -2} + 2n^{\delta } \le \cdots \le k_{0} + \ell n^{\delta } = \ell n^{\delta } \end{aligned}$$

which implies $\ell \ge b/n^{\delta } \ge \mu n^{2\delta }/n^{\delta } = \mu n^{\delta }$. Thus $\Pr \left( \ell \ge \mu n^{\delta }\right) \ge 1-2^{-\Omega (n^{\delta })}$. $\square$

The following lemmas allow estimating the sparsity of simple paths based on *m*-dimensional random paths.

### Lemma 37

Let $n\in \mathbb {N}$, and $r_0,\ldots , r_b$ be an *m*-dimensional random path with $m=\omega (\log (n))$ and $b\le n^k$ for a positive constant $k<m/10$ independent of *n*, define $u:=(1/c)(\log (b)+2k+5)$ where $c=\log (8/7)$. Then with probability at least 0.74, for all $d\ge u$ and $i\in [b-d]$, $H(r_i,r_{i+d})>2k$ for all sufficiently large *n*.

### Proof

Define events $\mathcal {E}_{i,d}:=\{H(r_i,r_{i+d})\le 2k\}$. By Lemma 35, part (ii) and a union bound,

$$\begin{aligned} \Pr \left( \cup _{i=1}^{b-u}\cup _{d=u}^{b-i}\mathcal {E}_{i,d}\right)&\le \sum _{i=1}^{b-u}\sum _{d=u}^{b-i}\Pr \left( \mathcal {E}_{i,d}\right) \\&\le \sum _{i=1}^{b-u}\sum _{d=u}^{b-i}\left( 2^{2k-cd}+e^{-\Omega (m)}\right) \\&< b\left( \sum _{v=0}^\infty 2^{2k-c(u+v)}\right) +b^2e^{-\Omega (m)}\\&= b \cdot 2^{-\log {b}-5}\left( \sum _{v=0}^\infty 2^{-cv}\right) + n^{O(1)}e^{-\Omega (m)}\\&= 2^{-5} \cdot \frac{1}{1-2^{-c}} + e^{-\Omega (m)} = \frac{1}{4} + o(1) < \frac{26}{100}, \end{aligned}$$

where the last inequality holds for all sufficiently large *n*. $\square$

### Lemma 38

Let $P=(p_0,\cdots ,p_\ell )$ be a simple random path based on an *m*-dimensional random path *R*, with $m=\omega (\log {n})$, of length $b\le n^k$ for a constant $k<m/10$ independent of *n*, and *v* be any integer with $m<v<n$. Then, for $\varepsilon := 21k\log (n)/n$, and sufficiently large *n*, the set $B'=\{1^{v-m} p_i0^{n-v}\mid i\in [\ell ]_0\}$ is $(\varepsilon ,\lfloor n/2\rfloor )$-sparse with probability at least 0.74.

### Proof

Let $u:=(1/c)(\log (b)+2k+5)$ where $c=\log (8/7)$ and note that for sufficiently large *n* we have

18 $$\begin{aligned} \frac{4u}{n} \le \frac{4}{c}\cdot \frac{\log (n^k) + 2k + 5}{n} = \frac{4}{c}\cdot \frac{k \log {n}}{n} + o(1) \le \varepsilon . \end{aligned}$$

We assume that for all $d\ge u$ and $i\in [b-d]$, $H(r_i,r_{i+d})>2k$, otherwise we consider the random path a *failure*. By Lemma 37, the probability of a failure is at most $1-0.74$. Conditioned on the event that path is not a failure, we now show that the set $B'$ is $(\varepsilon ,\lfloor n/2\rfloor )$-sparse and this proves the lemma.

By Lemma 12(3), for any sets $B'\subseteq B\subseteq \{0,1\}^n$, if *B* is $\varepsilon$-sparse, then the subset $B'$ is also $\varepsilon$-sparse. By construction, each simple path element $p_i$ equals some element $r_j$ on the random path $R=(r_0,\ldots ,r_b)$. It therefore suffices to prove the $\varepsilon$-sparsity of the set $B:=\{1^{v-m}r_i0^{n-v}\mid i\in [0..b]\}\supseteq B'$, where by assumption $b\le n^k$.

We first verify the conditions of Definition 5 for the case $x\in B$. By definition of *B*, there must exist a $j\in [0..b]$ such that $x=1^{v-m} r_j0^{n-v}$. For an arbitrary $r\in [1, n/2]$, define

$$\begin{aligned} X_j&:= \left| \left\{ y\in R\mid H(r_j,y)=r\right\} \right| . \end{aligned}$$

We will prove that unless a failure has occurred, it holds for all $j\in [0..b]$ that

19 $$\begin{aligned} X_j \le \varepsilon {n\atopwithdelims ()r}. \end{aligned}$$

Note that this is equivalent to inequality (1) when $x\in B$, because

$$\begin{aligned} X_j&= \left| \left\{ y\in R\mid H(r_j,y)=r\right\} \right| \\ &= \left| \left\{ 1^{v-m} y0^{n-v}\in B\mid H(1^{v-m} r_j0^{n-v},1^{v-m} y0^{n-v})=r\right\} \right| \\ &= \left| S_r(x)\cap B\right| . \end{aligned}$$

For the case where $k+1\le r\le n/2$, since *k* is a constant, it holds

20 $$\begin{aligned} \varepsilon {n\atopwithdelims ()r}&\ge \varepsilon {n\atopwithdelims ()k+1} \ge \varepsilon \left( \frac{n}{k+1}\right) ^{k+1} = n^k \left( \frac{\varepsilon n}{(k+1)^{k+1}}\right) \nonumber \\&= n^k\frac{21k\log (n)}{(k+1)^{k+1}} = n^k\cdot \Omega (\log {n}) > |B|, \end{aligned}$$

for *n* sufficiently large. So in this case, Eq. (19) holds for all $j\in [b]$.

We now consider the case $1\le r\le k$. Unless the random path is a failure, note that for any $r_j \in R$ we have that $H(r_j,r_{j+d})>2k\ge 2r > r$ and also that $H(r_{j-d},r_{j})>2k\ge 2r > r$ for all $d\ge u$. Thus only the remaining elements in *R*, which are $\{r_{j-u+1},\ldots ,r_{j-1},r_{j+1},\ldots ,r_{j+u-1}\}$ (assuming their indices are valid in [0, *b*]) can have Hamming distance *r* to $r_j$, and this is at most $2(u-1)<2u$ elements. Therefore, (18) implies that for $x=1^{v-m} r_j 0^{n-v}\in B,$

$$\begin{aligned} X_j = |S_r(x)\cap B|< 2u \le \varepsilon n/2 < \varepsilon {n\atopwithdelims ()r}. \end{aligned}$$

This proves that (1) holds when $x\in B$.

We now consider any other case where $x\not \in B$, where we assume that $r\le n/2$. We will prove that if the random path is not a failure, then

21 $$\begin{aligned} Y&:= \left| \left\{ 1^{v-m} y 0^{n-v}\in B\mid H(x,1^{v-m} y 0^{n-v})=r\right\} \right| \\ &= \left| \left\{ y\in R\mid H(x,1^u y 0^{n-v})=r\right\} \right| <\varepsilon {n\atopwithdelims ()r}. \end{aligned}$$

In the case of $k\le r\le n/2$, analogously to (20), for sufficiently large *n* we get

$$\begin{aligned} \varepsilon {n\atopwithdelims ()r} \ge n^k\frac{21k\log (n)}{(k+1)^{k+1}} = n^{k} \cdot \Omega (\log {n}) > 2|B|\ge 2Y, \end{aligned}$$

so Eq. (21) is satisfied.

In the other case, assume that $1\le r\le k$, and that $1^{v-m} r_j 0^{n-v}\in B$ is one of the elements in *B* with minimal distance $d_0 = H(1^{v-m} r_j 0^{n-v},x)$ to *x*. If $d_0\ge r+1$, then there is nothing to prove since $Y=0$. Hence, we consider the case $d_0\le r\le k$. For any element $1^{v-m} r_i 0^{n-v}\in B$, by the triangle inequality

$$\begin{aligned} H(x,1^{v-m} r_i 0^{n-v})\ge H(1^{v-m} r_j 0^{n-v}, 1^{v-m} r_i 0^{n-v})-d_0. \end{aligned}$$

So if $H(1^{v-m} r_j 0^{n-v}, 1^{v-m} r_i 0^{n-v})>d_0+r$ then $H(x,1^{v-m} r_i 0^{n-v})>r$ and the element $1^{v-m} r_i 0^{n-v}$ is definitely not counted in *Y*. In other words, *Y* is at most the number of elements in *B* in Hamming distance at most $d_0+r$ to $1^{v-m} r_j 0^{n-v}$. For our case $d_0+r \le 2k$, then analogously to a previous argument, if the random path is not a failure then *Y* is at most the cardinality of the set $\{r_{j-u+1},\ldots ,r_{j+u-1}\}$. It follows by (18) that

$$\begin{aligned} Y \le 2(u-1) + 1< 2u \le \varepsilon n/2< \varepsilon {n\atopwithdelims ()r}. \end{aligned}$$

We have proved that inequality (1) holds for all *x* and $r\in [\lfloor n/2 \rfloor ]$. $\square$

Lemma 35 can be used to prove that BBFunnel belongs to the class Slo$^\alpha _{\varepsilon ,r}$.

### Theorem 39

For any positive constant $k\le (v-u)/10$, any $\varepsilon \ge 21k\log (n)/n$, and for sufficiently large *n*, with probability at least 74/100, a function sampled from the class BBFunnel with $w\in \Theta (n)$, $v-u=\omega (\log {n})$, and $b\le n^k$ according to the distribution *p* belongs to the problem class $\textsc{Slo}^{1-w/n}_{\varepsilon ,n-1}$.

### Proof

By Lemma 6, it suffices to prove the lemma for $r_{\max }=\lfloor n/2\rfloor$. Define $\sigma :=1-74/100$. We show that an instance of BBFunnel sampled according to distribution *p* satisfies the criteria of Definition 8 with probability at least $1-\sigma$. Define sets^1^

$$\begin{array}{ll} {\tilde{B}}'_i:= \{ x\in B' \mid \textsc{Om} (x)=n-i+1 \}&\mathrm{for}\quad 1\le i \le n-u+1,\\ {\tilde{A}}'_i:= \{ x\in A' \mid \textsc{Om} (x)=n-i \}&\mathrm{for}\quad 1\le i \le n-u-1,\\ {\tilde{A}}_i:= \{ x\in A \mid \textsc{Lo} _y(x)=i-1 \}&\mathrm{for}\quad 1\le i \le u+1,\\ {\tilde{B}}_i:= \{ 1^up_{i}0^{n-v} \}&\mathrm{for}\quad 1\le i \le \ell ,\\ {\tilde{C}}_i:= \{ x\in C\mid \textsc{Lo} _y(x)=v+i \}&\mathrm{for}\quad 1\le i \le w-v,\\ {\tilde{D}}_i:= \{ x\in D\mid \textsc{Lo} _y(x)=w+i \}&\mathrm{for}\quad 1\le i \le n-w. \end{array}$$

Some of the sets ${\tilde{A}}'_i$ and ${\tilde{B}}'_i$ can be empty, and in the following partition we skip their inclusion. We define the partition of $\{0,1\}^n$ as

$$\begin{aligned} (A_1,\ldots , A_m) := (&{\tilde{B}}'_1, {\tilde{B}}'_2,\ldots , {\tilde{A}}'_1, {\tilde{A}}'_2,\ldots ,\\&{\tilde{A}}_1, {\tilde{A}}_2,\ldots , {\tilde{B}}_1, {\tilde{B}}_2,\ldots ,\\&{\tilde{C}}_1, {\tilde{C}}_2,\ldots , {\tilde{D}}_1, {\tilde{D}}_2,\ldots ,{\tilde{D}}_{n-w}), \end{aligned}$$

with the number of levels *m* of at most $2(n-u) + (u + 1) + \ell+ (w-v)$ $+ (n-w) = 3n - u - v + \ell + 1 \in {{\mathrm{poly}}}(n)$ as $\ell \in {{\mathrm{poly}}}(n)$.

It is easy to see that for for all $x\in A_j$ where $A_j$ comes from an ${\tilde{A}}_i$, ${\tilde{B}}_i$, ${\tilde{C}}_i$, or ${\tilde{D}}_i$ set, there exists an element $y\in A_{\ge j+1}$ with $H(x,y)=\mathord {\text {O}}\mathord {\left( 1\right) }$. If $A_j$ comes from a ${\tilde{B}}'_i$, note that $\textsc{Lo} _y(x)\ge u$, thus flipping the first bit of *x* from 1 to 0 creates a search point in $A' \cup A$, in other words at a higher level. If $A_j$ comes from an ${\tilde{A}}'_i$, note that $\textsc{Lo} _y(x)\le u-1$ because otherwise *x* is in $B'$, in other words, there are at most $u-1$ ones in the first *u* bits of *x*. In addition, $\textsc{Om} (x)\ge u+1$ because otherwise *x* is in *A*, thus there are at least $u+1 - (u-1)=2$ ones in the last $n-u$ bits of *x* and flipping any of these two bits leads to a search point either in $A'_{\ge i+1}$ or in *A*. These show that there always exists $y\in A_{\ge j+1}$ with Hamming distance *O*(1) for any $x\in A_j$.

The only deceptive pairs of the partition are $({\tilde{B}}_i,{\tilde{C}}_j)$ for all *i*, *j*. Application of Lemma 38 with $m:=u-v=\omega (\log {n})$ and the rest of the parameters as assumed by the conditions of the theorem implies that the set $B:=\cup _i {\tilde{B}}_i$ is $(\varepsilon ,\lfloor n/2 \rfloor )$-sparse with probability $1-\sigma$. Furthermore, every ${\tilde{C}}_{j}$ is a $(1-w/n)$-dense set with respect to ${\tilde{C}}_{\ge j}\cup D$. This is because any $x\in C_{j}$ has $w\ge \textsc{Lo} _y(x)\ge j+v$ so flipping any bit of the last $n-w$ bits of *x* produces a point in ${\tilde{C}}_{\ge j}\cup D$. All conditions of Definition 8 are satisfied therefore BBFunnel belongs to $\textsc{Slo}^{1-w/n}_{\varepsilon ,\lfloor n/2\rfloor }$. $\square$

We notice the following property of $\textsc{BBFunnel}$. Regardless of how the hidden string *z* and the points $p_1,\ldots ,p_{\ell }$ on the path are chosen, for all search points $x\in A$, i.e., those with no more than *u* 1s, the value of $\textsc{BBFunnel} _{\ell }(x)$ is always equal the number of leading ones in the first *u* bits of *x*. In other words, all instances of $\textsc{BBFunnel}$ define the fitness values in region *A* identically. Any black-box algorithm that knows it is optimising an instance of $\textsc{BBFunnel}$ immediately knows (i.e., without making any queries) the fitness of all the search points in *A*. These points include the point $1^u 0^{n-u}$ which contains the starting point $p_0=0^{v-u}$ of the path.

In order to reach the optimum $y=1^u\cdot p_\ell \cdot z\cdot 1^{n-w}$, the algorithm must discover the end point $p_\ell$ of the simple random path and the hidden sub-string *z*. The following lemma, which holds for any elitist black-box algorithm with a polynomial number of queries, shows that unless the algorithm explores large parts of the path, it will take exponential time to discover the end point $p_\ell$.

### Lemma 40

For all $t\in \mathbb {N}$, let $M_t$ be the set of points queried up to step *t* of algorithm $\textbf{A}$ (unrestricted black box algorithm) with $\mu \in {{\mathrm{poly}}}(n)$. Assume that $d(n)\le m/5$. Then there exists a constant $c>0$ such that if the simple path $p_0,p_1,\ldots ,p_\ell$ is sampled from the distribution *p* corresponding to an instance of BBFunnel with $m=\omega (\log {n})$, and the algorithm after step $t\le e^{cd(n)}$ satisfies $M_t\cap \{p_i,\ldots ,p_\ell \}=\emptyset$ and queries point *q* in iteration $t+1$, then $\Pr \left( q=p_{i+d(n)}\right) =e^{-\Omega (d(n))}$.

### Proof

We aim to apply Lemma 35, and therefore must relate the simple path $p_0,$ $p_1,$ $\ldots ,p_\ell$ with the random walk $r_0,r_1,\ldots ,r_b$, where $p_0=r_0$ and $b:=n^{2\delta }\mu \in {{\mathrm{poly}}}(n)$ for some constant $\delta >0$. Since every simple path element $p_i$ is on the random walk $r_0,r_1,\ldots ,r_b$, there must exist two natural numbers *j* with $i\le j\le b$ and $d'$ with $d(n)\le d'\le b-j$ such that $p_i = r_j$ and $p_{i+d(n)}=r_{j+d'}$. We apply the principles of deferred decisions where the random path elements $r_{j+d(n)}, \ldots , r_{b}$ are revealed (or equivalently sampled) after the algorithm have queried point *q*.

For any $z\in M_{t+1}$, we therefore have

$$\begin{aligned} \Pr \left( q=p_{i+d(n)}\right)&= \Pr \left( H(z,p_{i+d(n)}) = 0\right) \\&= \Pr \left( H(z,r_{j+d'})=0\right) \\&\le \sum _{k=d(n)}^{b-j}\Pr \left( H(z,r_{j+k})=0\right) , \end{aligned}$$

by Lemma 35 (2) with $d=0$, and some constants $c_1,c_2>0$, this is

$$\begin{aligned}&\le \sum _{k=d(n)}^{b-j}\left( 2^{-c_1k}+e^{-c_2m}\right) \\&\le \left( 2^{-c_1d(n)}\sum _{k=0}^{\infty }2^{-c_1k}\right) +be^{-c_2m} \end{aligned}$$

noting that $m=\omega (\log (n))$ and $b\in {{\mathrm{poly}}}(n)$, we have $b\le e^{cm}$ for any constant $c>0$

$$\begin{aligned}&\le \left( \frac{2^{-c_1d(n)}}{1-2^{-c_1}}\right) +e^{-(c_2-c)m+2\delta \ln (n)}\\&\le e^{-c_3d(n)}+e^{-(c_2-c)m+2\delta \ln (n)} \end{aligned}$$

for some constant $c_3>0$. By a union bound over all $t+1< 2e^{cd(n)}\le 2e^{cm/5}$ elements in $M_{t+1}$,

$$\begin{aligned} \Pr \left( p_{i+d(n)}\in M_{t+1}\right)&\le (t+1)(e^{-c_3d(n)}+e^{-(c_2-c)m+2\delta \ln (n)})\\&\le 2(e^{-(c_3-c)d(n)}+e^{-(c_2-6c/5)m+2\delta \ln (n)})\\&\le e^{-\Omega (d(n))}, \end{aligned}$$

where in the last inequality, we have chosen $c < \min (c_3, (5/6)c_2)$ and noted that $5d(n)\le m$ and $m=\omega (\log n)$. $\square$

Unless the algorithm knows the end point $p_\ell$, the fitness function does not reveal any information about the hidden sub-string *z*. However, if the path length is sufficiently long relative to $\mu$, any elitist $(\mu +\lambda )$ EA must with at least constant probability produce more than $\mu$ path points before the end point $p_\ell$ is discovered. From this point, the only way to discover the hidden sub-string *z* is to evaluate search points in region $C\cup D$. However, due to the elite nature of the algorithm, it is prevented from evaluating any search point in *C*. The only way for the algorithm to reach region *D* is to guess the bit string *z* correctly.

Previously it has been shown that the elitist black-box complexity of Slo$^\alpha _{\varepsilon ,r}$ is exponential for $\alpha ,\varepsilon$ being constant [13]. Using the same approach but with the new and inclusive definition of the hierarchy and more refined arguments, we have the following result which shows that the elitist black-box complexity is already exponential for a very mild deception $\varepsilon \in \omega (\log {n}/n)$ and constant density $\alpha$.

### Theorem 41

For any constant $\alpha \in (0,1),$ $\varepsilon \in \omega (\log {n}/n)$ and $\mu ,\lambda \in {{\mathrm{poly}}}(n)$, the elitist ($\mu$+$\lambda$) black-box complexity of Slo$^\alpha _{\varepsilon ,r}$ is $e^{\Omega (n)}$.

### Proof

We apply Theorem 2, or Yao’s minimax principle, and consider the average case runtime of any deterministic elitist $(\mu +\lambda )$ black-box algorithm $\textbf{A}$ w.r.t. a chosen distribution $p'$ over Slo$^\alpha _{\varepsilon ,r}$ functions.

We first justify the finiteness of the sets of inputs and algorithms when applying the principle. It will be clear later by our choice of $p'$ that the support of that distribution is finite, thus we only apply Yao’s minimax principle to a finite subset of Slo$^\alpha _{\varepsilon ,r}$ functions. However, by Lemma 3, the lower bound extends to the whole function class which may contain an infinite number of functions.

To avoid the issue of having an infinite number of elitist algorithms, we consider a relaxation to the restriction in Algorithm 3: in addition to $P_t$ and the ranking $\rho (P_t,f)$ the algorithm also knows the set $M_t$ of search points it has sampled so far when selecting the distribution $p^{(t)}$ in line 7. However, note that the algorithm does not know the entire ranking of all solutions in $M_t$ (only partially among those in $P_t$). This algorithmic model is clearly more powerful than our $(\mu +\lambda )$ elitist black-box model, and any lower bound on the running time for the former also holds for the latter. Furthermore, any optimal deterministic algorithm in this superset of algorithms must not sample a search point twice given the availability of $M_t$. Therefore, it suffices to consider the set of such algorithms when applying Theorem 2. Each algorithm can be modelled as a decision tree, i.e., to choose which search point to query next based on the current information, with depth $2^n$ and maximum degree $2^n-1$ (since the root is a fixed point). The number of such trees is finite and so is the set of algorithms.

Fix any $\delta <1$, we construct $p'$ from the distribution *p* over BBFunnel with path length $b=\mu n^{2\delta }\in {{\,\textrm{poly}\,}}(n)$, and parameters $w:=(1-\alpha )n, v:=(2/3)(1-\alpha )n,$ and $u:=\max ((1/3)(1-\alpha )n,v-n/2)=(1/3)(1-\alpha )n$. Define $m:=v-u$ $=(1/3)(1-\alpha )n$, and note that $m\in \omega (\log {n})$. Given a function *f* sampled according to distribution *p*, let *F* be the event that *f* has simple path length $\ell <n^\delta \mu$ or $f\not \in \textsc{Slo}^\alpha _{\varepsilon ,r}$. By Theorem 39, Lemma 36 and a union bound, the probability of event *F* is less than $\sigma +2^{-\Omega (n^\delta )}$ where $\sigma :=1-74/100$. To sample from distribution $p',$ we sample *f* according to *p*, and return $\textsc{ OneMax} \in \textsc{Slo}^1_0$ if *F* occurs, and *f* otherwise.

When event *F* occurs, we use the lower bound $T(I_{p'},\textbf{A})\ge 0$. Otherwise, if event *F* does not occur, we first claim that with overwhelmingly high probably, the algorithm will not query any search point in region *C* before finding the optimum.

To prove the claim, we distinguish between two cases depending on the number of queries on the path before querying the optimum. In case 1, the algorithm queries less than $\mu$ elements on the path before discovering the optimum or $p_\ell$. In case 2, the algorithm queries at least $\mu$ elements on the path before querying the optimum or $p_\ell$.

We now prove the claim in case 1. By Lemma 40 for $d(n)=n^{\delta /2}$ and a union bound, with probability at least $1-\mu e^{-\Omega (n^{\delta /2})}$, the last point $p_i$ discovered on the path before point $p_\ell$ has index $i<\mu d(n)=\mu n^{\delta /2}\le \ell -\Omega (\mu n^{\delta })$, that is, we do not progress along the path by more than *d*(*n*) in every $\mu$ queries. Hence, the algorithm has not queried the last path point $p_\ell$ before the optimum and will therefore never query a search point in the sub-optimal region *C* before finding the optimum.

We then prove the claim in case 2. By Lemma 40 with $d(n):=n^\delta$, with probability $1-e^{-\Omega (n^\delta )}$, algorithm $\textbf{A}$ must query at least $\ell /n^\delta \ge \mu$ of the points in the path region *B* before it obtains the final point $p_\ell$. The fitness of these search points is higher than that of any search point in *C*, hence once the algorithm has obtained path point $p_\ell$, the population contains only points in *B*. With $\mu$ elements in *B*, the algorithm cannot base any further decisions on the fitness values in region *C*.

The claim therefore holds in both cases. Hence, in order to reach region *D*, the algorithm must find the hidden sub-string *z* without querying any elements in region *C*. The probability that *z* is found in any of the $e^{\Omega (n)}$ next queries made by the algorithm is exponentially small. Thus, $E\left[ T(I_{p'},\textbf{A}) | F \text{ does } \text{ not } \text{ occur }\right] = e^{\Omega (n)}$.

By the law of total probability w.r.t. *F*, $\min _{\textbf{A} \in \mathcal {A}} E\left[ T(I_{p'},\textbf{A})\right] = e^{\Omega (n)}$, which by Yao’s principle implies the theorem. $\square$

## Non-Elitist Algorithms

In this section, we show that some non-elitist EAs within the framework of Algorithm 1 and with appropriate settings can efficiently solve all problems of some specific SLO-hierarchy with constant $\alpha ,\varepsilon$ and $\varepsilon <\alpha$. This is in contrast with the inefficiency of all elitist ($\mu$+$\lambda$) black-box algorithms as previously shown in Theorem 41. We then demonstrate that the relation between $\alpha ,\varepsilon$ of SLOs that are solvable by non-elitist EAs and the parameters of these algorithms can be derived with further detailed analysis.

### Level-based Analysis with Deceptive Regions

The level-based theorem [5] is a tool for deriving upper bounds on the expected runtime of Algorithm 2. To derive sufficient conditions for the efficiency of non-elitist EAs on Slo$^\alpha _{\varepsilon ,r}$, we generalise the theorem by introducing a “deceptive region” *B*. The new conditions (G1) and (G2) are weakened (compared to those in [5]), and only required to hold if there are few individuals in the deceptive region. A new condition (G0) requires that the number of deceptive individuals reduces in expectation by a $(1-\varepsilon ')$-factor if it is above some threshold $\psi _0\lambda$. This variant of the level-based theorem has been implicitly used before, including [9] and [3].

#### Theorem 42

Consider Algorithm 2 with population size $$\lambda $$. Given a partition (*A*_1_, …, *A*_*m*_) of $$\mathcal {X}$$ and a subset $$B\subset \mathcal {X}$$, define $$T := \min$$ $$\{t\lambda \mid |P_t\cap A_{m}|>0\}$$, where for all $$t\in \mathbb {N}$$, $$P_t\in \mathcal {X}^\lambda $$ is the population in generation *t*. If there exist $$z_1,\dots ,z_{m-1}, \delta $$$$ \in (0,1]$$, $$\varepsilon '\in (0,\min (\delta ,2/3)],$$ and $$\psi _0,\gamma _0 \in (0,1),$$ $$\psi _0\ge \gamma _0$$ such that for any population $$P\in \mathcal {X}^\lambda $$, $$y \sim D(P)$$, any $$j \le m-1$$, the following conditions hold

(G0): for any $\psi \ge \psi _0$ if $|P\cap B|\le \psi \lambda$ then $\Pr \left( y\in B\right) \le (1-\varepsilon ')\psi ,$
(G1): if $|P\cap B|\le \psi _0\lambda$ and $|P\cap A_{\ge j}| \ge \gamma _0\lambda$, then $\Pr \left( y\in A_{\ge j+1} \right) \ge z_j,$
(G2): for any $\gamma \le \gamma _0$ if $|P\cap B|\le \psi _0\lambda$ and $|P\cap A_{\ge j}| \ge \gamma _0\lambda$ and $|P\cap A_{\ge j+1}| \ge \gamma \lambda$, then $\Pr \left( y\in A_{\ge j+1}\right) \ge (1+\delta )\gamma ,$
(G3): $$\displaystyle \lambda \ge \left( \frac{12}{\gamma _0 (\varepsilon ')^2}\right) \ln \left( \frac{300m}{z_* \varepsilon ' \delta \psi _0}\right) ,\, \mathrm{where}\, z_*:=\min _j z_j, $$ then $$ E\left[ T\right] \le \frac{72\lambda }{5\varepsilon '\psi _0}+ \frac{96}{\delta ^{2}} \sum _{j=1}^{m-1} \left( \lambda \ln \left( \frac{6\delta \lambda }{4+z_j\delta \lambda }\right) +\frac{1}{z_j}\right) . $$

#### Proof

The proof applies the level-based theorem [5]. Conditions (G1) and (G2) are identical to those in the level-based theorem, except for the additional assumptions on the number of individuals in the population *P* that belongs to set *B*. We call these individuals *deceptive individuals*. It is also easy to see that condition (G3) satisfies (G3) of the level-based theorem in [5], namely

$$\begin{aligned} \lambda \ge \frac{4}{\gamma _0\delta ^2} \ln \left( \frac{128 m}{z_* \delta ^2}\right) , \end{aligned}$$

since larger constants are used, $\varepsilon '\le \delta$, and $\gamma _0\le \psi _0<1$.

We divide the run into phases of length $\tau _1+2\tau _2$, where $\tau _1$ and $\tau _2$ will be specified later. We will prove that unless a low-probability failure event occurs, within the first sub-phase of $\tau _1$ generations, the number of deceptive individuals reduces to no more than $\psi _0\lambda$. We will then prove that unless a failure occurs, during the second sub-phase of $2\tau _2$ generations, the number of deceptive individuals remains below $\psi _0\lambda$, and through application of the level-based theorem, the optimum is found by the end of the phase. The final result will be obtained by considering multiple phases, taking into account the probability of failures.

To analyse the number of deceptive individuals, define $Y_t:=|P_{t}\cap B|$. Let $y\sim D(P_t)$, then note that when $Y_t\ge \psi _0$, which means there exists a $\psi \ge \psi _0$ such that $Y_t=|P_t \cap B|=\psi \lambda$, condition (G0) implies that the probability of *y* ending up in *B* is at most $(1-\varepsilon ')\psi = (1-\varepsilon ')Y_t/\lambda$. Otherwise when $Y_t\le \psi _0$, which means $Y_t=|P_t \cap B|\le \psi _0\lambda$, (G0) implies that the probability of *y* being in *B* is at most $(1-\varepsilon ')\psi _0$. Since $\lambda$ search points like *y* are sampled independently in each generation, $Y_{t+1}$ is stochastically dominated by the random variable $Z\sim {{\,\textrm{Bin}\,}}(\lambda ,p_s)$ where $p_s:=\max (\psi _0,Y_t/\lambda )(1-\varepsilon ')$.

We will say that a *failure* event occurs during sub-phase 1 whenever $Y_{t+1}\ge \max (\psi _0\lambda ,(1-\varepsilon '/2) Y_t)$. To bound the probability of failure in any given generation of the first sub-phase, we will apply the Chernoff bound in Lemma 55 with respect to *Z* and $\kappa =\varepsilon '/(2(1-\varepsilon '))$. Note that the conditions of Lemma 55 are satisfied because the assumption $\varepsilon '\le 2/3$ implies $\varepsilon '/(2(1-\varepsilon '))\le 1.$

22 $$\begin{aligned} \Pr \left( Y_{t+1}\ge \max (\psi _0\lambda ,(1-\varepsilon '/2) Y_t)\right)&< \Pr \left( Z\ge (1-\varepsilon '/2) \max (\psi _0\lambda ,Y_t)\right) \nonumber \\&= \Pr \left( Z\ge E\left[ Z\right] \left( 1+\varepsilon '/(2(1-\varepsilon '))\right) \right) \nonumber \\&\le \exp \left( -\frac{(\varepsilon ')^2\max (\psi _0\lambda ,Y_t)(1-\varepsilon ')}{12(1-\varepsilon ')^2}\right) \nonumber \\&< \exp \left( -\frac{(\varepsilon ')^2 \psi _0\lambda }{12}\right) . \end{aligned}$$

We now define the duration of the first sub-phase to be

$$\begin{aligned} \tau _1&:= \frac{\ln (\psi _0)}{\ln (1-\varepsilon '/2)} = \frac{\ln (1/\psi _0)}{\ln (\frac{1}{1-\varepsilon '/2})} = \frac{\ln (1/\psi _0)}{\ln (1+\frac{\varepsilon '}{2-\varepsilon '})}, \end{aligned}$$

by Lemmas 56, this is

23 $$\begin{aligned}&\le \left( \frac{1/\psi _0-1}{2}\right) \left( \frac{2+1/\psi _0-1}{1+1/\psi _0-1}\right) \left( \frac{2-\varepsilon '}{2\varepsilon '}\right) \left( \frac{\varepsilon '}{2-\varepsilon '} + 2\right) \nonumber \\&= \left( \frac{1-\psi _0}{2\psi _0}\right) \left( \frac{1+1/\psi _0}{1/\psi _0}\right) \left( \frac{2-\varepsilon '}{2\varepsilon '}\right) \left( \frac{4}{2-\varepsilon '}\right) \nonumber \\&= \left( \frac{1-\psi _0}{2\psi _0}\right) \left( \psi _0+1\right) \left( \frac{2}{\varepsilon '}\right) = \frac{(1-\psi _0)^2}{\psi _0 \varepsilon '} < \frac{1}{\varepsilon '\psi _0}. \end{aligned}$$

If no failure occurs during sub-phase 1, then the number of deceptive individuals at the end of this sub-phase is no more than $\lambda (1-\varepsilon '/2)^{\tau _1}=\psi _0\lambda$.

The second sub-phase lasts for $2\tau _2$ generations, where we define

24 $$\begin{aligned} \tau _2&:=\left( \frac{8}{\delta ^{2}}\right) \sum _{j=1}^{m-1} \left( \ln \left( \frac{6\delta \lambda }{4+z_j\delta \lambda }\right) +\frac{1}{z_j\lambda }\right) \nonumber \\&\le \frac{8m}{\delta ^2}\left( \ln (6/z_*)+\frac{1}{\lambda z_*}\right)< \frac{8m}{\delta ^2z_*}\left( 6+\frac{1}{\lambda }\right) < \frac{146m}{3\delta ^2z_*}, \end{aligned}$$

where the last inequality applied $\lambda >12$ which follows from condition (G3). Note that $\tau _2$ is the expected time to reach level *m* in the original level-based theorem [5].

Now by Eqs. (22),(23),(24), condition (G3), and a union bound, with probability no more than

25 $$\begin{aligned} (\tau _1+2\tau _2)\exp \left( -\frac{(\varepsilon ')^2\psi _0\lambda }{12}\right)&\le \left( \frac{1}{\psi _0\varepsilon '}+\frac{292m}{3\delta ^2z_*}\right) \left( \frac{z_* \varepsilon ' \delta \psi _0}{300m}\right) \nonumber \\&= \frac{z_*\delta }{300m}+\frac{292\varepsilon '\psi _0}{3\delta \cdot 300}\nonumber \\&< \frac{1}{300} + \frac{292}{900} = \frac{295}{900} < \frac{1}{3}, \end{aligned}$$

the number of deceptive individuals is more than $\psi _0\lambda$ during the second sub-phase of $2\tau _2$ generations. In the second last inequality above, we used that $\varepsilon '<\delta$.

By Markov’s inequality and the level-based theorem [5], with probability no more than 1/2, the algorithm fails to reach level *m* after $2\tau _2$ generations of the phase. Inversely, by a union bound and Eq. (25), a phase is successful with probability $1-1/2-1/3=1/6$. If the phase is unsuccessful, we can apply the same arguments to the next phase, since we have not assumed anything about the initial state of the population.

Hence, a successful phase occurs in expectation after at most 6 phases, i.e., $6(\tau _1+2\tau _2)$ generations. The theorem now follows because each generation produces $\lambda$ individuals. $\square$

We now derive a corollary result to the theorem that is tailored to implementations of operator *D* which combines a *f*-monotone selection and the bitwise mutation operator, denoted by $p_\textrm{mut}$. The following lemmas describe the transition probabilities of search points on dense and sparse regions of the search space when the mutation operator is applied to them.

#### Lemma 43

Let $x\in \{0,1\}^n$ and $y\sim p_\textrm{mut} (x)$. If a set *C* is $\alpha$-dense set with respect to a set $D\supseteq C$ and if $x\in C$, then

26 $$\begin{aligned} \Pr \left( y\in D\right) > \left( 1-\chi /n\right) ^n(1+\alpha \chi ). \end{aligned}$$

#### Proof of Lemma 43

To obtain an element in *D* from *x* via mutation, it suffices to either flip no bits so that *x* remains in $C\subseteq D$, or to mutate into one of the at least $\alpha n$ Hamming-neighbours of *x* in *D*, each obtained by flipping exactly one specific bit. The probability of this event is

$$\begin{aligned} \left( 1-\frac{\chi }{n}\right) ^n + \alpha n \left( \frac{\chi }{n}\right) \left( 1-\frac{\chi }{n}\right) ^{n-1}&> \left( 1-\frac{\chi }{n}\right) ^n\left( 1+\alpha \chi \right) . \end{aligned}$$

$$\square $$

#### Lemma 44

Let $\varepsilon \in [0,1]$, $r_{\max }\in [\lfloor n/2\rfloor ]$, and $\chi \in [0,n/2]$ be such that $(e\chi /r_{\max })^{r_{\max }}\le \varepsilon \left( 1-\frac{\chi }{n}\right) ^n$, $x\in \{0,1\}^n$ and $y\sim p_\textrm{mut} (x)$. For any $(\varepsilon ,r_{\max })$-sparse set $B\subset \{0,1\}^n$, it holds

(1): $$\Pr \left( y\in B\right) \le \left( 1-\frac{\chi }{n}\right) ^n+ \varepsilon $$ if $$x\in B$$,
(2): $$\Pr \left( y\in B\right) \le \varepsilon $$ if $$x\notin B$$.

#### Proof

Let $y\sim p_\textrm{mut} (x)$ be the mutated search point from *x*. By the union bound, the probability that *y* has a Hamming distance at least $r_{\max }$ to the parent *x* is at most

27 $$\begin{aligned} \Pr \left( H(x,y)\ge r_{\max }\right)&\le {n\atopwithdelims ()r_{\max }}\left( \frac{\chi }{n}\right) ^{r_{\max }}\nonumber \\&\le \left( \frac{en}{r_{\max }}\right) ^{r_{\max }}\left( \frac{\chi }{n}\right) ^{r_{\max }} \le \varepsilon \left( 1-\frac{\chi }{n}\right) ^n. \end{aligned}$$

If we start with parent $x\in B$, by the law of total probability,

$$\begin{aligned} \Pr \left( y\in B\right) =&\sum _{r=0}^n\Pr \left( y\in B\mid H(x,y)=r\right) \Pr \left( H(x,y)=r\right) \\ \le&\Pr \left( H(x,y)=0\right) + \sum _{r=1}^{r_{\max }-1}\frac{|S_r(x)\cap B|}{{n\atopwithdelims ()r}}\Pr \left( H(x,y)=r\right) \\&+\Pr \left( H(x,y)\ge r_{\max }\right) , \end{aligned}$$

tinequality (1) implies, this is

$$\begin{aligned} \le&\Pr \left( H(x,y)=0\right) \\&+\varepsilon \left( 1-\Pr \left( H(x,y)=0\right) -\Pr \left( H(x,y)\ge r_{\max }\right) \right) \\&+\Pr \left( H(x,y)\ge r_{\max }\right) \\ =&\left( 1-\frac{\chi }{n}\right) ^n(1-\varepsilon ) + \varepsilon +(1-\varepsilon )\Pr \left( H(x,y)\ge r_{\max }\right) ,\\ \end{aligned}$$

by (27), this is

$$\begin{aligned} \le&\left( 1-\frac{\chi }{n}\right) ^n(1-\varepsilon ) + \varepsilon +(1-\varepsilon )\varepsilon \left( 1-\frac{\chi }{n}\right) ^n \\ =&\left( 1-\frac{\chi }{n}\right) ^n(1-\varepsilon ^2) + \varepsilon < \left( 1-\frac{\chi }{n}\right) ^n + \varepsilon . \end{aligned}$$

Similarly, if we start with parent $x\notin B$, by the law of total probability

$$\begin{aligned} \Pr \left( y\in B\right) =&\; \sum _{r=0}^n\Pr \left( y\in B\mid H(x,y)=r\right) \Pr \left( H(x,y)=r\right) \\ \le&\; \sum _{r=1}^{r_{\max }}\frac{|S_r(x)\cap B|}{{n\atopwithdelims ()r}}\Pr \left( H(x,y)=r\right) +\Pr \left( H(x,y)\ge r_{\max }\right) , \end{aligned}$$

inequality (1) implies, this is

$$\begin{aligned} <&\; \varepsilon \left( 1-\Pr \left( H(x,y)=0\right) -\Pr \left( H(x,y)\ge r_{\max }\right) \right) + \Pr \left( H(x,y)\ge r_{\max }\right) \\ =&\; \varepsilon \left( 1-\left( 1-\frac{\chi }{n}\right) ^{n}\right) + (1-\varepsilon )\Pr \left( H(x,y)\ge r_{\max }\right) , \end{aligned}$$

Eq. (27) implies

$$\begin{aligned} \le&\; \varepsilon \left( 1-\left( 1-\frac{\chi }{n}\right) ^{n}\right) + (1-\varepsilon )\varepsilon \left( 1-\frac{\chi }{n}\right) ^n\\ =&\; \varepsilon \left( 1-\varepsilon \left( 1-\frac{\chi }{n}\right) ^n\right) <\varepsilon . \end{aligned}$$

$$\square $$

The following theorem, a special case of Theorem 42, gives sufficient conditions for non-elitist evolutionary algorithms that fit the framework of Algorithm 1 to efficiently optimise Slo$^\alpha _{\varepsilon ,r}$ using bitwise mutation and a selection mechanism characterised by its $\beta (\psi ,\gamma )$ function (c.f. Definition 1). Recall that $\beta (\psi ,\gamma )$ is the probability that the selection mechanism chooses an individual with rank in the interval from $\lceil \psi \lambda \rceil$ to $\lceil \gamma \lambda \rceil$. Conditions (SM2a) and (SM2b) are lower bounds on the selective pressure, and relate to condition (G2) in the level-based theorem (Theorem 42). Condition (SM2a) ensures that the fittest $\gamma _0\lambda$ individuals have sufficiently high selective pressure relative to the mutation rate $\chi$. Condition (SM2b) ensures that condition (G2) from the level-based theorem is satisfied with respect to individuals in fitness valleys, i.e., potentially with ranks higher than $\psi _0\lambda$. Condition (SM0) is an upper bound on the selective pressure. It ensures that individuals on fitness peaks do not occupy an overly large fraction of the population. Finally, condition (SM3) is an equivalent to condition (G3) in the level-based theorem, stating that the population size must be sufficiently large.

#### Theorem 45

If there exist constants $\psi _0,\gamma _0,\alpha \in (0,1),$ with $\psi _0\ge \gamma _0$ and $\psi _0+2\gamma _0\le 1$, and $\delta \in (0,1), \varepsilon '\in (0,\min (\delta ,2/3)]$ with $1/\varepsilon '\in {{\,\textrm{poly}\,}}(n)$, and $\varepsilon \in (0,\psi _0)$, and $r\in [\lfloor n/2 \rfloor ]$ such that Algorithm 1 with the bitwise mutation operator with rate $\chi \in (0,n/2]$ satisfying $1/\chi \in {{\,\textrm{poly}\,}}(n)$ and $(e\chi /r)^{r}\le \varepsilon \left( 1-\frac{\chi }{n}\right) ^n$, and an *f*-monotone selection mechanism with selection probability function $\beta$, and population size $\lambda$ satisfying

(SM0): $\beta (0,\gamma )\le \frac{\gamma (1-\varepsilon ')-\varepsilon }{\left( 1-\frac{\chi }{n}\right) ^n}$ for all $\gamma \in [\psi _0,1]$,
(SM2a): $\beta (0,\gamma )\ge \frac{\gamma (1+\delta )}{\left( 1-\frac{\chi }{n}\right) ^{n}}$ for all $\gamma \in (0,\gamma _0]$,
(SM2b): $\beta (\psi _0+\gamma _0,\psi _0+\gamma _0+\gamma )\ge \frac{\gamma (1+\delta )}{\left( 1-\frac{\chi }{n}\right) ^n(1+\alpha \chi )}$ for all $\gamma \in (0,\gamma _0]$,
(SM3): $\frac{c}{(\varepsilon ')^2}\ln (n)\le \lambda \in {{\,\textrm{poly}\,}}(n)$ for a sufficiently large constant *c* then Algorithm 1 has expected polynomial runtime on Slo$^\alpha _{\varepsilon ,r}$.

#### Proof

Consider any function *f* in the problem class, with an associated partition $(A_1,\ldots , A_m)$ and *f*-deceptive pairs $(A_{i_1},A_{j_1}),$ ..., $(A_{i_u},A_{j_u})$, where by definition $m\in {{\,\textrm{poly}\,}}(n)$. We will apply Theorem 42 with respect to $B:=\cup _{v=1}^u A_{i_v}$, which by the definition of the problem class is $(\varepsilon ,r)$-sparse.

To verify condition (G0), let us assume that the population contains $\gamma \lambda =|P\cap B|$ individuals in the *B*-region and the fittest among those, with respect to *f*, has rank *i* in *P*, and that $|P\cap B|\le \psi \lambda$ for some fixed $\psi \ge \psi _0$.

Let *x* be the selected parent and *y* be the the offspring obtained after mutating *x*, that is, $x=P(k)$ where $k\sim p_\textrm{sel} (P)$ and $y\sim p_\textrm{mut} (x)$. By the law of total probability, we get

$$\begin{aligned} \Pr \left( y\in B\right)&= \Pr \left( x\in B\right) \Pr \left( y\in B\mid x\in B\right) + \Pr \left( x\not \in B\right) \Pr \left( y\in B\mid x\not \in B\right) , \end{aligned}$$

then by Lemma 44 and the *f*-monotone property of selection given $0\le i/\lambda$, this is

$$\begin{aligned}&\le \beta (i/\lambda ,i/\lambda +\gamma )\left( \varepsilon + (1-\chi /n)^n\right) + (1-\beta (i/\lambda ,i/\lambda +\gamma ))\varepsilon \\&= \beta (i/\lambda ,i/\lambda +\gamma )(1-\chi /n)^n + \varepsilon \le \beta (0,\gamma )(1-\chi /n)^n + \varepsilon , \\ \end{aligned}$$

and since $\beta (0,\gamma )$ is cumulative, and $\gamma \le \psi$ (from $|P\cap B|\le \psi \lambda$), this is

$$\begin{aligned}&\le \beta (0,\psi )(1-\chi /n)^n + \varepsilon , \end{aligned}$$

now using (SM0) with $\psi$ in place of $\gamma$ (since $\psi \ge \psi _0$) gives

$$\begin{aligned}&\le (\psi (1-\varepsilon ')-\varepsilon ) + \varepsilon = \psi (1-\varepsilon '). \end{aligned}$$

Hence, condition (G0) is satisfied.

To verify condition (G1), assume that $|P\cap B|\le \psi _0\lambda$ and $|P\cap A_{\ge j}|\ge \gamma _0\lambda$, and that the fittest individual of $P\cap A_{\ge j}$ has rank *i* in *P*. Notice that $i\le \psi _0 \lambda$ because otherwise if $i> \lceil \psi _0\lambda \rceil$ then this implies that there exists an individual in *P* that is fitter than those in $A_{\ge j}$ while that individual does not belong to *B*, and this contradicts the definition of the set *B*. Then, to produce an individual *y* in $A_{\ge j+1}$, it suffices to select an individual $x\in A_{\ge j}$, and flip at most $d=\mathord {\text {O}}\mathord {\left( 1\right) }$ bit-positions. Thus, by condition (SM2b) and the *f*-monotone property of the selection mechanism with $i/\lambda \le \psi _0$, we get

$$\begin{aligned} \Pr \left( y\in A_{\ge j+1}\right)&\ge \Pr \left( x\in A_{\ge j}\right) \Pr \left( y\in A_{\ge j+1}\mid x\in A_{\ge j}\right) \\&\ge \beta (i/\lambda ,i/\lambda +\gamma _0)\left( \chi /n\right) ^{d}\left( 1-\chi /n\right) ^{n-d}\\&\ge \beta (\psi _0,\psi _0+\gamma _0)\left( \chi /n\right) ^{d}\left( 1-\chi /n\right) ^{n-d}\\&\ge \beta (\psi _0+\gamma _0,\psi _0+2\gamma _0)\left( \chi /n\right) ^{d}\left( 1-\chi /n\right) ^{n-d}\\&\ge \frac{\gamma _0(1+\delta )}{\left( 1-\chi /n\right) ^n(1+\alpha \chi )}\left( \chi /n\right) ^{d}\left( 1-\chi /n\right) ^{n-d}=: z_j. \end{aligned}$$

Hence, by $d=O(1)$ and $1/\chi \in {{\,\textrm{poly}\,}}(n),$ condition (G1) is satisfied for some parameter $z_j\in 1/{{\,\textrm{poly}\,}}(n)$.

We now verify condition (G2). Assume that $|P\cap B|=\psi \lambda \le \psi _0\lambda ,$ $|P\cap A_{\ge j}| \ge \gamma _0\lambda$ and $|P\cap A_{\ge j+1}| \ge \gamma \lambda$. Zero or more of the levels $A_{j+1}, A_{j+2},\ldots , A_{m-1}$ may be in a fitness valley. To account for the lower selection probability in these levels, we define the set

$$\begin{aligned} B_{\ge j+1} := \bigcup \left\{ A_i\mid (A_i,A_k)\,\mathrm{is}\,f\text{-deceptive for}\,i\le j<k\right\} . \end{aligned}$$

corresponding to deceptive levels before level $j+1$ with respect to levels in $A_{\ge j+1}$. Note that $B_{\ge j+1}\subseteq B$. Furthermore, define

$$\begin{aligned} A^-_{\ge j+1} := \bigcup \left\{ A_k\mid \exists i\,\text{ s.t.}\, i\le j<k\le m-1\,\,\mathrm{and}\,\, (A_i,A_k)\,\mathrm{is}\,f\mathrm{-deceptive}\right\} . \end{aligned}$$

corresponding to the levels in $A_{\ge j+1}$ which are in a fitness valley with respect to levels prior to level *j*. Furthermore, define

$$\begin{aligned} A^+_{\ge j+1} := A_{\ge j+1} {\setminus} A^-_{\ge j+1} \end{aligned}$$

corresponding to the levels which are not in a fitness valley with respect to levels prior to level *j*. Let

$$\begin{aligned} \gamma ^- := \frac{1}{\lambda }|P\cap A^-_{\ge j+1}|\quad\mathrm{and}\quad\gamma ^+ := \frac{1}{\lambda }|P\cap A^+_{\ge j+1}|. \end{aligned}$$

It immediately follows from these definitions that $\gamma =\gamma ^-+\gamma ^+$.

By the definitions above, in the worst case with respect to selection probabilities, the $\gamma ^+\lambda$ fittest elements in the population are in the set $A^+_{\ge j+1}$ since these levels by definition do not form part of a deceptive pair. They are followed by at most $|P\cap B_{\ge j+1}|\le |P\cap B|\le \psi _0\lambda$ individuals in $B_{\ge j+1}$, followed by $\gamma ^-\lambda$ individuals in $A^-_{\ge j+1}$, since these levels are part of a deceptive pair.

Again, let *x* be the selected individual, i.e., $x=P(k)$ where $k\sim p_\textrm{sel} (P)$, and $y\sim p_\textrm{mut} (x)$ be the offspring after mutation.

Lemma 43, the $\alpha$-density of $A_{\ge j + 1}$, and (SM2b) now imply

$$\begin{aligned}&\Pr \left( x\in A^-_{\ge j+1}\right) \Pr \left( y\in A_{\ge j+1}\mid x\in A^-_{\ge j+1}\right) \\&\quad \quad \ge \beta (\psi _0+\gamma ^+,\psi _0+\gamma ^++\gamma ^-)\left( 1-\chi /n\right) ^n(1+\alpha \chi )\\&\quad \quad \ge \beta (\psi _0+\gamma _0,\psi _0+\gamma _0+\gamma ^-)\left( 1-\chi /n\right) ^n(1+\alpha \chi ) \ge \gamma ^-(1+\delta ). \end{aligned}$$

If the algorithm selects an individual in the highest levels $A^+_{\ge j+1}$, it suffices that the algorithm does not mutate any of the bits. By condition (SM2a), we get probability

$$\begin{aligned} \Pr \left( x\in A^+_{\ge j+1}\right) \Pr \left( y\in A_{\ge j+1}\mid x\in A^+_{\ge j+1}\right)&\ge \beta (0,\gamma ^+)\left( 1-\chi /n\right) ^n \ge \gamma ^+(1+\delta ). \end{aligned}$$

By the law of total probability,

$$\begin{aligned} \Pr \left( y\in A_{\ge j+1}\right)&\ge \Pr \left( x\in A^-_{\ge j+1}\right) \Pr \left( y\in A_{\ge j+1}\mid x\in A^-_{\ge j+1}\right) \\ & \quad +\Pr \left( x\in A^+_{\ge j+1}\right) \Pr \left( y\in A_{\ge j+1}\mid x\in A^+_{\ge j+1}\right) \\ &\ge \gamma ^-(1+\delta ) + \gamma ^+(1+\delta )\\ &= \gamma (1+\delta ). \end{aligned}$$

Hence, condition (G2) of Theorem 42 is satisfied.

We now verify condition (G3). Since $\varepsilon '\le \delta$, so $1/\varepsilon '\in {{\,\textrm{poly}\,}}(n)$ implies $1/\delta \in {{\,\textrm{poly}\,}}(n)$. Furthermore, $1/z_*=1/z_{\min }\in {{\,\textrm{poly}\,}}(n)$ and $m\in {{\,\textrm{poly}\,}}(n)$, and $\psi _0,\gamma _0\in \Theta (1)$, then it holds for a sufficiently large constant *c* that

$$\begin{aligned} \lambda&\ge \frac{c}{(\varepsilon ')^2}\log (n) \ge \left( \frac{12}{\gamma _0 (\varepsilon ')^2}\right) \ln \left( \frac{300m}{z_*\varepsilon '\delta \psi _0}\right) , \end{aligned}$$

hence condition (G3) is satisfied.

All conditions of Theorem 42 are satisfied. Recalling that $\psi _0\in \Theta (1)$, $m\in {{\,\textrm{poly}\,}}(n)$, $\lambda \in {{\,\textrm{poly}\,}}(n)$, $1/z_j\in {{\,\textrm{poly}\,}}(n)$, and $1/\delta \in {{\,\textrm{poly}\,}}(n)$ it follows that the expected runtime of Algorithm 1 on *f* is no more than

$$\begin{aligned} \frac{72\lambda }{5\varepsilon '\psi _0}+ \left( \frac{96}{\delta ^{2}}\right) \sum _{j=1}^{m-1} \left( \lambda \ln \left( \frac{6\delta \lambda }{4+z_j\delta \lambda }\right) +\frac{1}{z_j}\right)&\in {{\,\textrm{poly}\,}}(n). \end{aligned}$$

$$\square $$

Note that the original definition of Slo$^\alpha _{\varepsilon ,r}$ in [13] made a stronger assumption in condition 4, namely that for any *f*-deceptive pair $(A_i,A_j)$ that the whole set $A_{\ge j}$ is $\alpha$-dense w.r.t. $A_{\ge j}$ (in terms of the density Definition 4 from the current paper). Clearly, the set $A_{\ge j}$ contains the points of the “fitness valley” where the level set $A_j$ belongs to, but it may also contain search points in higher levels $A_k$ for $k>j$ which are not in a “fitness valley”. The stronger assumption from [13] is relaxed in this paper because the proof of Theorem 45 does not require that the elements in $A_k, \ k>j$ are $\alpha$-dense since they have strictly higher fitness than any search point in earlier levels $A_{<k}$, and an appropriately configured algorithm can multiply the number of individuals in $A_{\ge k}$ by selecting and duplicating them.

### Efficiency of Some Non-Elitist EAs

We now show how Theorem 45 can be applied to specific selection mechanisms. Particularly, we prove that many finely tuned non-elitist EAs are generally efficient in solving some SLO classes that are proven, from the previous section, to be hard for elitist EAs. The following corollary of Theorem 45 gives an example configuration of the *f*-monotone selection of 3-tournament where non-elitist EAs are efficient on Slo$^\alpha _{\varepsilon ,r}$.

It is noteworthy that the mutation rates in the following results have very specific numerical values which differ from the traditional mutation probability 1/*n* often recommended for elitist evolutionary algorithm. These mutation rates relate to the phenomenon of “error thresholds” in non-elitist evolutionary algorithms (see e.g.,  Theorem 4 in [29]) which provides a relationship between the selective pressure and the mutation rate. Informally, an evolutionary algorithm is said to have reproductive rate $\alpha _0$ if every individual has in expectation at most $\alpha _0$ offspring. A non-elitist evolutionary algorithm with mutation rate $\chi >\ln (\alpha _0)$, is said to be above the error threshold, and needs exponential time to find an optimum of any problem with a polynomial number of global optima.

For example, the non-elitist evolutionary algorithm in Corollary 48 has reproductive rate $\alpha _0\approx 2$. A mutation rate of $\chi =1$ would satisfy the condition $\chi >\ln (\alpha _0)\approx 0.693147$ of exponential runtime from [29]. Interestingly, choosing $\chi$ slightly less than the error threshold, in this case $\chi =0.693146$, is what is required to optimise $\textsc{Slo}^{4/9}_{1/100}$ in expected polynomial time. Condition (SM0) prevents a much smaller mutation rate $\chi$.

#### Corollary 46

Algorithm 1 with 3-tournament selection, population size $c\ln (n)$ $\le \lambda \in {{\,\textrm{poly}\,}}(n)$ for a sufficiently large constant *c*, and mutation rate $\chi =1.09812$ has polynomial worst case expected runtime on Slo$^\alpha _{\varepsilon ,r}$ with $\alpha =1/4,$ $\varepsilon =7/10^5$ and $r\ge 10$.

#### Proof of Corollary 46

Note that 3-tournament selection has $\beta$-function

$$\begin{aligned} \beta (\psi ,\psi +\gamma )= (1-\psi )^3 - (1-(\psi +\gamma ))^3 = \gamma \left( \gamma ^2 + 3 (1-\psi )(1-\gamma -\psi )\right) \end{aligned}$$

We evaluate the conditions of Theorem 45 numerically with the parameters

$$\begin{aligned} \psi _0 := \frac{1}{40},\quad \gamma _0 := \frac{1}{6250},\quad \delta := \frac{1}{30000} =: \varepsilon ' \end{aligned}$$

These parameters satisfy the assumptions of Theorem 45 because $\psi _0>\gamma _0$, $\psi _0+2\gamma _0<1$, $\varepsilon '=\delta$, $\varepsilon '<2/3$, and $\varepsilon <\psi _0$.

The following bounds hold for $\left( 1-\frac{\chi }{n}\right) ^n$, given that $n\ge 10^4$:

$$\begin{aligned} \left( 1-\frac{\chi }{n}\right) ^{n}&< e^{-\chi } < \frac{3335}{10000}, \\ \left( 1-\frac{\chi }{n}\right) ^{n}&= \left( 1-\frac{\chi }{n}\right) ^{\left( \frac{n}{\chi }-1\right) \chi }\left( 1-\frac{\chi }{n}\right) ^{\chi }\\&\ge e^{-\chi }\left( 1-\frac{\chi }{10^4}\right) ^{\chi } > \frac{3334}{10000}. \end{aligned}$$

The latter lower bound can be used for straightforward verification of the condition $(e\chi /r)^{r}\le \varepsilon \left( 1-\frac{\chi }{n}\right) ^n$ of Theorem 45 for $r\ge 10$, $\chi =1.09812$ and $\varepsilon =7/10^5$.

To verify conditions (SM0), (SM2a) and (SM2b), we define the following function.

$$\begin{aligned} h(\psi ,\gamma ) := \frac{\beta (\psi ,\psi +\gamma )}{\gamma } = \gamma ^2 + 3 (1-\psi )(1-\gamma -\psi ) \end{aligned}$$

Its derivatives are

$$\begin{aligned} \frac{\partial h(\psi ,\gamma )}{\partial \gamma }&= -3 + 2 \gamma + 3 \psi \le 0, \\ \mathrm{and}\quad \frac{\partial h(\psi ,\gamma )}{\partial \psi }&= -6 + 3 \gamma + 6 \psi \le -1. \end{aligned}$$

Thus, $h(\psi ,\gamma )$ is non-increasing in both $\psi$ and $\gamma$ for $(\psi ,\gamma ) \in [0,1/3] \times (0,1]$.

Hence, for all $\gamma \in [\psi _0,1] \subset (0,1]$ we have

$$\begin{aligned} \frac{\beta (0,\gamma )}{\gamma }&= h(0,\gamma ) \le h(0,\psi _0) = \frac{1}{40^2} + 3\left( 1-\frac{1}{40}\right) = \frac{4681}{1600} \\&< \frac{1-\varepsilon '-\varepsilon /\psi _0}{\frac{3335}{10000}} < \frac{1-\varepsilon '-\varepsilon /\gamma }{\left( 1-\frac{\chi }{n}\right) ^n}, \end{aligned}$$

which implies that condition (SM0) is satisfied.

Similarly, it holds for all $\gamma \in (0,\gamma _0] \subset (0,1]$

$$\begin{aligned} \frac{\beta (0,\gamma )}{\gamma }&= h(0,\gamma ) \ge h(0,\gamma _0) = \frac{1}{6250^2} + 3\left( 1-\frac{1}{6250}\right)> \frac{18747}{6250} \\&> \frac{1+30000^{-1}}{\frac{3334}{10000}} > \frac{1+\delta }{\left( 1-\frac{\chi }{n}\right) ^n}, \end{aligned}$$

thus condition (SM2a) is satisfied.

Furthermore, for all $\gamma \in (0,\gamma _0] \subset (0,1]$ and $\psi$ fixed to $\psi _0 + \gamma _0 \in [0,1/3]$

it holds

$$\begin{aligned} \frac{\beta (\psi _0 + \gamma _0, \psi _0 + \gamma _0 + \gamma )}{\gamma }&= h(\psi _0+\gamma _0,\gamma ) \ge h(\psi _0+\gamma _0,\gamma _0)> \frac{57}{20} \\&> \frac{1+30000^{-1}}{\frac{3334}{10000} \cdot (1 + \frac{109812}{10^5}\cdot \frac{1}{4})} > \frac{1+\delta }{\left( 1-\chi /n\right) ^n(1+\alpha \chi )}, \end{aligned}$$

thus condition (SM2b) is also satisfied. Finally, condition (SM3) is satisfied for a sufficiently large constant *c*. $\square$

Corollary 46 together with Propositions 21, 22 and 23 imply that the non-elitist EA with 3-tournament is efficient for some Knapsack, VCP, and MISP example instances that we discussed earlier.

#### Corollary 47

Algorithm 1 with 3-tournament selection, population size $c\ln (n)$ $\le \lambda \in {{\,\textrm{poly}\,}}(n)$ for a sufficiently large *c*, and mutation rate $\chi =1.09812$ has polynomially bounded expected runtime

- on the fitness function $f_{\textsc{Knapsack}}$ for $\textsc{Knapsack} _{u, v}$ problem if $v\le 7/10^5$ and $u(1-v)+v\ge 1/4$;
- on the fitness function $f_\textsc{MISP}$ for $\textsc{MISP} ^{u,v}(K_{1,n})$ problem and on the fitness function $f_\textsc{VCP}$ for $\textsc{VCP} ^{u,v}(K_{1,n})$ problem, if $v\in [1/4, u]$, and $10^5/7<n$.

In contrast to non-elitist EAs described by this corollary, the Randomised Local Search (RLS) algorithm has an infinite runtime on $\textsc{MISP} ^{u,v}(K_{1,n})$ for any $u,v \in (0,1)$, as it was noted in Sect. 4.4.2. The same applies to the RLS runtime on $\textsc{VCP} ^{u,v}(K_{1,n})$.

The ranking selection operator [23] is defined by means of a ranking function $\alpha :\mathbb {R}\rightarrow \mathbb {R}$, such that $\alpha (x)\ge 0$ for all $x\in [0,1]$, and $\int _0^1\alpha (x) dx = 1$. In ranking selection with ranking function $\alpha$, the probability of selecting an individual among $\gamma \lambda$ best individuals is $\int _0^\gamma \alpha (x)dx$. In the special case of *linear ranking*, the selection operator is parameterised by $\eta \in (1,2]$, and the ranking function is defined as $\alpha (\gamma ):=\eta (1 - 2\gamma ) + 2\gamma$.

Note that we can represent the linear ranking selection as a randomised combination of 2-tournament selection and 1-tournament selection (the uniform selection). Indeed, the probability distribution of a rank-based selection operator is completely determined by its function $\beta (\gamma )$, and in the case of the linear ranking selection, $\beta _{\eta }(\gamma )=\int _0^\gamma \alpha (x)dx={\gamma (\eta (1-\gamma )+\gamma )}$. At the same time, if we apply the 2-tournament with probability *p* and the 1-tournament otherwise, we will get a selection operator with $\beta _p(\gamma )= {p(1-(1-\gamma )^2)}+(1-p)(1-(1-\gamma ))$, and it is easy to see that $\beta _p(\gamma )=\beta _{\eta }(\gamma )$ for all $\gamma \in [0,1]$, if we assume $p:=\eta -1$. In a similar way one can randomly combine *k*-tournament and $(k+1)$-tournament selection for any integer $k>0$ thus obtaining any intermediate selection intensity.

#### Corollary 48

Algorithm 1 with either 2-tournament selection or linear ranking selection [31] for $\eta =2$, population size $c\ln (n)\le \lambda \in {{\,\textrm{poly}\,}}(n)$ for a sufficiently large *c*, and mutation rate $\chi =0.693146$ has polynomial worst case expected runtime on Slo$^\alpha _{\varepsilon ,r}$ with $\alpha =4/9$, $\varepsilon =1/100$, and $r\ge 10$.

#### Proof of Corollary 48

The proof is by Theorem 45. The structure of the proof is identical to the proof of Corollary 46, however we will need different numerical parameters.

Recall that linear ranking selection has a $\beta$-function deduced from [23, 31]

$$\begin{aligned} \beta (\gamma _1,\gamma _2) = \int _{\gamma _1}^{\gamma _2}\alpha _{\mathrm{linear}}(x) dx,\quad \mathrm{with}\quad \alpha _{\mathrm{linear}}(\gamma ) = \eta (1-2\gamma )+2\gamma . \end{aligned}$$

Thus both this selection with $\eta =2$ and 2-tournament selection have:

$$\begin{aligned} \beta (\psi ,\psi +\gamma ) = \gamma (2 - \gamma - 2\psi ). \end{aligned}$$

We evaluate the conditions of Theorem 45 numerically with the parameters

$$\begin{aligned} \psi _0&:= \frac{1}{5}, \gamma _0 := \frac{1}{10^7}, \delta := \frac{1}{10^{6}} =: \varepsilon '. \end{aligned}$$

These parameters satify the assumptions of Theorem 45 because $\psi _0>\gamma _0$, $\psi _0+2\gamma _0<1$, $\varepsilon '=\delta$, $\varepsilon '<2/3$ and $\varepsilon <\psi _0$.

The following bounds hold for $\left( 1-\frac{\chi }{n}\right) ^{n}$ and sufficiently large *n*, i.e., $n \ge 10^8$

$$\begin{aligned} \left( 1-\frac{\chi }{n}\right) ^{n}&< e^{-\chi } < \frac{50000060}{10^8}, \\ \left( 1-\frac{\chi }{n}\right) ^{n}&\ge e^{-\chi }\left( 1-\frac{\chi }{10^8}\right) ^{\chi } > \frac{50000058}{10^8}, \end{aligned}$$

then the requirement $(e\chi /r)^r\le \varepsilon (1-\chi /n)^n$ of Theorem 45 can be easily verified for $r \ge 10$, $\chi =0.689146$, and $\varepsilon =1/100$.

Note that the function

$$\begin{aligned} h(\psi ,\gamma ) := \frac{\beta (\psi ,\psi +\gamma )}{\gamma } = 2-\gamma -2\psi \end{aligned}$$

is decreasing in both $\psi$ and $\gamma$ on the domain of $[0,1]^2$.

Hence, for all $\gamma \in [\psi _0,1]$ we have

$$\begin{aligned} \frac{\beta (0,\gamma )}{\gamma }&= h(0,\gamma ) \le h(0,\psi _0) = \frac{9}{5} \\&< \frac{4749995}{2500003} = \frac{1-\varepsilon '-\varepsilon /\psi _0}{\frac{50000060}{10^8}} \le \frac{1-\varepsilon '-\varepsilon /\gamma }{\left( 1-\frac{\chi }{n}\right) ^n}, \end{aligned}$$

which implies that condition (SM0) is satisfied.

Similarly, it holds for all $\gamma \in (0,\gamma _0]$

$$\begin{aligned} \frac{\beta (0,\gamma )}{\gamma }&= h(0,\gamma ) \ge h(0,\gamma _0) = \frac{19999999}{10000000}\\&> \frac{50000050}{25000029} = \frac{1+\delta }{\frac{50000058}{10^8}} \ge \frac{1+\delta }{\left( 1-\frac{\chi }{n}\right) ^n}, \end{aligned}$$

thus condition (SM2a) is satisfied.

Furthermore, for all $\gamma \in (0,\gamma _0]$ and $\psi$ fixed to $\psi _0+\gamma _0$, it holds

$$\begin{aligned} \frac{\beta (\psi _0+\gamma _0,\psi _0+\gamma _0+\gamma )}{\gamma }&=h(\psi _0+\gamma _0,\gamma )\ge h(\psi _0+\gamma _0,\gamma _0) =\frac{15999997}{10^7} \\&>\frac{6250006250000}{4087707519513} = \frac{1+\delta }{\frac{50000058}{10^8}\cdot (1+\alpha \chi )}\\&\ge \frac{1+\delta }{\left( 1-\chi /n\right) ^n(1+\alpha \chi )}, \end{aligned}$$

thus condition (SM2b) is also satisfied. Finally, condition (SM3) is satisfied for a sufficiently large constant *c*. $\square$

Based on Corollary 48 together with Propositions 21 and 23 one can obtain polynomial runtime bounds for 2-tournament selection and linear ranking selection on the $\textsc{Knapsack} _{u, v}$ $\textsc{MISP} ^{u,v}(K_{1,n})$ and $\textsc{VCP} ^{u,v}(K_{1,n})$, analogous to those of Corollary 47.

### On the Impact of Selection and Mutation

We now turn out attention to how $\alpha$ and $\varepsilon$ impact the parameters of some parent selection mechanisms, namely the linear ranking and power-law ranking [14] selections, and of mutation for all Slo$^\alpha _{\varepsilon ,r}$ that can be efficiently solved by the corresponding non-elitist EAs.

The following lemma gives bounds on the probability of not flipping any bit using bitwise mutation with mutation parameter $\chi$.

#### Lemma 49

For any $\chi ,\delta >0$, let $n_0:= \frac{\chi }{1 - \left( \frac{1+\delta }{1+2\delta }\right) ^{1/\chi }}$, then for all natural numbers $n\ge n_0$ it holds

$$\begin{aligned} e^{-\chi } > \left( 1-\frac{\chi }{n}\right) ^n \ge \frac{1+\delta }{1+2\delta } \cdot e^{-\chi } \end{aligned}$$

#### Proof

The first inequality follows from $1+x\le e^x$ where $x=-\chi /n$ and from the choice of $n\ge n_0$ that $1-\chi /n > 0$. For the second inequality, since $n\ge n_0$ we get

$$\begin{aligned} \left( 1-\frac{\chi }{n}\right) ^n = \left( 1-\frac{\chi }{n}\right) ^{\chi + (n/\chi -1)\chi }&\ge \left( 1-\frac{\chi }{n_0}\right) ^{\chi } e^{-\chi } = \frac{1+\delta }{1+2\delta } \cdot e^{-\chi } \end{aligned}$$

and here we use $(1-1/x)^{x-1}\ge 1/e$ with *x* being $n/\chi$. $\square$

We have the following general result for linear ranking with parameter $\eta \in (1,2]$. The result essentially means that if

$$\begin{aligned} \varepsilon < \frac{\eta }{4(\eta -1)}\left( 1-\frac{1}{1+\alpha \ln {\eta }}\right) ^2 \end{aligned}$$

for any constants $\alpha ,\varepsilon \in (0,1)$ and $\eta \in (1,2]$, then the non-elitist algorithm as described in Algorithm 1 with linear ranking selection using parameter $\eta$ can optimise Slo$^{\alpha }_{\varepsilon ,\max \{8, \lceil 1-\ln {\varepsilon } \rceil \}}$ in expected polynomial time. Note that the fixed choice of $\eta =2$, $\alpha =4/9$ and $\varepsilon =1/100$ from Corollary 48 largely satisfies the above inequality, thus it is one special case of the general result.

#### Theorem 50

Let $\alpha , \varepsilon \in [0,1]$, $\delta \in (0,1)$ and $\eta \in (1,2]$ be constants such that $\eta > 1+3\delta$ and

28 $$\begin{aligned} \frac{(1+2\delta )^2 \eta }{4(1+3\delta )(\eta -1)}\left( 1-\frac{1}{1+\alpha \ln (\eta /(1+3\delta ))}\right) ^2 - 2\delta = \varepsilon . \end{aligned}$$

Algorithm 1 with the linear ranking selection with parameter $\eta$, the bitwise mutation with parameter $\chi =\ln (\eta /(1+3\delta ))$, and with population size $\lambda \in {{\,\textrm{poly}\,}}(n)$ such that $\lambda \ge c\ln {n}$ for a sufficiently large constant $c>0$ has polynomial worst case expected runtime on Slo$^\alpha _{\varepsilon ,r}$ for any $r \ge \max \{8, \lceil 1-\ln {\varepsilon } \rceil \}$.

#### Proof

For linear ranking, we have

$$\begin{aligned} \beta (\psi ,\psi +\gamma )&= \int _{\psi }^{\psi +\gamma } (\eta - 2(\eta - 1)x)dx = \left[ \eta x - (\eta - 1)x^2 \right] _{\psi }^{\psi +\gamma }\\&= \eta (\psi +\gamma - \psi ) - (\eta - 1)\left( (\psi +\gamma )^2 - \psi ^2 \right) \\&= \gamma (\eta - (\eta - 1)(\gamma + 2\psi )). \end{aligned}$$

Let $h(\psi ,\gamma ) :=\beta (\psi ,\psi +\gamma )/\gamma = \eta - (\eta - 1)(\gamma + 2\psi )$, and we note that *h* is linearly decreasing in both directions of $\gamma$ and $\psi$.

To prove the result, we apply Theorem 45 with parameter $\delta$ the same as the one in (28) and with the other parameters:

$$\begin{aligned} \varepsilon '&:= \min \left\{ \delta ,\frac{2}{3}\right\} ,\\ \psi _0&:= \sqrt{\frac{(2\delta + \varepsilon ) \eta }{(1+3\delta )(\eta -1)}} = \sqrt{\frac{(2\delta + \varepsilon ) e^\chi }{\eta -1}},\\ \gamma _0&:= \frac{1}{3}\cdot \frac{\delta \eta }{(1+3\delta )(\eta -1)}\left( 1-\frac{1}{1+\alpha \ln (\eta /(1+3\delta ))}\right) \\&= \frac{1}{3}\cdot \frac{\delta e^\chi }{\eta -1}\left( 1-\frac{1}{1+\alpha \chi }\right) < \frac{1}{3}\cdot \frac{\delta e^\chi }{\eta -1}. \end{aligned}$$

Clearly this setting is appropriate for $\varepsilon '$, i.e., $\varepsilon \le \min (\delta ,2/3)$. We now justify that it is also appropriate for $\psi _0$ and $\gamma _0$, that is $\psi _0 + 2\gamma _0<1$ and $\psi _0\ge \gamma _0$ as required by the theorem. Equation (28) can be written as

$$\begin{aligned} \left( \frac{(1+2\delta ) \eta }{2(1+3\delta )(\eta -1)}\right) ^2 \left( 1-\frac{1}{1+\alpha \ln (\eta /(1+3\delta ))}\right) ^2 = \frac{(2\delta + \varepsilon ) \eta }{(1+3\delta )(\eta -1)} = \psi _0^2, \end{aligned}$$

then taking square root both sides and using $\chi =\ln (\eta /(1+3\delta ))$ give

$$\begin{aligned} \psi _0&= \left( \frac{(1+2\delta ) \eta }{2(1+3\delta )(\eta -1)}\right) \left( 1-\frac{1}{1+\alpha \ln (\eta /(1+3\delta ))}\right) \\&= \left( \frac{(1+2\delta ) e^{\chi }}{2(\eta -1)}\right) \left( 1-\frac{1}{1+\alpha \chi }\right) , \end{aligned}$$

thus $\psi _0/\gamma _0=3(1+2\delta )/(2\delta )>1$ or $\psi _0>\gamma _0$. Furthermore,

29 $$\begin{aligned} 2\psi _0 + 3\gamma _0&= 2\left( \frac{(1+2\delta ) e^{\chi }}{2(\eta -1)}\right) \left( 1-\frac{1}{1+\alpha \chi }\right) + 3\cdot \frac{1}{3}\cdot \frac{\delta e^\chi }{\eta -1}\left( 1-\frac{1}{1+\alpha \chi }\right) \nonumber \\&= \left( \frac{(1+3\delta ) e^{\chi }}{\eta -1}\right) \left( 1-\frac{1}{1+\alpha \chi }\right) \nonumber \\&= \left( \frac{\eta }{\eta -1}\right) \left( 1-\frac{1}{1+\alpha \chi }\right) . \end{aligned}$$

Therefore it holds for $\alpha \le 1$, $\delta >0$ and $\chi =\ln (\eta /(1+3\delta ))<\ln {\eta }$ that

$$\begin{aligned} \psi _0 + 2\gamma _0&< 2\psi _0 + 3\gamma _0 = \left( \frac{\eta }{\eta -1}\right) \left( 1-\frac{1}{1+\alpha \chi }\right) \\&\le \left( \frac{\eta }{\eta -1}\right) \left( 1-\frac{1}{1+\ln {\eta }}\right) = \frac{\eta \ln (\eta )}{(\eta -1)(1+\ln {\eta })}. \end{aligned}$$

Note that the right-hand side of the last inequality is a monotonically decreasing function of $\eta \in (1,2]$ (i.e., by looking at its derivative), it then follows from l’Hôpital’s rule that $\psi _0 + 2\gamma _0 <\lim _{\eta \rightarrow 1^+}\frac{\eta \ln (\eta )}{(\eta -1)(1+\ln {\eta })} =\lim _{\eta \rightarrow 1^+}\frac{1+\ln {\eta }}{(1+\ln {\eta }+(\eta -1)/\eta )} =1$.

We assume that *n* is sufficiently large, i.e., $n \ge n_0$ where $n_0$ is defined as in Lemma 49, then that lemma implies

30 $$\begin{aligned} e^{-\chi }&> \left( 1-\frac{\chi }{n}\right) ^n \ge \frac{1+\delta }{1+2\delta } \cdot e^{-\chi }. \end{aligned}$$

We now check the preliminary conditions of Theorem 45. Note that $e^{\chi }/(\eta -1)>1$ since $\chi >0$ and $\eta \le 2$, therefore $\psi _0 = \sqrt{\frac{(2\delta +\varepsilon )e^{\chi }}{\eta -1}}> \sqrt{\varepsilon } > \varepsilon$.

To check whether $(e\chi /r)^r \le \varepsilon \left( 1-\chi /n\right) ^n$, we write it equivalently as

$$\begin{aligned} \left( \frac{r}{e\chi }\right) ^r \ge \frac{1}{\varepsilon \left( 1-\chi /n\right) ^n}. \end{aligned}$$

It follows from $r\ge 8>e^2$, $\chi<\ln {\eta }<\ln {2}<1$, $r\ge 1-\ln {\varepsilon } > \ln {\eta } - \ln {\varepsilon }$, and (30) that indeed the condition holds

$$\begin{aligned} \left( \frac{r}{e\chi }\right) ^r&\ge \left( \frac{e^2}{e}\right) ^r = e^r \ge e^{-\ln {\varepsilon } + \ln {\eta }} = \frac{\eta }{\varepsilon } > \frac{\eta }{\varepsilon }\cdot \frac{1+2\delta }{1+4\delta +3\delta ^2}\\&= \frac{\eta (1+2\delta )}{\varepsilon (1+3\delta )(1+\delta )} = \frac{e^\chi (1+2\delta )}{\varepsilon (1+\delta )} \ge \frac{1}{\varepsilon \left( 1-\chi /n\right) ^n}. \end{aligned}$$

It remains to verify the SM-conditions of the theorem. For $\gamma \le \gamma _0<(1/3)(\delta e^{\chi }/(\eta -1))$, by the monotonicity of *h*, we have

$$\begin{aligned} \frac{\beta (0,\gamma )}{\gamma }&= h(0, \gamma ) \ge h(0, \gamma _0) = \eta - (\eta - 1)\gamma _0\\&> (1+3\delta )e^{\chi } - \frac{\delta e^\chi }{3} > (1+2\delta )e^{\chi } \ge \frac{1+\delta }{\left( 1-\frac{\chi }{n}\right) ^n} \end{aligned}$$

where the last inequality is by (30). Therefore, (SM2a) is satisfied.

Then from (29) and (30), we get for $\gamma \le \gamma _0$ that

$$\begin{aligned} \frac{\beta (\psi _0+\gamma _0,\psi _0+\gamma _0+\gamma )}{\gamma }&= h(\psi _0+\gamma _0, \gamma ) \ge h(\psi _0+\gamma _0, \gamma _0) = \eta - (\eta - 1)(3\gamma _0+2\psi _0)\\&= (1+3\delta )e^{\chi } - (1+3\delta ) e^{\chi } \left( 1-\frac{1}{1+\alpha \chi }\right) > \frac{(1+2\delta )e^{\chi }}{1+\alpha \chi }\\&\ge \frac{1+\delta }{\left( 1-\frac{\chi }{n}\right) ^n (1+\alpha \chi )} \end{aligned}$$

thus (SM2b) holds.

From the definition of $\psi _0$ and the property $\psi _0<1/2$, we have $(\eta -1)\psi _0^2 = (2\delta +\varepsilon )e^{\chi } > (4\delta \psi _0+\varepsilon )e^{\chi }$ and dividing both sides of this with $\psi _0$ gives $(\eta -1)\psi _0 > (4\delta +\varepsilon /\psi _0)e^{\chi }$. Furthermore, our choice of $\varepsilon '$ implies $\delta \ge \varepsilon '$, then for $\gamma \ge \psi _0$ we get

$$\begin{aligned} \frac{\beta (0,\gamma )}{\gamma }&= h(0, \gamma ) \le h(0, \psi _0) = \eta - (\eta -1)\psi _0\\&< (1+3\delta )e^{\chi } - (4\delta +\varepsilon /\psi _0)e^{\chi } = (1 - \delta - \varepsilon /\psi _0)e^{\chi }\\&< \frac{1 - \varepsilon ' - \varepsilon /\psi _0}{\left( 1-\frac{\chi }{n}\right) ^n} \le \frac{1 - \varepsilon ' - \varepsilon /\gamma }{\left( 1-\frac{\chi }{n}\right) ^n} \end{aligned}$$

So, (SM0) is satisfied. Since all parameters we have chosen are constants, (SM3) will hold for a sufficiently large constant *c*. $\square$

The power-law ranking selection mechanism is defined with $\beta (0,\gamma )=\gamma ^c$. Parameter $c \in (0,1)$ controls the strength of the selection, i.e., it is close to uniform selection with *c* close to 0, is close to (1,$\lambda$)-selection if *c* is close to 1. This mechanism was introduced in [14] to show that on easy problems, like the case of the LeadingOnes function, a tight bound on the expected runtime can be computed. On the other hand, on difficult problems like the those in the SLO-hierarchy of [13], polynomial expected runtime can still be achieved for some $\alpha$ and $\varepsilon$.

We now show an analogous result for our Slo$^\alpha _{\varepsilon ,r}$. For this, the following properties of exponential functions from [14]. The mathematical proofs of these results were only available during the review process of that conference, hence we provide them here for the sake of completeness.

#### Lemma 51

(Lemma 15 in [14]). For any $c \in (0,1)$ and any $a,b>0$ holds

$$\begin{aligned} \frac{c}{a^{1-c}}> \frac{(a+b)^c - a^c}{b} > \frac{c}{(a+b)^{1-c}}. \end{aligned}$$

#### Proof

Let $f(x):=x^c$, and note that *f*(*x*) is continuous and differentiable in $[a,a+b]$ with $f'(x)=c/x^{1-c}$. Therefore, by the mean value theorem, there exists a $z \in (a,a+b)$ such that

$$\begin{aligned} \frac{f(a+b) - f(a)}{b} = f'(z). \end{aligned}$$

We also note that $f'(x)$ is monotonically decreasing in $[a,a+b]$ (e.g., $f''(x)=(c-1)c/x^{2-c}<0$ since $c<1$) thus $f'(a)> f'(z) > f'(a+b)$ since $a< z < a+b$. Combining this with the above equation proves our result. $\square$

#### Lemma 52

(Lemma 16 in [14]). If $f(x,y):= \frac{(x+y)^c - x^c}{y}$, $c \in (0,1)$ then ${\frac{\partial f}{\partial x} (x,y) < 0}$ and ${\frac{\partial f}{\partial y} (x,y) < 0}$ for all $x, y >0$.

#### Proof

We have

$$\begin{aligned} \frac{\partial f}{\partial x} (x,y)&= \frac{c}{y}\left( \frac{1}{(x+y)^{1-c}} - \frac{1}{x^{1-c}}\right) , \end{aligned}$$

and note that $x+y>x$, so $(x+y)^{1-c}>x^{1-c}$ since $c<1$, and therefore $\frac{1}{(x+y)^{1-c}} <\frac{1}{x^{1-c}}$ and $\frac{\partial f}{\partial x} (x,y) < 0$.

Also,

$$\begin{aligned} \frac{\partial f}{\partial y} (x,y)&= \frac{1}{y^2}\left( -((x+y)^c - x^c) + \frac{cy}{(x+y)^{1-c}}\right) , \end{aligned}$$

and from Lemma 51 it holds that

$$\begin{aligned} (x+y)^c - x^c > \frac{cy}{(x+y)^{1-c}}, \end{aligned}$$

and this implies $\frac{\partial f}{\partial y} (x,y)<0$. $\square$

#### Lemma 53

(Lemma 17 in [14]). If $a,b\ge 0$, then $(a+b)^c \le a^c + b^c$ for any $c\in (0,1)$.

#### Proof

For $a=b=0$, the result is trivial. For $a+b>0$, the result follows from the concavity of function $f(x):=x^c$ on $[0,+\infty )$ (see e.g., its second derivative in the proof of Lemma 51), because then let $t=a/(a+b)$ we get

$$\begin{aligned} f(a)+f(b)&= f\left( t(a+b)+(1-t)0\right) + f\left( (1-t)(a+b)+t\cdot 0\right) \\&\ge t f(a+b) + (1-t)f(0) + (1-t) f(a+b) + tf(0)\\&= f(a+b).\end{aligned}$$

$$\square $$

The following general result connects $\varepsilon$, $\alpha$ with the parameter *c* of power-law ranking selection, for all problems in Slo$^\alpha _{\varepsilon ,r}$ that can be efficiently solved by Algorithm 1 using that parent selection. As shown in [14] and observed in the experiments [14, 15], unlike previously existed mechanisms, e.g., linear ranking or tournaments, there is a wide range of values for the mutation parameter $\chi$ in which the algorithm is efficient using power-law ranking selection. We therefore do not fix a formulation for the mutation rate, but put it in the same equation for the relation between $\varepsilon , \alpha$ and *c*.

#### Theorem 54

Let $\alpha , \varepsilon \in [0,1]$, $\chi >0$, and $\delta , c \in (0,1)$ be constants such that $\delta \le (1+\alpha \chi )/2$ and

31 $$\begin{aligned} \varepsilon = \left( 1- \frac{1+3\delta }{c(1+\alpha \chi -\delta )}\right) \left( \frac{c(1+\alpha \chi -\delta )e^{-\chi }}{(1+2\delta )}\right) ^{\frac{1}{1-c}}. \end{aligned}$$

Then Algorithm 1 with the power-law ranking selection with parameter *c*, the bitwise mutation with parameter $\chi$, and with population size $\lambda \in {{\,\textrm{poly}\,}}(n)$ such that $\lambda \ge d\ln {n}$ for a sufficiently large constant $d>0$ has polynomial worst case expected runtime on Slo$^\alpha _{\varepsilon ,r}$ for any $r \ge \max \{e^2\chi , \chi - \ln (\varepsilon /2) \}$.

#### Proof

For power-law ranking, we simply have

$$\begin{aligned} \beta (\psi ,\psi +\gamma ) = (\psi +\gamma )^{c} - \psi ^{c}. \end{aligned}$$

Let $h(\psi ,\gamma ) :=\beta (\psi ,\psi +\gamma )/\gamma$, then it follows from Lemma 52 that *h* is decreasing in both $\gamma$ and $\psi$.

We apply Theorem 45 with parameter $\delta$ the same as the one in (31) and with the remaining parameters:

$$\begin{aligned} \varepsilon '&:= \min \left\{ \frac{\delta }{c(1+\alpha \chi - \delta )},\delta ,\frac{2}{3}\right\} ,\\ \psi _0&:=\left( \frac{c(1+\alpha \chi -\delta )e^{-\chi }}{(1+2\delta )}\right) ^{\frac{1}{1-c}},\\ \gamma _0&:= \frac{1}{2}\cdot \left( \frac{c\delta e^{-\chi }}{1+2\delta }\right) ^{\frac{1}{1-c}}. \end{aligned}$$

We first justify that the setting above is appropriate, that is, $\varepsilon '\le \min (\delta ,2/3)$, $\psi _0 + 2\gamma _0 \le 1$ and $\psi _0\ge \gamma _0$ as required by the theorem. The first requirement holds trivially. From $\delta \le (1+\alpha \chi )/2$, we get $\delta \le 1+\alpha \chi -\delta$ and therefore

$$\begin{aligned} 2\gamma _0 = \left( \frac{c\delta e^{-\chi }}{1+2\delta }\right) ^{\frac{1}{1-c}} \le \left( \frac{c(1+\alpha \chi -\delta )e^{-\chi }}{(1+2\delta )}\right) ^{\frac{1}{1-c}}=\psi _0, \end{aligned}$$

or $\gamma _0\le \psi _0/2<\psi _0$. It follows from Lemma 53, and $e^{\chi }\ge 1+\chi$ that

32 $$\begin{aligned} (\psi _0 + 2\gamma _0)^{1-c}&\le \psi _0^{1-c} + (2\gamma _0)^{1-c} = \frac{c(1+\alpha \chi )}{(1+2\delta )e^{\chi }} \nonumber \\&\le \frac{c(1+\alpha \chi )}{(1+2\delta )(1+\chi )} <1 \end{aligned}$$

since $c,\alpha <1$, and $\delta >0$. Thus, $\psi _0 + 2\gamma _0$ is smaller than 1.

We assume that *n* is sufficiently large, i.e., $n \ge n_0$ where $n_0$ is defined as in Lemma 49, then that lemma implies

33 $$\begin{aligned} e^{-\chi }&> \left( 1-\frac{\chi }{n}\right) ^n \ge \frac{1+\delta }{1+2\delta } \cdot e^{-\chi }. \end{aligned}$$

We now check the preliminary conditions of Theorem 45. To check whether $(e\chi /r)^r \le \varepsilon \left( 1-\chi /n\right) ^n$, we write it equivalently as $(r/(e\chi ))^r \ge (1/\left( \varepsilon \left( 1-\chi /n\right) ^n\right)$. It follows from $r\ge \max \{e^2\chi , \chi - \ln (\varepsilon /2)\}$ and (33) that

$$\begin{aligned} \left( \frac{r}{e\chi }\right) ^r&\ge \left( \frac{e^2\chi }{e\chi }\right) ^r = e^r \ge e^{\chi - \ln (\varepsilon /2)} = \frac{e^\chi }{\varepsilon /2} > \frac{(1+2\delta )e^\chi }{(1+\delta )\varepsilon } \ge \frac{1}{\varepsilon \left( 1-\chi /n\right) ^n}. \end{aligned}$$

It remains to verify the SM-conditions of the theorem using the monotonicity of the function *h*. By our choice of $\gamma _0$, $c<1$ and (33) we get

$$\begin{aligned} \frac{\beta (0,\gamma )}{\gamma }&= h(0, \gamma ) \ge h(0, \gamma _0) = \frac{1}{\gamma _0^{1-c}} = 2^{1-c} \cdot \frac{1+2\delta }{c\delta e^{-\chi }} > \frac{1+2\delta }{\delta e^{-\chi }} \ge \frac{1+\delta }{\left( 1-\frac{\chi }{n}\right) ^n}, \end{aligned}$$

and (SM2a) is therefore satisfied.

Then from Lemma 51, (32) and (33), we get for all $\gamma \le \gamma _0$

$$\begin{aligned} \frac{\beta (\psi _0+\gamma _0,\psi _0+\gamma _0+\gamma )}{\gamma }&= h(\psi _0+\gamma _0, \gamma ) \ge h(\psi _0+\gamma _0, \gamma _0)\\&= \frac{(\psi _0+2\gamma _0)^c-(\psi _0+\gamma _0)^c}{\gamma _0}\\&\ge \frac{c}{(\psi _0+2\gamma _0)^{1-c}} \ge \frac{(1+2\delta )e^{\chi }}{1+\alpha \chi } \\&= \frac{(1+\delta )}{(1+\alpha \chi )\cdot \frac{1+\delta }{1+2\delta }\cdot e^{-\chi }} \ge \frac{(1+\delta )}{(1+\alpha \chi ) \left( 1-\chi /n\right) ^n}, \end{aligned}$$

thus (SM2b) holds.

From our choice of $\psi _0$ and (31), we note that $\varepsilon = \left( 1 - \frac{1+3\delta }{c(1+\alpha \chi -\delta )}\right) \psi _0.$ Our choice of $\varepsilon '$ implies that $\frac{\delta }{c(1+\alpha \chi - \delta )}\ge \varepsilon '$, so we have for all $\gamma \ge \psi _0$

$$\begin{aligned} \frac{\beta (0,\gamma )}{\gamma }&= h(0, \gamma ) \le h(0, \psi _0) = \frac{1}{\psi _0^{1-c}} = \frac{\left( \frac{1+2\delta }{c(1+\alpha \chi - \delta )}\right) }{e^{-\chi }}\\&= \frac{1 - \frac{\delta }{c(1+\alpha \chi - \delta )} - \left( 1 - \frac{1+3\delta }{c(1+\alpha \chi - \delta )}\right) }{e^{-\chi }}\\&= \frac{1 - \varepsilon ' - \varepsilon /\psi _0}{e^{-\chi }} \le \frac{1 - \varepsilon ' - \varepsilon /\gamma }{\left( 1-\chi /n\right) ^n}, \end{aligned}$$

and (SM0) is satisfied. Since all parameters we have chosen are constants, (SM3) will hold for a sufficiently large constant *d*. $\square$

## Conclusions

We have introduced the problem hierarchy Slo$^\alpha _{\varepsilon ,r}$ which is a refinement of the problem class SparseLocalOpt. By considering different regimes of $\alpha$ and $\varepsilon$, we get different classifications of problems. The hard part of the hierarchy (those where $\varepsilon >\alpha$) contains deceptive problems, problem classes which are closed under permutation (No Free Lunch theorem), and problems with exponential unrestricted black-box complexity. As we decrease $\varepsilon$ and increase $\alpha$, we get intermediary problem classes that do not contain the hard problems discussed above. The intermediary part of the hierarchy (where $\varepsilon< \alpha < 1$) encompasses some instances of NP-hard problems and randomly perturbed problems. The easiest, most restricted, part of the hierarchy ($\varepsilon$ close to 0 and $\alpha$ close to 1), contains theoretical benchmark problems where many EAs have polynomial runtime.

Our analysis proves a clear benefit of non-elitism when mutation rates and selection pressure are appropriately tuned. The $(\mu +\lambda )$-elitist black-box complexity is exponential even close to the easiest part of the hierarchy, while non-elitist EAs have polynomial expected runtime for broader parts of the hierarchy. While an exponential separation between non-elitist and elitist EAs was proven for the problem BBFunnel [12], we here demonstrate that improvement on the more natural problem class of  OneMax with perturbed fitness. Polynomial expected runtime can be guaranteed even with constant perturbation probability for many non-elitist EAs. This significantly improves the result of [28] which mainly focused on comparing the simple algorithms $(1,\lambda )$ EA and $(1+\lambda )$ EA.

An essential aspect of the Slo$^\alpha _{\varepsilon ,r}$-classification is that it is linked to a partition, thus the following open question is interesting. Does there exist a problem that belongs to both $\textsc{Slo}^{\alpha _1}_{\varepsilon _1,r_1}$ and $\textsc{Slo}^{\alpha _2}_{\varepsilon _2,r_2}$ with $0<\varepsilon _1<\varepsilon _2$ and $\alpha _1<\alpha _2<1$, but not to $\textsc{Slo}^{\alpha _1}_{\varepsilon _2,r_3}$ for some $r_1,r_2,r_3$? Future research should also look into the following directions.

Generalising of the notion of sparsity and density to other search spaces, for example integer values or permutations, will enable a better understanding of problem difficulty in these search spaces. The key observation from our work is that these definitions are tied to the operators used, particularly they guarantee the capability of the operator to escape local optima and explore fitness valleys. In our case, this capability is captured by Lemmas 43 and 44.

It could be interesting to generalise of the notion of sparsity and density beyond the perspective of showing the drawback of elitism. A recent work following this direction appeared in [16] for comparing algorithms in multi-objective optimisation. In that work, the notion of sparsity is introduced to the Pareto front structure, and this leads to a hierarchy of problem difficulties for the simple evolutionary multi-objective algorithm GSEMO [22]. Practical algorithms like NSGA-II, NSGA-III or SMS-EMOA on the other hand are shown being able to overcome these difficulties on some function that is the most difficult for GSEMO.

Our research suggests that the density of fitness valleys and the sparsity of local optima play an important role in determining problem difficulties. While our definitions of (deceptive) local optima and fitness valleys require a level partition, we believe these concepts are well-defined in the context of fitness landscape analysis [33, 39]. Therefore, we believe making the connection from our theoretical work, particularly the notion of sparsity and density and how to estimate them for new functions or practical instances, to fitness landscape analysis with the aim of building better and problem-difficulty-aware algorithms is a fruitful research direction.

## Data Availability

No datasets were generated or analysed during the current study.

## Appendix A: Chernoff Bounds

### Lemma 55

[34] Let $Z_1,\ldots ,Z_n$ be *n* independent Poisson trials such that $\Pr \left( Z_i=1\right) =p_i$. Let $Z=\sum _{i=1}^n Z_i$ and $\mu =E\left[ Z\right]$. Then for $0<\kappa \le 1$,

$$\begin{aligned} \Pr \left( Z>(1+\kappa )\mu \right) \le \exp \left( -\frac{\mu \kappa ^2}{3}\right) . \end{aligned}$$

## Appendix B: Logarithmic Bounds

### Lemma 56

[40] For $$0\le x <\infty $$,

$$\begin{aligned} \frac{2x}{2+x}\le \ln (1+x)\le \frac{x}{2}\cdot \frac{2+x}{1+x} \end{aligned}$$

## Appendix C: The Incompressibility Method

Informally, the Kolmogorov complexity of a natural number *x* conditional on some number *y* is the length of the shortest program *p* running on some universal Turing machine which outputs *x*, when given *y* as input. It is a standard result in this theory that the choice of the universal Turing machine (universal function) is irrelevant up to constant terms.

### Definition 57

[32] Let *x*, *y*, *p* be natural numbers. Any partial recursive function $\phi ,$ together with *p* and *y* such that $\phi (\langle y,p\rangle )=x$, is a *description of x*. The complexity $C_\phi$ of *x* conditional to *y* is defined by

34 $$\begin{aligned} C_\phi (x\mid y) = \min \{\ell (p) \mid \phi (\langle y,p\rangle )=x \}. \end{aligned}$$

For any fixed universal function $\phi _0$, define $C(x\mid y)= C_{\phi _0}(x\mid y)$.

The incompressibility theorem says that any set with *m* elements contains at least one element with Kolmogorov complexity $\Omega (\log m)$. The theorem follows from a simple pigeon hole argument.

### Theorem 58

(Theorem 2.2.1 in [32]). Let *k* be a positive integer. For each fixed *y*, every finite set *A* of cardinality *m* has at least $m(1-2^{-k})$ elements *x* with $C(x\mid y)\ge \log (m)-k$.

The so-called incompressibility method can be a powerful approach to proving a statement *P*. The method consists in showing that if *P* does not hold, then there must exist an encoding of the elements in some set *A* of *m* elements, such that every element has a description of length $o(\log m)$. However, this would contradict the incompressibility theorem, and therefore *P* follows.

## Footnotes

1 The tilde-notation is introduced to disambiguate the levels within regions of BBFunnel, and the partition $(A_1,\ldots ,A_m)$ required by the definition of Slo$^\alpha _{\varepsilon ,r}$.
